# Cross-Coupling of Nitroalkanes with Enamides, Enecarbamates, and Enol Ethers

**DOI:** 10.1021/acs.joc.6c00401

**Published:** 2026-06-09

**Authors:** Katarína R. Detková, Alexandra M. Liptajová, Branislav Ferko, Tomáš Malatinský, Mária Kopáčová, Michal Šoral, Pavol Jakubec

**Affiliations:** a Department of Organic Chemistry, Slovak University of Technology in Bratislava, Radlinského 9, Bratislava 812 37, Slovakia; b Institute of Chemistry, 87280 Slovak Academy of Sciences, Dúbravská cesta 9, Bratislava 845 38, Slovakia

## Abstract

A stepwise cross-coupling of cyclic enamides, enecarbamates, and enol ethers, with functionalized nitroalkanes has been developed, offering access to functionalized tetrahydropyridines, dihydropyrans, and other heterocycles. The outcome of the palladium-free process resembles a Heck-type reaction, in which β-substituted nitroalkanes serve as an acrylate surrogate. The method utilizing the powerful CAN oxidant tolerates several commonly employed protective groups and operates via a well-understood reaction mechanism.

## Graphical abstract

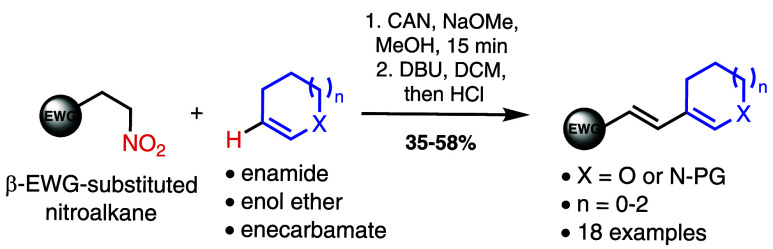

Cross-couplings, as the union of two distinct molecular entities via a covalent bond-forming process, have become one of the most powerful synthetic tools in modern organic synthesis.^1^ Among the most utilized and reliable are transition-metal-catalyzed couplings forming a new σ bond between two sp^2^-hybridized carbon atoms. Their popularity stems partially from their readily accessible cross-coupling partners. The highly popular Heck–Mizoroki cross-coupling employs richly abundant alkenes with aryl (pseudo)­halides or vinyl (pseudo)­halides as reaction partners under Pd catalysis.^2^ Despite their countless admirable features and huge popularity, the necessity to utilize a precious transition-metal catalyst leaves room for further modifications and development of complementary methods. Herein, we report our contribution to the development of novel Pd-free cross-couplings employing nitro compounds as alkene surrogates.^3,4^ The most common variant of the Heck–Mizoroki cross-coupling employs an electron-deficient alkene and aryl halide or vinyl halide as cross-coupling partners.^5^ In the synthesis of conjugated enamides **3a** from ethyl acrylate (**1a**), the vinyl halide is represented by iodoenamide **2a** (Scheme 1, part A).^6,7^ As successfully demonstrated by Gillaizeau, this coupling can proceed directly with methyl acrylate (**1b**) and enamides **2b**, devoid of the halogen.^8^ Similarly, the enol ether **2c** undergoes Pd-catalyzed coupling with methyl vinyl ketone (**1c**) at an elevated temperature, yielding the extended enol ether **3c** (Scheme 1, part B).^9^ We hypothesized that both structural motifs, enamides and enol ethers, and related enecarbamates linked to electron-deficient alkenes could be accessed by a novel, general denitrative cross-coupling of nitroalkanes **4** with enamides, enecarbamates, and enol ethers **2** (Scheme 1, part C). Overall, the process would resemble the Heck coupling but without the need for Pd-catalysis or toxic acrylates.

**Scheme 1:**
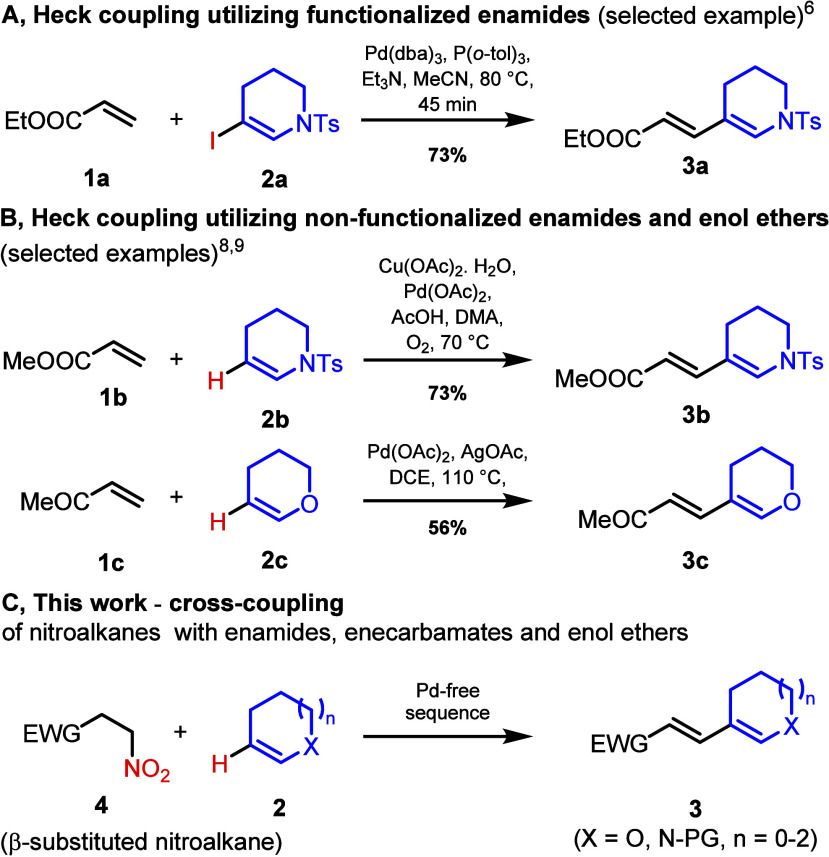
Rationale for the Developed Cross-Coupling

Our idea was built on a previously unexplored application of readily accessible α-nitroalkyl radical **8a** with electron-rich enecarbamates **2d** (Scheme 2, addition) and the ability of the adduct **5a** to undergo an extensive elimination of nitrous acid (elimination, step 1) and methanol (elimination, step 2). α-Nitroalkyl radicals have recently been gaining increasing popularity. Several attractive methods for their preparation became available, particularly due to the work of Ooi et al.^10,11^ Their addition to structurally diverse alkenes paved the way to novel reaction patterns, including various denitrative transformations.^11^ To establish optimal reaction conditions for the envisaged cross-coupling, we have arbitrarily selected nitroalkane **4a** and the Alloc-protected enamine **2d** as the corresponding cross-coupling partners. To generate the key α-nitroalkyl radical **8a**, we adjusted the reaction conditions initially described by Arai and Narasaka^12^ and recently employed in our C-arylation of nitroalkanes.^13,14^ Arai and Narasaka described the CAN oxidation of nitronate to α-nitroalkyl radical in MeOH under cryogenic conditions and its addition to silyl enol ethers.^12^ In the C-arylation, the reaction conditions were tailored to enable a reaction cascade initiated by the addition of an α-nitroalkyl radical to electron-rich heterocycles, and the reaction was performed at room temperature. For the envisioned cross-coupling, generating the α-nitroalkyl radical required further tuning, primarily due to the substituent at the β-position. The reaction temperature was decreased to a convenient 0–5 °C to suppress premature HNO_2_ elimination during the deprotonation of nitroalkane **4a**, resulting in the formation of undesired acrylamide **9** (Scheme 2). The lower temperature is required because the β-substituent is strongly electron-withdrawing and thus promotes HNO_2_ elimination during deprotonation. Sodium methoxide served as a base to fully deprotonate the nitroalkanes **4a**, and readily available cerium ammonium nitrate (CAN) served as a powerful oxidant to oxidize the resulting nitronate **7a** to radical **8a**.^12^ The following cascade of reactions involving enecarbamate **2d**, CAN, and methanol resulted in the formation of intermediate **5a**, thus securing the formation of the key σ bond between the two cross-coupling partners. The subsequent straightforward one-pot elimination of nitrous acid (transformation **5a** to **6a**) and methanol (transformation **6a** to **3d**) was achieved by sequential addition of DBU and hydrogen chloride.^15^ Although an extractive aqueous workup was necessary after the first step, the two-pot cross-coupling gave a respectable 43% isolated yield of tetrahydropyridine **3d** after a single chromatographic purification. The isolated yield of **3d** over three steps correlates well with the isolated yields after each step. Nitro amide **5a** containing three stereocenters was isolated in 52% yield as a mixture of racemic diastereomers, while the yield of acrylamide **6a** was 45% over two steps (addition and elimination, step 1). Pleased with the simple reaction setup and short reaction times of the novel cross-coupling, we continued with the reaction scope investigation. First, the scope of the electron-rich alkene partner was investigated in the reaction with nitro amide **4a** (Scheme 3). A range of five, six, and seven-membered ring enecarbamates and enamides **2** bearing various functional groups at the nitrogen atom were reacted with nitro amide **4a** under established reaction conditions. Intermediates **5** were subsequently treated with DBU and HCl to yield the desired products **3**. Pleasingly, common protective groups such as Alloc, Boc, Cbz, Ts, and Bn were all well tolerated during both stages of the coupling, and the desired products **3d**-**3j** were isolated as exclusive *E*-isomers in respectable yields. Taking into account the powerful oxidative properties of CAN (*E*° = 1.61 V vs NHE)^16^ and the complexity of the overall process (see Scheme 5 for a mechanistic proposal), the developed cross-coupling represents an example of a high level of chemo- and stereoselectivity. Apart from the cyclic enecarbamates and enamides, the procedure can even be applied to enol ethers, as demonstrated by the preparation of dihydropyrans **3k** and **3l**. Having explored the scope of electron-rich alkenes, our focus turned to nitroalkanes.

**Scheme 2:**
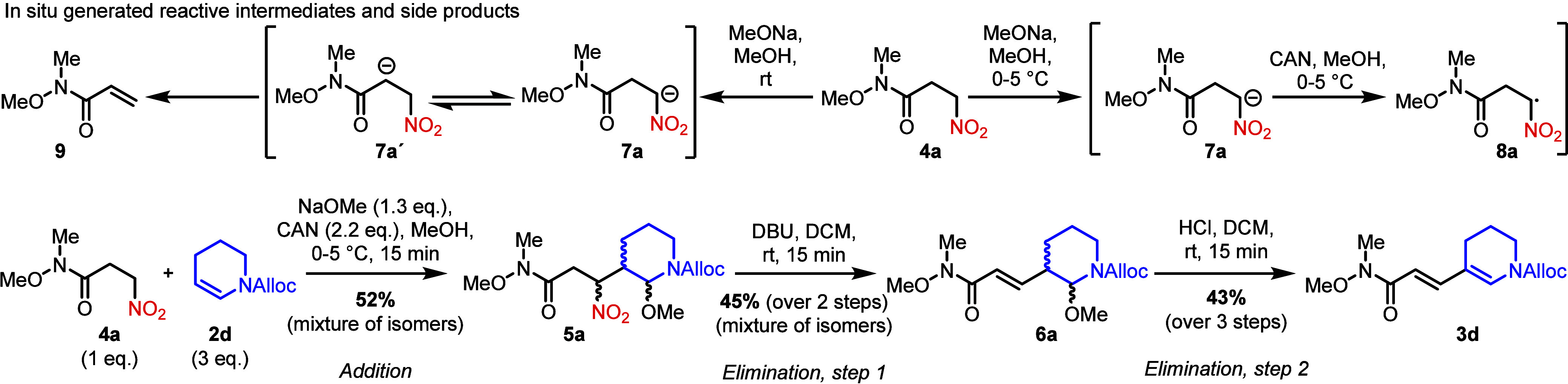
Proof of Concept

**Scheme 3:**
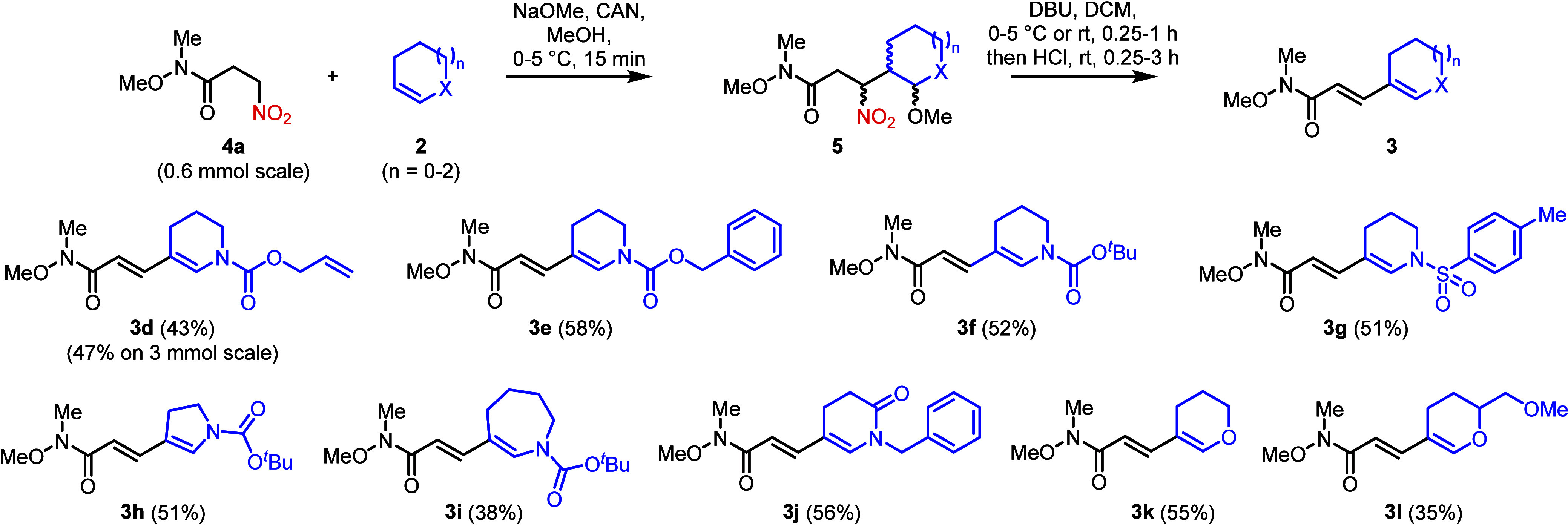
Scope of Denitrative Cross-Coupling with a Range of Enecarbamates, Enamides, and Enol Ethers

Various nitroalkanes bearing an electron-withdrawing group in the β-position were tested (Scheme 4). Methyl, ethyl, and benzyl ester-containing products **3m**–**3r** were successfully prepared and isolated in moderate to good chemical yields. The modularity of the acidifying group in the β-position of nitroalkanes was further demonstrated by the preparation of tetrahydropyridines **3s** and **3t** bearing a dimethylamide and phosphonate functionality. Initially, experiments employing a selected nitroalkane with a sulfone moiety in the β-position met with limited success, and the desired product **3u** was not detected in the crude reaction mixtures. Luckily, the root of the problem was quickly identified – premature elimination of HNO_2_ at the standard reaction temperature (0–5 °C). Consequently, the reaction temperature was adjusted, and the method could eventually be extended to nitroalkanes bearing a *tert*-butylsulfone in the β-position, yielding tetrahydropyridine **3u** in 53% yield. As anticipated, lowering the acidity of the β-position next to the nitro group revealed certain limitations of the novel coupling. When 2-phenyl-1-nitroethane reacted with dihydropyran under standard conditions for the addition step, nitroacetal **5v** was isolated in 56% yield (Scheme 4). However, all attempts (thermal elimination in refluxing toluene and utilization of a stronger base, MTBD instead of DBU) to proceed with the elimination of HNO_2_ failed, and only unreacted starting material **5v** was recovered. This limitation can be attributed to the increased p*K*_a_ of the β-position, which hampers efficient deprotonation by DBU. Further limitation was revealed when the electron-rich alkene was replaced by cyclohexene in the reaction with 2-phenyl-1-nitroethane. No desired adduct **5w** was detected in the crude reaction mixture (Scheme 4). Despite these limitations, the developed cross-coupling offers a Pd-free alternative to the existing Mizoroki-Heck coupling for the synthesis of certain structural motifs.

**Scheme 4:**
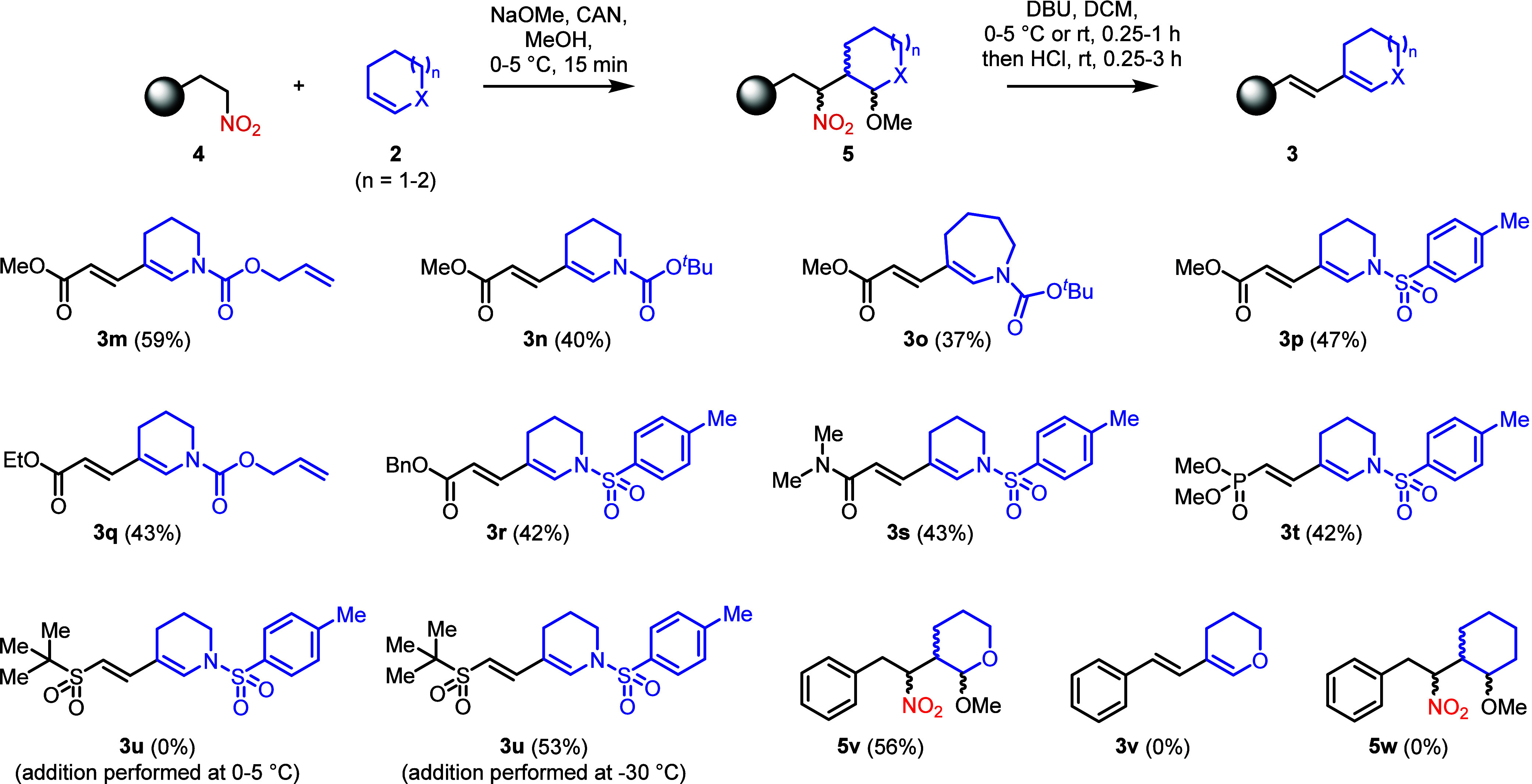
Scope of the Denitrative Cross-Coupling with a Range of β-Substituted Nitroalkanes

With strong experimental evidence for multiple intermediates at various stages of the cross-coupling, we propose that the two-pot process proceeds via the mechanism shown in Scheme 5. Initially, nitroalkane **4** is deprotonated by sodium alkoxide, yielding nitronate **7**, which is then oxidized to nitroalkyl radical **8** by CAN. Subsequent radical addition to the electron-rich double bond of dihydropyran yields stabilized radical **10**, which is oxidized to the carbenium ion **11**. The nucleophilic addition of the methoxide, forming isolable adduct **5**, completes the addition step of the cross-coupling. The elimination of HNO_2_ is proposed to proceed via an E1cb-type mechanism involving the intermediate **12**. The protonation of acetal **6** triggers the final methanol elimination. The proposed formation of radical **8** and its subsequent addition were supported by a radical scavenger experiment in which TEMPO adduct **16** was isolated instead of adduct **5k**. Also, the proposed mechanism is consistent with mechanisms recently reported by our group for the C-arylation of nitroalkanes.^13^

**Scheme 5:**
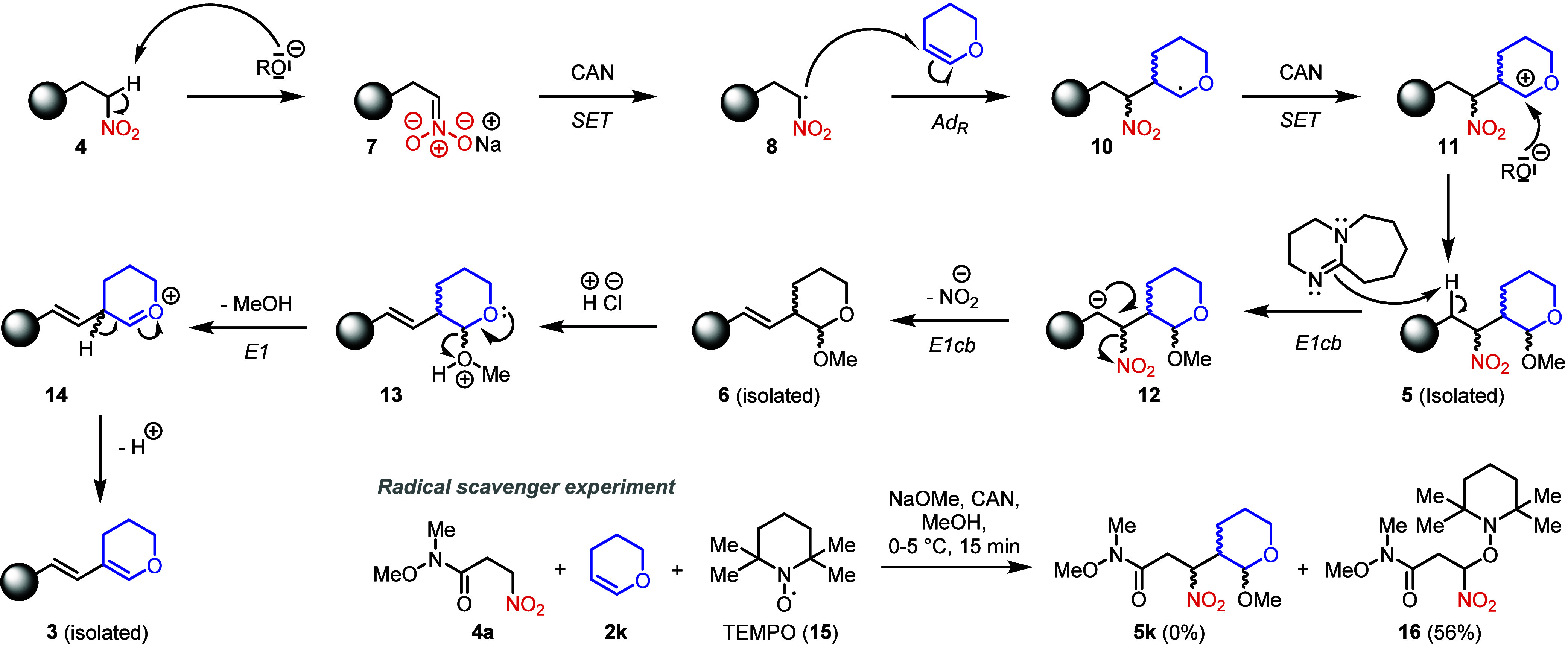
Proposed Reaction Mechanism

In summary, we have developed a denitrative cross-coupling reaction between nitro compounds bearing an electron-withdrawing substituent at the β-position and a series of cyclic electron-rich alkenes. The discovered protocol operates under mild conditions and short reaction times, using readily available cross-coupling partners, and allows straightforward access to functionalized tetrahydropyridines, dihydropyrans, and related heterocycles.

## Supplementary Material



## Data Availability

The data underlying this study are available in the published article and its Supporting Information.

## Notes

Experimental procedures, characterization data, and ^1^H and ^13^C NMR spectra (PDF)
