# Supplementary material for: Cross-Coupling of Nitroalkanes with Enamides, Enecarbamates, and Enol Ethers
Source: J Org Chem. 2026 Jun 9;91(24):8440–4. doi: 10.1021/acs.joc.6c00401 (PMC13288628; doi:10.1021/acs.joc.6c00401)
Supplement: Supplementary file 1 [file jo6c00401_si_001.pdf]

Supporting Information

Cross-Coupling of Nitroalkanes with Enamides, Enecarbamates, and Enol Ethers

Katarína R. Detková,[a] Alexandra M. Liptajová,[a] Branislav Ferko,[a] Tomáš Malatinský,[a] Mária Kopáčová,[b] Michal Šoral,[b] and Pavol Jakubec[a*]

[a] Department of Organic Chemistry, Slovak University of Technology in Bratislava, Radlinského 9, 812 37 Bratislava, Slovakia

[b] Institute of Chemistry, Slovak Academy of Sciences, Dúbravská cesta 9, 845 38 Bratislava, Slovakia

Contents

1. General experimental and methods S6

1.1 Reagents and solvents S6

1.2 Chromatography S6

1.3 Spectroscopy and instruments S6

1.4 Starting materials S7

2. Practical experimental S8

2.1 Preparation and characterization of nitroalkanes and enecarbamate S8

2.1.1 Benzyl 3-nitropropanoate (4d) S8

2.1.2 Synthesis and characterization of nitroalkane 4e S8

2.1.2.1 3-Nitropropionic acid chloride 20 .... S8

2.1.2.2 3-Nitro-*N*,*N*-dimethylpropanamide (4e) S9

2.1.3 Dimethyl (2-nitroethyl)phosphonate (4f) S9

2.1.4 Synthesis of enecarbamate 2d S10

2.1.4.1 Allyl 3,4-dihydropyridine-1(2*H*)-carboxylate (2d) S10

2.2 Optimization study S11

2.2.1. Optimization - The addition step S11

2.2.2 Optimization – The addition and elimination steps S11

2.3 Synthesis and characterization of intermediates in the denitrative cross-coupling S12

2.3.1 Allyl 2-methoxy-3-(3-(methoxy(methyl)amino)-1-nitro-3-oxopropyl)piperidine-1-carboxylate (5a) S12

2.3.2 (*E*)-Allyl 2-methoxy-3-(3-(methoxy(methyl)amino)-3-oxoprop-1-en-1-yl)piperidine-1-carboxylate (6a) S12

2.4 Synthesis and characterization of the denitrative cross-coupling products S13

2.4.1 General procedure A S13

2.4.2 Allyl (*E*)-5-(3-(methoxy(methyl)amino)-3-oxoprop-1-en-1-yl)-3,4-dihydropyridine-1(2*H*)-carboxylate (3d) S14

2.4.2.1 Synthesis of compound 3d on 3 mmol scale S14

2.4.3 Benzyl (*E*)-5-(3-(methoxy(methyl)amino)-3-oxoprop-1-en-1-yl)-3,4-dihydropyridine-1(2*H*)-carboxylate (3e) S15

2.4.4 *tert*-Butyl (*E*)-5-(3-(methoxy(methyl)amino)-3-oxoprop-1-en-1-yl)-3,4-dihydropyridine-1(2*H*)-carboxylate (3f) S15

2.4.5 (*E*)-*N*-methoxy-*N*-methyl-3-(1-tosyl-1,4,5,6-tetrahydropyridin-3-yl)acrylamide (3g) S16

2.4.6. *tert*-Butyl (*E*)-4-(3-(methoxy(methyl)amino)-3-oxoprop-1-en-1-yl)-2,3-dihydro-1*H*-pyrrole-1-carboxylate (3h) S17

2.4.7 *tert*-Butyl (*E*)-6-(3-(methoxy(methyl)amino)-3-oxoprop-1-en-1-yl)-2,3,4,5-tetrahydro-1*H*-azepine-1-carboxylate (3i) S17

2.4.8 (*E*)-3-(1-Benzyl-6-oxo-1,4,5,6-tetrahydropyridin-3-yl)-*N*-methoxy-*N*-methylacrylamide (3j). S18

2.4.9 (*E*)-3-(3,4-Dihydro-2*H*-pyran-5-yl)-*N*-methoxy-*N*-methylacrylamide (3k).......... S19

2.4.10 (*E*)-*N*-Methoxy-3-(2-(methoxymethyl)-3,4-dihydro-2*H*-pyran-5-yl)-*N*-methylacrylamide (3l) S19

2.4.11 Allyl (*E*)-5-(3-methoxy-3-oxoprop-1-en-1-yl)-3,4-dihydropyridine-1(2*H*)-carboxylate (3m) S20

2.4.12 *tert*-Butyl (*E*)-5-(3-methoxy-3-oxoprop-1-en-1-yl)-3,4-dihydropyridine-1(2*H*)-carboxylate (3n) S21

2.4.13 *tert*-Butyl (*E*)-6-(3-methoxy-3-oxoprop-1-en-1-yl)-2,3,4,5-tetrahydro-1*H*-azepine-1-carboxylate (3o) S21

2.4.14 Methyl (*E*)-3-(1-tosyl-1,4,5,6-tetrahydropyridin-3-yl)acrylate (3p) S22

2.4.15 Allyl (*E*)-5-(3-ethoxy-3-oxoprop-1-en-1-yl)-3,4-dihydropyridine-1(2*H*)-carboxylate (3q) S23

2.4.16 Benzyl (*E*)-3-(1-tosyl-1,4,5,6-tetrahydropyridin-3-yl)acrylate (3r) S23

2.4.17 (*E*)-*N*,*N*-Dimethyl-3-(1-tosyl-1,4,5,6-tetrahydropyridin-3-yl)acrylamide (3s) S24

2.4.18 (*E*)-Dimethyl (2-(1-tosyl-1,4,5,6-tetrahydropyridin-3-yl)vinyl)phosphonate (3t) S25

2.4.19 (*E*)-5-(2-(tert-Butylsulfonyl)vinyl)-1-tosyl-1,2,3,4-tetrahydropyridine (3u) S26

2.4.20 (*E*)-5-Styryl-3,4-dihydro-2*H*-pyran (3v) S26

2.4.20.1 2-Methoxy-3-(1-nitro-2-phenylethyl)tetrahydro-2*H*-pyran (5v) S27

2.4.20.2 (*E*)-2-Methoxy-3-styryltetrahydro-2*H*-pyran (6v) S28

2.4.21.1 1-Methoxy-2-(1-nitro-2-phenylethyl)cyclohexane (5w) S28

3. Mechanistic investigation S29

3.1 Experiment in the presence of radical scavenger TEMPO S29

3.1.1 2,2,6,6-Tetramethyl-1-(1-nitro-2-phenylethoxy)piperidine (16) S29

3.2 HPLC analysis of the addition phase, transformation 4a to 5a S30

4. ^{1}H and $^{13}C\{^{1}H\}$ NMR spectra S31
4.1 ^{1}H and $^{13}C\{^{1}H\}$ NMR spectra of 4d S31
4.2 ^{1}H and $^{13}C\{^{1}H\}$ NMR spectra of 20 ..... S32
4.3 ^{1}H and $^{13}C\{^{1}H\}$ NMR spectra of 4e S33
4.4 ^{1}H, $^{13}C\{^{1}H\}$ and ^{31}P NMR spectra of 4f S34
4.5 ^{1}H and $^{13}C\{^{1}H\}$ NMR spectra of 2d S36
4.6 ^{1}H and $^{13}C\{^{1}H\}$ NMR spectra of 5a S37
4.7 ^{1}H and $^{13}C\{^{1}H\}$ NMR spectra of 6a S38
4.8 ^{1}H and $^{13}C\{^{1}H\}$ NMR spectra of 3d S39
4.9 ^{1}H and $^{13}C\{^{1}H\}$ NMR of 3e S40
4.10 ^{1}H and $^{13}C\{^{1}H\}$ NMR spectra of 3f S41
4.11 ^{1}H and $^{13}C\{^{1}H\}$ NMR spectra of 3g S42
4.12 ^{1}H and $^{13}C\{^{1}H\}$ NMR spectra of 3h S43
4.13 ^{1}H and $^{13}C\{^{1}H\}$ NMR spectra of 3i S44
4.14 ^{1}H and $^{13}C\{^{1}H\}$ NMR spectra of 3j S45
4.15 ^{1}H and $^{13}C\{^{1}H\}$ NMR spectra of 3k S46
4.16 ^{1}H and $^{13}C\{^{1}H\}$ NMR spectra of 3l S47
4.17 ^{1}H and $^{13}C\{^{1}H\}$ NMR spectra of 3m S48
4.18 ^{1}H and $^{13}C\{^{1}H\}$ NMR spectra of 3n S49
4.19 ^{1}H and $^{13}C\{^{1}H\}$ NMR spectra of 3o S50
4.20 ^{1}H and $^{13}C\{^{1}H\}$ NMR spectra of 3p S51
4.21 ^{1}H and $^{13}C\{^{1}H\}$ NMR spectra of 3q S52
4.22 ^{1}H and $^{13}C\{^{1}H\}$ NMR spectra of 3r S53
4.23 ^{1}H and $^{13}C\{^{1}H\}$ NMR spectra of 3s S54
4.24 ^{1}H, $^{13}C\{^{1}H\}$ NMR and ^{31}P spectra of 3t S55
4.25 ^{1}H and $^{13}C\{^{1}H\}$ NMR spectra of 3u S57
4.26 ^{1}H and $^{13}C\{^{1}H\}$ NMR spectra of 5v S58
4.27 ^{1}H and $^{13}C\{^{1}H\}$ NMR spectra of 16 ..... S59
5. References S60

1. General experimental and methods

All reactions were performed without special precautions to avoid the presence of moisture unless otherwise stated.

1.1 Reagents and solvents

Commercially available reagents and solvents were used without further purification. They were obtained from commercial suppliers (Sigma Aldrich, Fluorochem, Abcr, Acros Organics). Acetonitrile used for the hydrodesulfurization was purchased from Acros Organics (99.9%, Extra Dry over Molecular Sieve, AcroSeal™). Solvents were removed under reduced pressure using Büchi Rotavapor and Heidolph apparatus.

1.2 Chromatography

Column chromatography was carried out using silica gel: Merck Kieselgel 60 (15 - 40 µm). All reactions were monitored by thin-layer chromatography (TLC - Merck Kieselgel 60 F254 (230 - 400 mesh) fluorescent treated silica). For practical use, it was visualized under UV light (254 nm) or by staining with an aqueous solution of basic potassium permanganate.

Reverse-phase HPLC used to monitor reaction progress was operated with the following parts: Hardware: E-COM machine setup using ECD2000 detector series. Software: Clarity. Columns: Kromasil 100 C18, 5 µm (MZ-Analysentechnik GmbH, 125 × 4.6 mm). Mobile phases: Gradient using 0.1% aqueous HCOOH and acetonitrile.

1.3 Spectroscopy and instruments

^{1}H and ^{13}C NMR spectra were recorded on spectrometers (Bruker 400 MHz and Varian Inova-300 and Varian VNMRS-600), operating at frequencies of 300 MHz, 400 MHz and 600 MHz (for ^{1}H nuclei) and 75 MHz, 101 MHz and 151 MHz (for ^{13}C nuclei). The resonance frequency of tetramethylsilane (TMS) served as a reference frequency for the calculation of the chemical shift scales ($\delta_H = \delta_C = 0.000$ ppm). For the ^{1}H spectra, the residual non-perdeuterated solvent signal ($CHCl_3$: $\delta_H = 7.260$ ppm) was used as a secondary reference whilst correctly shifting the chemical shift scale. For the ^{13}C spectra, the deuterated solvent signal ($CDCl_3$: $\delta_C = 77.16$ ppm) was used analogously. Chemical shifts (δ) are reported in parts per million (ppm), and coupling constants (J) are given in Hertz (Hz). The ^{1}H NMR spectra are reported as follows: δ/ppm [number of protons, multiplicity (s = singlet, d = doublet, t = triplet, q = quartet, quin = quintet, m = multiplet, b = broad), coupling constants J/Hz (where appropriate)]. Data were analyzed using the Mestrenova program. All NMR spectra and measurement

parameters are available upon request from the corresponding author. High-resolution mass spectra (HRMS) were recorded on Orbitrap Elite (Thermo Fisher Scientific). The following measurement conditions were used: ion source HESI (heated electrospray), capillary temperature 350 ºC, source heater temperature 300 ºC, full scan, positive polarity, resolution 120000, spray voltage 4kV (APPI/APCI both temperatures 290 ºC).

1.4 Starting materials

Starting materials utilized in the article, which are not commercially available from major worldwide suppliers, were prepared either according to referenced literature procedures or newly developed procedures described in the text below.

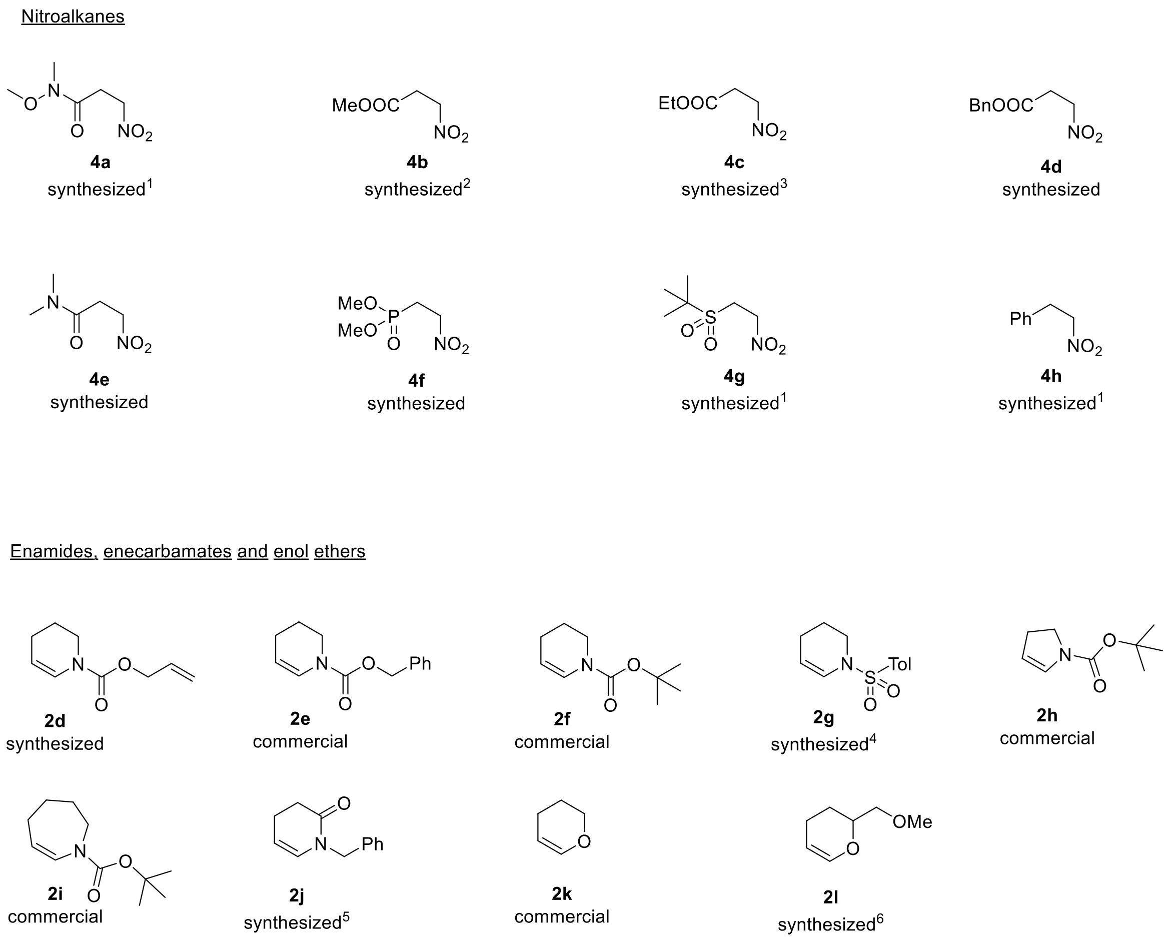

Figure S1

2. Practical experimental

2.1 Preparation and characterization of nitroalkanes and enecarbamate

2.1.1 Benzyl 3-nitropropanoate (4d)

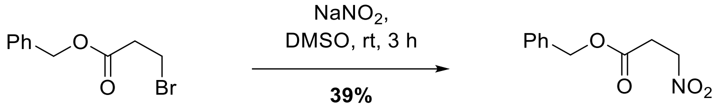

18 **4d**

Scheme S1

To a mixture of bromoester **18**[7] (10.00 mmol, 2.431 g) and DMSO (50 mL) was added sodium nitrite (2 eq., 20.0 mmol, 1.38 g), and the resulting mixture was left stirring at rt. After 3 h, the mixture was diluted with water (200 mL, pre-cooled to 4 °C) and extracted with EtOAc (3 × 50 mL). The combined organics were washed with brine (50 mL), dried (Na_2SO_4), and concentrated *in vacuo*, yielding a yellow heterogeneous residue (2.4 g). This residue was purified by column chromatography (gradient eluent hexane:EtOAc 90:10 to 85:15), yielding ester **4d** as a white solid (0.81 g, 39%).

^{1}H NMR (300 MHz, $CDCl_3$) δ 7.42 – 7.31 (m, 5H), 5.18 (s, 2H), 4.69 – 4.64 (m, 2H), 3.06 – 3.01 (m, 2H). **^{13}C{^{1}H} NMR** (75 MHz, $CDCl_3$) δ 169.4, 135.3, 128.8, 128.7, 128.5, 69.8, 67.4, 31.3. **HRMS** (*m/z*): $[M+Na]^+$ calcd for $C_{10}H_{11}NO_4Na^+$, 232.0580; found, 232.0582.

2.1.2 Synthesis and characterization of nitroalkane 4e

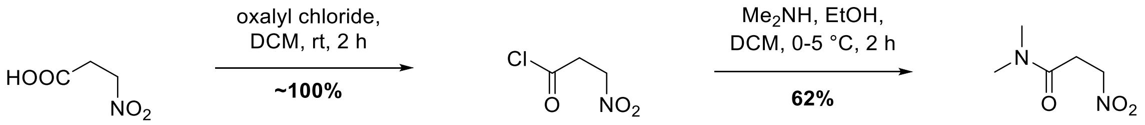

19 **20** **4e**

Scheme S2

2.1.2.1 3-Nitropropionic acid chloride 20

To a mixture of acid **19** (8.00 mmol, 0.953 g) and DCM (40 mL) was added oxalyl chloride (1.5 eq., 12 mmol, 1.5 g, 1.0 mL) and DMF (0.02 eq., 0.16 mmol, 12 mg, 12 µL). The resulting mixture was stirred at rt with a bubbler attached. After 2 h, the mixture was concentrated *in vacuo*, yielding the acid chloride **20** (1.1 g, 100%) as a pale-orange oil.

^{1}H NMR (600 MHz, $CDCl_3$) δ 4.67 (t, J = 5.8 Hz, 1H), 3.56 (t, J = 5.8 Hz, 1H). **^{13}C{^{1}H} NMR** (151 MHz, $CDCl_3$) δ 171.0, 69.0, 42.8. **HRMS -** The molecular ion was not detected using the method described in Section 1.

2.1.2.2 3-Nitro-*N*,*N*-dimethylpropanamide (4e)

To the crude acid chloride **20** (8.0 mmol, 1.1 g) was added DCM (40 mL), and the resulting mixture was cooled to 0-5 °C (ice-bath) under argon. Dimethylamine (4 eq., 32 mmol, 5.7 mL of 5.6 M solution in EtOH) was added, and the mixture was stirred at 0-5 °C (ice-bath) under argon. After 2 h the mixture was diluted with 1 M aqueous HCl (50 mL) and extracted with EtOAc (5 × 50 mL), the combined organics were washed with brine (50 mL), dried (Na_2SO_4) and concentrated *in vacuo* yielding a pale-orange liquid (1.1 g). This residue was purified by column chromatography (gradient eluent hexane:EtOAc 50:50 to EtOAc), affording amide **4e** (0.73 g, 62%) as a pale-yellow liquid.

^{1}H NMR (300 MHz, $CDCl_3$) δ 4.71 – 4.66 (m, 2H), 3.04 (s, 3H), 3.00 – 2.95 (m, 5H). **^{13}C{^{1}H} NMR** (151 MHz, $CDCl_3$) δ 168.3, 70.4, 37.1, 35.6, 30.1. **HRMS** (*m/z*): $[M+H]^+$ calcd for $C_5H_{11}N_2O_3^+$, 147.0764; found, 147.0767.

2.1.3 Dimethyl (2-nitroethyl)phosphonate (4f)

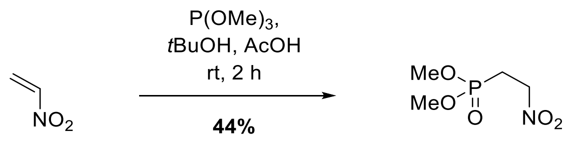

21 4f

Scheme S3

The synthesis of nitrophosphonate **4f** was performed according to the reported procedure.[8] Nitroethene **21**[9] (13.0 mmol, 0.950 g) was added dropwise to a solution of trimethoxyphosphine (5 eq., 65 mmol, 8.1 g, 7.7 mL) and acetic acid (4 eq., 52 mmol, 3.1 g, 3.0 mL) in *t*BuOH (33 mL) under argon. The resulting mixture was stirred at rt under argon. After 2 h, the mixture was concentrated *in vacuo*, yielding a pale-yellow oil (1.5 g). This residue was purified by column chromatography (gradient eluent hexane:EtOAc 90:10 to 50:50 to EtOAc), affording nitrophosphonate **4f** (1.05 g, 44%) as a pale-yellow oil.

^{1}H NMR (300 MHz, $CDCl_3$) δ 4.64 – 4.47 (m, 2H), 3.76 (d, J = 11.0 Hz, 6H), 2.58 – 2.41 (m, 2H). **^{13}C{^{1}H} NMR** (75 MHz, $CDCl_3$) δ 69.2, 53.0 (d, J = 6.3 Hz), 23.2 (d, J = 143.7 Hz). **^{31}P NMR** (243 MHz, $CDCl_3$) δ 26.7. **HRMS** (*m/z*): $[M+H]^+$ calcd for $C_4H_{11}NO_5P^+$, 184.0369; found, 184.0374. The data are in good agreement with previously published data.[10]

2.1.4 Synthesis of enecarbamate 2d

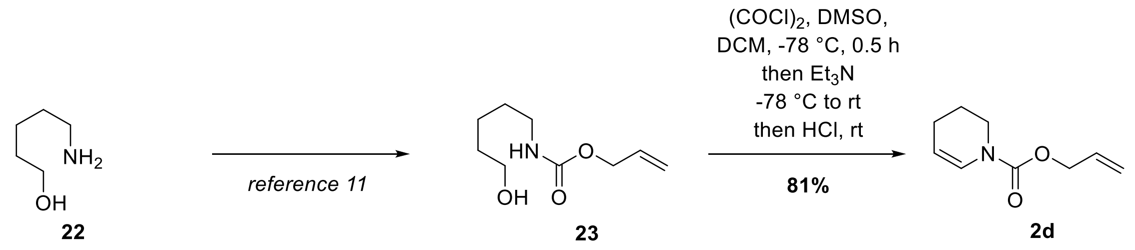

Scheme S4

2.1.4.1 Allyl 3,4-dihydropyridine-1(2*H*)-carboxylate (2d)

A mixture of oxalyl chloride (1.2 eq., 3.97 mmol, 0.504 g, 0.341 mL) and DCM (12 mL) was cooled to -78 °C (a dry ice-acetone bath) under argon. DMSO (2.1 eq., 6.95 mmol, 0.543 g, 0.494 mL) was added dropwise at -78 °C. After 5 min, a solution of carbamate **23** (3.31 mmol, 0.620 g) in DCM (3 mL) was added, and the resulting mixture was stirred at -78 °C (a dry ice-acetone bath) under argon. After 0.5 h Et_3N (5 eq., 17 mmol, 1.7 g, 2.3 mL) was added dropwise, the mixture was warmed to rt and stirred at rt under argon. After 0.5 h, 3 M aqueous HCl solution (13 mL) was added, and the mixture was stirred at rt. After 10 min the phases were separated and the aqueous phase was extracted with DCM (10 mL). The combined organics were washed with water (10 mL), dried (Na_2SO_4), and concentrated *in vacuo,* yielding a pale-yellow oil. This residue was purified by column chromatography (gradient eluent hexane to hexane:EtOAc 90:10 to 85:15), yielding enecarbamate **2d** (0.45 g, 81%) as a pale-yellow liquid.

NMR spectra of compound ***2d*** *show a rotameric mixture and are reported as observed*
^{1}H NMR (300 MHz, $CDCl_3$) δ 6.88 – 6.71 (m, 1H), 5.93 (ddt, J = 17.2, 10.4, 5.5 Hz, 1H), 5.36 – 5.15 (m, 2H), 4.96 – 4.82 (m, 1H), 4.62 (dt, J = 5.6, 1.5 Hz, 2H), 3.64 – 3.54 (m, 2H), 2.02 (m, 2H), 1.88 – 1.74 (m, 2H). **^{13}C{^{1}H} NMR** (75 MHz, $CDCl_3$) δ 153.5, 153.1, 132.8, 125.4, 124.9, 117.8, 106.7, 106.4, 66.4, 66.3, 42.4, 42.2, 21.7, 21.5, 21.3. **HRMS** (*m/z*): $[M+H]^+$ calcd for $C_9H_{14}NO_2^+$, 168.1019; found, 168.1023. The data are in good agreement with previously published data.[12]

2.2 Optimization study

2.2.1. Optimization - The addition step

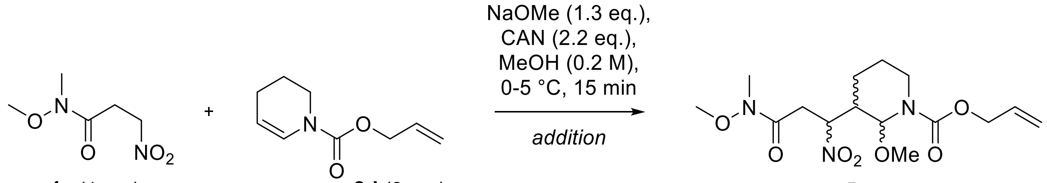

4a (1 eq.) **2d** (3 eq.) **5a**

Scheme S5

The utilized reaction conditions for the addition step were adopted from the extensively optimized C-arylation of nitroalkanes.[13] The reaction temperature was decreased to 0-5 °C to suppress premature elimination of HNO_2 during the deprotonation of nitroalkane **4a**.

2.2.2 Optimization – The addition and elimination steps

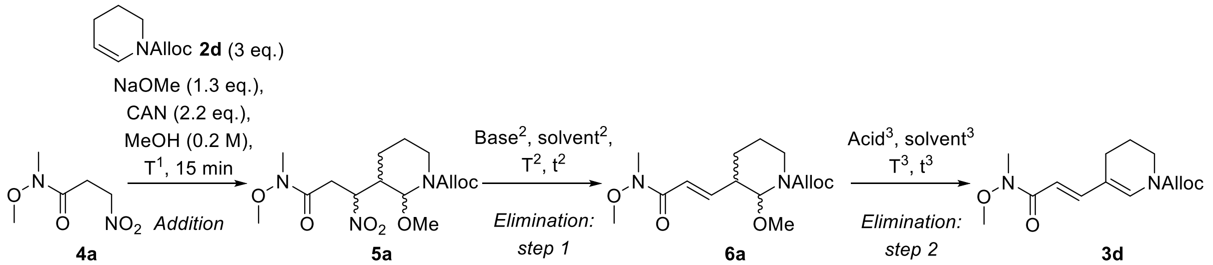

4a **5a** **6a** **3d**

Entry	Addition	Elimination: Step 1				Elimination: Step 2				
	T^1	$Base^2$	$Solvent^2$	T^2	t^2	$Acid^3$	$Solvent^3$	T^3	t^3	Yield of **3d**
1	-20 °C	DBU	DCM	rt	15 min	HCl (2 eq.)	DCM	rt	15 min	38%[a]
2	0-5 °C	DBU	DCM	rt	15 min	HCl (2 eq.)	DCM	rt	15 min	43%[a] (43%)[b]
3[c]	0-5 °C	DBU	DCM	rt	15 min	HCl (2 eq.)	DCM	rt	15 min	12%[a]
4	rt	DBU	DCM	rt	15 min	HCl (2 eq.)	DCM	rt	15 min	18%[a]
5	0-5 °C	*t*BuOK	THF	0-5 °C	1 h	-	DCM	-	-	9%[a]
6	0-5 °C	DBU	DCM	rt	1 h	-	DCM	-	-	24%[a]
7	0-5 °C	DBU	DCM	rt	1 h	-	DCM	-	-	24%[a]
8	0-5 °C	*t*BuOK	THF	0-5 °C-rt	1 h	-	DCM	-	-	15%[a]
9	0-5 °C	DBU	DCM	rt	1 h	-	DCM	-	-	26%[a]
10	0-5 °C	DBU	DCM	rt	1 h	HCl (5 eq.)	DCM	rt	1 h	35%[a]
11	0-5 °C	DBU	DCM	rt	15 min	HCl (3 eq.)	DCM	rt	15 min	40%[a]
12	0-5 °C	DBU	DCM	rt	1 h	HCl (3 eq.)	DCM	rt	1 h	33%[a]
13	0-5 °C	DBU	DCM	rt	15 min	HCl (2 eq.)	DCM	rt	15 min	43%[a]
14	0-5 °C	DBU	DCM	0-5 °C	15 min	HCl (2 eq.)	DCM	0-5 °C	15 min	23%[a]

[a] NMR yield [b] Isolated yields [c] Reaction with an inverted stoichiometry – **2d** (1 eq.), **4a** (3 eq.), NaOMe (3 eq.)

Figure S2

2.3 Synthesis and characterization of intermediates in the denitrative cross-coupling

2.3.1 Allyl 2-methoxy-3-(3-(methoxy(methyl)amino)-1-nitro-3-oxopropyl)piperidine-1-carboxylate (5a)

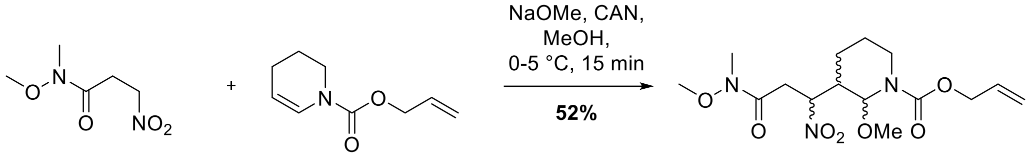

4a **2d** **5a**

Scheme S6

To a mixture of nitroalkane **4a** (0.60 mmol, 0.097 g) in MeOH (2 mL) cooled to 0-5 °C (ice-bath) was added MeONa (1.3 eq., 0.78 mmol, 0.17 g of 25% w/w solution in MeOH) immediately followed by the addition of solution of alkene **2d** (3.0 eq., 1.80 mmol, 0.301 g) in MeOH (1 mL) and solution of CAN (2.2 eq., 1.30 mmol, 0.724 g) in MeOH (3 mL). The resulting mixture was vigorously stirred (500 rpm) at 0-5 °C. After 15 minutes, a saturated aqueous solution of $Na_2S_2O_3$ (10 mL) and water (10 mL) were added, and the mixture was extracted with EtOAc (2 × 10 mL). The combined organic phases were washed with brine (10 mL), dried (Na_2SO_4), and concentrated *in vacuo*. This residue was purified by column chromatography (gradient eluent hexane to hexane:EtOAc 90:10 to 85:15), yielding the nitro compound **5a** (0.112 g, 52%) as a colorless oil.

^{1}H NMR ($CDCl_3$) and **$^{13}C\{^1H\}$ NMR** ($CDCl_3$): Due to the complexity of compound **5a** containing multiple diastereomers, the individual signals are not listed. However, the recorded NMR spectra are available in Section 4 on p. S37. **HRMS** (*m/z*): $[M+Na]^+$ calcd for $C_{15}H_{25}N_3NaO_7^+$, 382.1585; found, 382.1588.

2.3.2 (*E*)-Allyl 2-methoxy-3-(3-(methoxy(methyl)amino)-3-oxoprop-1-en-1-yl)piperidine-1-carboxylate (6a)

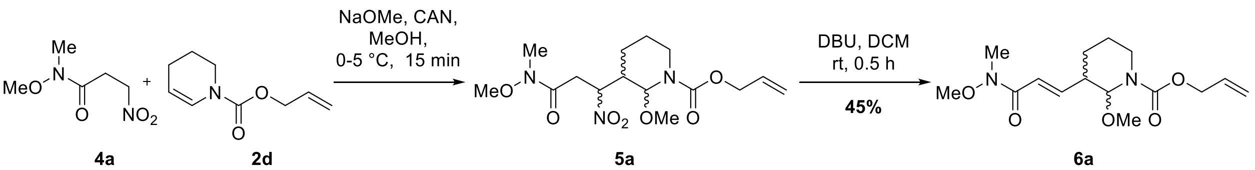

4a **2d** **5a** **6a**

Scheme S7

To a mixture of nitroalkane **4a** (0.60 mmol, 0.097 g) in MeOH (2 mL) cooled to 0-5 °C (ice-bath) was added MeONa (1.3 eq., 0.78 mmol, 0.17 g of 25% w/w solution in MeOH) immediately followed by addition of mixture of alkene **2d** (3.0 eq., 1.80 mmol, 0.301 g) in MeOH (1 mL) and solution of CAN (2.2 eq., 1.3 mmol, 0.724 g) in MeOH (3 mL). The resulting mixture was vigorously stirred (500 rpm)

at 0-5 °C. After 15 minutes, a saturated aqueous solution of $Na_2S_2O_3$ (10 mL) and water (10 mL) were added, and the mixture was extracted with EtOAc (2 × 10 mL). The combined organic phases were washed with brine (10 mL), dried (Na_2SO_4), and concentrated *in vacuo*. This residue containing **5a** was used in the following elimination phase.

The residue containing **5a** (0.600 mmol, theoretical amount from the previous step) was dissolved in DCM (6 mL), and DBU (1.5 eq., 0.90 mmol, 0.13 mL) was added at room temperature. The resulting mixture was stirred at rt. After 15 minutes, the mixture was concentrated *in vacuo*. The crude mixture was purified by column chromatography (gradient eluent hexane:EtOAc 90:10 to 85:15 to 75:25 to 50:50), yielding the amide **6a** (0.084 g, 45%) as a yellow oil.

^{1}H NMR ($CDCl_3$) and **^{13}C{^{1}H} NMR** ($CDCl_3$): Due to the complexity of compound **6a** containing multiple diastereomers, the individual signals are not listed. However, the recorded NMR spectra are available in Section 4 on p. S38. **HRMS** (*m/z*): The molecular ion was not observed; however, an elimination product (the loss of MeOH) was detected. $[M\text{-}MeOH+H]^+$ calcd for $C_{14}H_{21}N_2O_4^+$, 281.1496; found, 281.1498.

2.4 Synthesis and characterization of the denitrative cross-coupling products

2.4.1 General procedure A

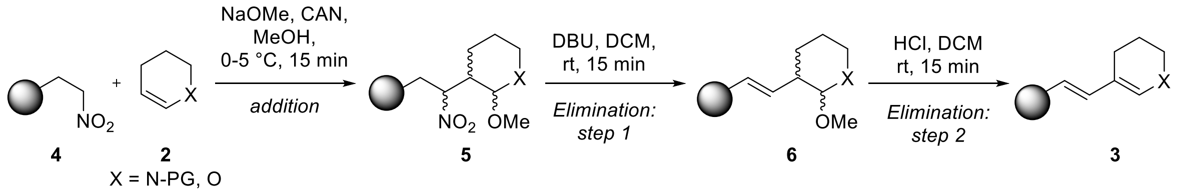

Scheme S8

Addition Phase

To a mixture of nitroalkane **4** (1 eq., 0.60 mmol) in MeOH (2 mL) cooled to 0-5 °C (ice-bath) was added MeONa (1.3 eq., 0.78 mmol, 0.17 g, of 25% w/w solution in MeOH) immediately followed by addition of solution of alkene **2** (3.0 eq., 1.8 mmol) in MeOH (1 mL) and solution of CAN (2.2 eq., 1.3 mmol) in MeOH (3 mL). The resulting mixture (0.1 M concentration of nitroalkane **4**) was vigorously stirred (500 rpm) at 0-5 °C. After 15 minutes, a saturated aqueous solution of $Na_2S_2O_3$ (10 mL) and water (10 mL) were added, and the mixture was extracted with EtOAc (2 × 10 mL). The combined organic phases were washed with brine (10 mL), dried (Na_2SO_4), and concentrated *in vacuo*. This residue containing **5** was used in the elimination phase.

Elimination Phase

The residue containing adduct **5** (0.60 mmol) was dissolved in DCM (6 mL), and DBU (1.5 eq., 0.90 mmol, 0.13 mL) was added at room temperature. After 15 minutes, HCl (2 eq., 1.2 mmol, 0.30 mL of 4

M HCl in dioxane) was added, and the mixture was stirred at room temperature. After another 15 minutes (total elimination time was 0.5 h), water (15 mL) and EtOAc (30 mL) were added. The phases were separated, and the aqueous phase was further extracted with EtOAc (15 mL). The combined organic phases were washed with brine (15 mL), dried with Na_2SO_4, and concentrated *in vacuo*. The crude mixture was purified by column chromatography, yielding product **3**.

2.4.2 Allyl (*E*)-5-(3-(methoxy(methyl)amino)-3-oxoprop-1-en-1-yl)-3,4-dihydropyridine-1(2*H*)-carboxylate (3d)

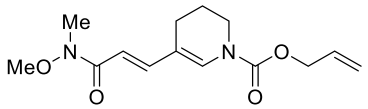

3d

Figure S3

Addition

Carbamate **3d** was prepared according to the general procedure A using a solution of nitroalkane **4a** (0.60 mmol, 97 mg) in MeOH (2 mL), MeONa (1.3 eq., 0.78 mmol, 0.17 g, 0.18 mL of 25% w/w solution of MeONa in MeOH), a solution of enecarbamate **2d** (3.0 eq., 1.80 mmol, 301 mg) in MeOH (1 mL) and a solution of CAN (2.2 eq., 1.32 mmol, 724 mg) in MeOH (3 mL).

Elimination

Elimination proceeded in DCM (6 mL) using DBU (1.5 eq., 0.90 mmol, 0.14 g, 0.13 mL) for step 1 and HCl (3.0 eq., 1.80 mmol, 0.450 mL of 4 M HCl in dioxane) for step 2. The crude product was purified by column chromatography (gradient eluent hexane:EtOAc 90:10 to 75:25 to 50:50), affording the product **3d** (72 mg, 43%) as a pale-yellow liquid.

*NMR spectra of compound **3d** show a rotameric mixture and are reported as observed*

^{1}H NMR (300 MHz, $CDCl_3$) δ 7.42 – 7.25 (m, 2H), 6.30 (d, *J* = 14.6 Hz, 1H), 5.96 (ddt, *J* = 17.2, 10.4, 5.7 Hz, 1H), 5.35 (dq, *J* = 17.2, 1.5 Hz, 1H), 5.28 (dq, *J* = 10.5, 1.3 Hz, 1H), 4.69 (dt, *J* = 5.7, 1.4 Hz, 2H), 3.71 (s, 3H), 3.75 – 3.61 (m, 2H), 3.26 (s, 3H), 2.26 (t, *J* = 6.3 Hz, 2H), 1.92 (q, *J* = 6.2 Hz, 2H). **^{13}C{^{1}H} NMR** (151 MHz, $CDCl_3$) δ 168.0, 153.3, 152.8, 144.8, 133.0, 132.5, 132.2, 118.6, 116.2, 110.6, 67.2, 61.7, 42.5, 32.7, 21.4, 20.9. **HRMS** (*m/z*): $[M+H]^+$ calcd for $C_{14}H_{21}N_2O_4^+$, 281.1496; found, 281.1498.

2.4.2.1 Synthesis of compound 3d on 3 mmol scale

Addition

Carbamate **3d** was prepared according to the general procedure A using a solution of nitroalkane **4a** (3.00 mmol, 0.486 g) in MeOH (10 mL), MeONa (1.3 eq., 3.90 mmol, 0.843 g of 25% w/w solution of

MeONa in MeOH), a solution of enecarbamate **2d** (3.0 eq., 9.00 mmol, 1.50 g) in MeOH (5 mL) and a solution of CAN (2.2 eq., 6.60 mmol, 3.62 g) in MeOH (15 mL).

Elimination

Elimination proceeded in DCM (30 mL) using DBU (1.5 eq., 4.5 mmol, 0.69 g, 0.67 mL) for step 1 and HCl (2.0 eq., 0.60 mmol, 1.5 mL of 4 M HCl in dioxane) for step 2. The crude product was purified by column chromatography (gradient eluent hexane:EtOAc 90:10 to 75:25 to 50:50), affording product **3d** (0.395 g, 47%) as a pale-orange oil.

2.4.3 Benzyl (*E*)-5-(3-(methoxy(methyl)amino)-3-oxoprop-1-en-1-yl)-3,4-dihydropyridine-1(2*H*)-carboxylate (3e)

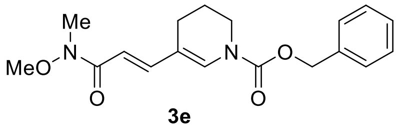

3e

Figure S4

Addition

Carbamate **3e** was prepared according to the general procedure A using a mixture of nitroalkane **4a** (0.60 mmol, 97 mg) in MeOH (1 mL), MeONa (1.3 eq., 0.78 mmol, 0.17 g, 0.18 mL of 25% w/w solution of MeONa in MeOH), mixture of enecarbamate **2e** (3.0 eq., 1.80 mmol, 391 mg) in MeOH (3 mL) and solution of CAN (2.2 eq., 1.32 mmol, 724 mg) in MeOH (2 mL).

Elimination

Elimination proceeded in DCM (6 mL) using DBU (1.5 eq., 0.900 mmol, 0.137 g, 0.134 mL) for step 1 and HCl (2.0 eq., 1.20 mmol, 0.044 g, 0.300 mL of 4 M HCl in dioxane) for step 2. The crude product was purified by column chromatography (gradient eluent hexane:EtOAc 90:10 to 85:15 to 50:50) affording carbamate **3e** (114 mg, 58%) as a yellow oil.

NMR data for the rotameric mixture are reported as observed

^{1}H NMR (300 MHz, $CDCl_3$) δ 7.42 – 7.27 (m, 8H), 6.29 (dd, J = 15.3, 0.7 Hz, 1H), 5.23 (s, 2H), 3.71 (s, 3H), 3.69 – 3.63 (m, 2H), 3.26 (s, 3H), 2.25 (t, J = 6.3 Hz, 2H), 1.97 – 1.85 (m, 2H). **^{13}C{^{1}H} NMR** (75 MHz, $CDCl_3$) δ 168.0, 155.5, 144.8, 135.8, 132.6, 128.8, 128.6, 128.3, 116.3, 110.6, 68.3, 61.7, 42.5, 32.7, 21.4, 20.9. **HRMS** (*m/z*): $[M+H]^+$ calcd for $C_{18}H_{23}N_2O_4^+$, 331.1652; found, 331.1657.

2.4.4 *tert*-Butyl (*E*)-5-(3-(methoxy(methyl)amino)-3-oxoprop-1-en-1-yl)-3,4-dihydropyridine-1(2*H*)-carboxylate (3f)

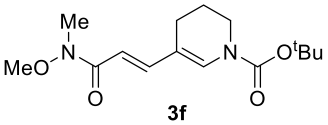

3f

Figure S5

Addition

Carbamate **3f** was prepared according to the general procedure A using a solution of nitroalkane **4a** (0.60 mmol, 97 mg) in MeOH (1 mL), MeONa (1.3 eq., 0.78 mmol, 0.17 g of 25% w/w solution of MeONa in MeOH), solution of enecarbamate **2f** (3.0 eq., 1.80 mmol, 330 mg) in MeOH (3 mL) and solution of CAN (2.2 eq., 1.32 mmol, 724 mg) in MeOH (2 mL).

Elimination

Elimination proceeded in DCM (6 mL) using DBU (1.5 eq., 0.900 mmol, 0.137 g, 0.134 mL) for step 1 and at 0-5 °C (ice-bath) HCl (2.0 eq., 1.20 mmol, 0.300 mL of 4 M HCl in dioxane) for step 2. The crude product was purified by column chromatography (gradient eluent hexane:EtOAc 90:10 to 75:25 to 50:50), affording product **3f** (93 mg, 52%) as a yellow oil.

^{1}H NMR (600 MHz, $CDCl_3$) δ 7.43 – 7.31 (m, 1H), 7.22 (bs, 1H), 6.26 (d, J = 15.2 Hz, 1H), 3.71 (s, 3H), 3.62 – 3.54 (m, 2H), 3.26 (s, 3H), 2.24 (t, J = 6.3 Hz, 2H), 1.90 (q, J = 6.2 Hz, 2H), 1.51 (s, 9H). **^{13}C{^{1}H} NMR** (151 MHz, $CDCl_3$) δ 168.2, 152.0, 145.2, 133.8, 115.2, 109.8, 82.1, 61.7, 41.8, 32.7, 28.4, 21.4, 21.0. **HRMS** (*m/z*): $[M+H]^+$ calcd for $C_{15}H_{25}N_2O_4^+$, 297.1809; found, 297.1814.

2.4.5 (*E*)-*N*-methoxy-*N*-methyl-3-(1-tosyl-1,4,5,6-tetrahydropyridin-3-yl)acrylamide (3g)

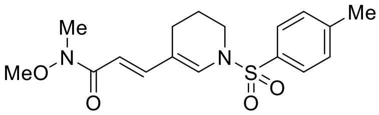

3g

Figure S6

Addition

Sulfonamide **3g** was prepared according to the general procedure A using a solution of nitroalkane **4a** (0.60 mmol, 97 mg) in MeOH (1 mL), MeONa (1.3 eq., 0.78 mmol, 0.17 g of 25% w/w solution of MeONa in MeOH), solution of enamide **2g** (3.0 eq., 1.80 mmol, 427 mg) in MeOH (3 mL) and solution of CAN (2.2 eq., 1.32 mmol, 724 mg) in MeOH (2 mL).

Elimination

Elimination proceeded in DCM (6 mL) using DBU (1.5 eq., 0.900 mmol, 0.137 g, 0.134 mL) for step 1 and HCl (2.0 eq., 1.20 mmol, 0.300 mL of 4 M HCl in dioxane) for step 2. The crude product was purified by column chromatography (gradient eluent hexane:EtOAc 90:10 to 50:50), affording product **3g** (107 mg, 51%) as a yellow oil.

^{1}**H NMR** (300 MHz, $CDCl_3$) δ 7.69 – 7.64 (m, 2H), 7.38 – 7.28 (m, 4H), 7.14 (bs, 1H), 6.24 (d, *J* = 14.7 Hz, 1H), 3.68 (s, 3H), 3.44 – 3.37 (m, 2H), 3.25 (s, 3H), 2.43 (s, 3H), 2.14 (t, *J* = 6.3 Hz, 2H), 1.88 – 1.76 (m, 2H). ^{13}**C{^{1}H} NMR** (151 MHz, $CDCl_3$) δ 167.7, 144.2, 144.1, 134.7, 132.1, 130.0, 127.0, 117.1, 110.9, 61.6, 43.7, 32.5, 21.6, 20.9, 20.5. **HRMS** (*m/z*): $[M+H]^+$ calcd for $C_{17}H_{23}N_2O_4S^+$, 351.1373; found, 351.1377.

2.4.6. *tert*-Butyl (*E*)-4-(3-(methoxy(methyl)amino)-3-oxoprop-1-en-1-yl)-2,3-dihydro-1*H*-pyrrole-1-carboxylate (3h)

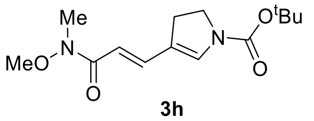

3h

Figure S7

Addition

Carbamate **3h** was prepared according to the general procedure A using a solution of nitroalkane **4a** (0.60 mmol, 97 mg) in MeOH (3 mL), MeONa (1.3 eq., 0.78 mmol, 0.17 g of 25% w/w solution of MeONa in MeOH), enecarbamate **2h** (3.0 eq., 1.80 mmol, 305 mg, 0.311 mL) and solution of CAN (2.2 eq., 1.32 mmol, 724 mg) in MeOH (3 mL).

Elimination

Elimination proceeded in DCM (6 mL) using DBU (1.5 eq., 0.900 mmol, 0.137 g, 0.134 mL) for step 1 and at 0-5 °C (ice-bath) HCl (2.0 eq., 1.20 mmol, 0.300 mL of 4 M HCl in dioxane) for step 2. The crude product was purified by column chromatography twice (gradient eluent hexane:EtOAc 90:10 to 50:50) and then (gradient eluent hexane:Et_2O 90:10 to 80:20 to 50:50), affording carbamate **3h** (86 mg, 51%) as a pale-yellow oil.

NMR spectra of compound ***3h*** *show a rotameric mixture, and are reported as observed*

^{1}**H NMR** (300 MHz, $CDCl_3$) δ 7.52 (d, *J* = 15.3 Hz, 1H), 7.03 and 6.86 (2 singlets, 1H), 6.13 (d, *J* = 15.2 Hz, 1H), 3.94 – 3.79 (m, 2H), 3.71 (s, 3H), 3.26 (s, 3H), 2.89 – 2.68 (m, 2H), 1.50 (s, 9H). ^{13}**C{^{1}H} NMR** (151 MHz, $CDCl_3$) δ 167.8, 151.1, 137.6, 135.9, 135.5, 120.4, 120.2, 112.7, 112.4, 81.5, 81.3, 61.7, 46.6, 46.1, 32.7, 28.4, 28.3, 27.5. **HRMS** (*m/z*): $[M+H]^+$ calcd for $C_{14}H_{23}N_2O_4^+$, 283.1652; found, 283.1657.

2.4.7 *tert*-Butyl (*E*)-6-(3-(methoxy(methyl)amino)-3-oxoprop-1-en-1-yl)-2,3,4,5-tetrahydro-1*H*-azepine-1-carboxylate (3i)

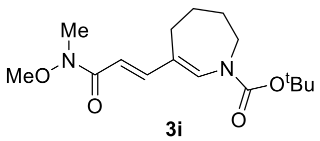

3i

Figure S8

Addition

Carbamate **3i** was prepared according to the general procedure A using a solution of nitroalkane **4a** (0.60 mmol, 97 mg) in MeOH (3 mL), MeONa (1.3 eq., 0.78 mmol, 0.17 g of 25% w/w solution of MeONa in MeOH), enecarbamate **2i** (3.0 eq., 1.80 mmol, 355 mg, 0.346 mL) and solution of CAN (2.2 eq., 1.32 mmol, 724 mg) in MeOH (3 mL).

Elimination

Elimination proceeded in DCM (6 mL) using DBU (1.5 eq., 0.900 mmol, 0.137 g, 0.134 mL) for step 1 and at 0-5 °C (ice-bath) HCl (2.0 eq., 1.20 mmol, 0.300 mL of 4 M HCl in dioxane) for step 2. The crude product was purified by column chromatography twice (gradient eluent hexane:EtOAc 90:10 to 50:50) and then (gradient eluent hexane:Et_2O 90:10 to 80:20 to 50:50), affording product **3i** (71 mg, 38%) as a pale-yellow oil.

^{1}H NMR (300 MHz, $CDCl_3$) δ 7.38 (d, J = 15.3 Hz, 1H), 7.04 (bs, 1H), 6.31 (d, J = 15.4 Hz, 1H), 3.84 – 3.74 (m, 2H), 3.71 (s, 3H), 3.26 (s, 3H), 2.47 – 2.39 (m, 2H), 1.93 – 1.78 (m, 4H), 1.50 (s, 9H). **^{13}C{^{1}H} NMR** (75 MHz, $CDCl_3$) δ 168.1, 153.2, 147.2, 139.3, 122.6, 110.9, 81.8, 61.8, 46.5, 32.7, 28.4, 27.0, 25.1, 23.8. **HRMS** (*m/z*): $[M+H]^+$ calcd for $C_{16}H_{27}N_2O_4^+$, 311.1965; found, 311.1969.

2.4.8 (*E*)-3-(1-Benzyl-6-oxo-1,4,5,6-tetrahydropyridin-3-yl)-*N*-methoxy-*N*-methylacrylamide (3j)

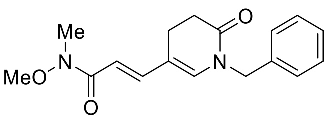

3j

Figure S9

Addition

Amide **3j** was prepared according to the general procedure A using a solution of nitroalkane **4a** (0.60 mmol, 97 mg) in MeOH (1 mL), MeONa (1.3 eq., 0.78 mmol, 0.17 g, of 25% w/w solution of MeONa in MeOH), a solution of enamide **2j** (3.0 eq., 1.80 mmol, 337 mg) in MeOH (2 mL) and solution of CAN (2.2 eq., 1.32 mmol, 724 mg) in MeOH (3 mL).

Elimination

Elimination proceeded in DCM (6 mL) using DBU (1.5 eq., 0.900 mmol, 0.137 g, 0.134 mL) for 1 h for step 1 and at 0-5 °C (ice-bath) using HCl (2.0 eq., 1.20 mmol, 0.300 mL of 4 M HCl in dioxane) for 1 h

for step 2. The crude product was purified by column chromatography twice (gradient eluent hexane:EtOAc 90:10 to 50:50 to EtOAc) and then (gradient eluent hexane:Et_2O 90:10 to 50:50 to Et_2O) affording the enamide **3j** (101 mg, 56%) as a yellow oil.

^{1}H NMR (300 MHz, $CDCl_3$) δ 7.40 – 7.18 (m, 6H), 6.48 (bs, 1H), 6.28 (d, J = 15.4 Hz, 1H), 4.73 (s, 2H), 3.70 (s, 3H), 3.25 (s, 3H), 2.73 – 2.67 (m, 2H), 2.59 – 2.53 (m, 2H). **^{13}C{^{1}H} NMR** (75 MHz, $CDCl_3$) δ 169.3, 167.6, 141.8, 136.6, 135.8, 129.0, 128.0, 128.0, 116.9, 112.3, 61.8, 49.7, 32.7, 30.8, 20.6. **HRMS** (*m/z*): $[M+H]^+$ calcd for $C_{17}H_{21}N_2O_3^+$, 301.1547; found, 301.1549.

2.4.9 (*E*)-3-(3,4-Dihydro-2*H*-pyran-5-yl)-*N*-methoxy-*N*-methylacrylamide (3k)

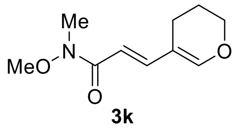

3k

Figure S10

Addition

Amide **3k** was prepared according to the general procedure A using a solution of nitroalkane **4a** (0.60 mmol, 97 mg) in MeOH (3 mL), MeONa (1.3 eq., 0.78 mmol, 0.17 g of 25% w/w solution of MeONa in MeOH), dihydropyran **2k** (20.0 eq., 12.0 mmol, 1.00 g, 1.09 mL) and solution of CAN (2.2 eq., 1.32 mmol, 724 mg) in MeOH (3 mL).

Elimination

Elimination proceeded in DCM (6 mL) using DBU (1.5 eq., 0.900 mmol, 0.137 g, 0.134 mL) for 1 hour for step 1 and HCl (3.0 eq., 1.80 mmol, 0.450 mL of 4M HCl in dioxane) for 3 hours for step 2. The crude product was purified by column chromatography (gradient eluent hexane:EtOAc 90:10 to 75:25 to 50:50), affording the product **3k** (65 mg, 55%) as a pale-yellow oil.

^{1}H NMR (400 MHz, $CDCl_3$) δ 7.31 (d, J = 15.4 Hz, 1H), 6.89 (bs, 1H), 6.22 (d, J = 15.3 Hz, 1H), 4.10 – 4.03 (m, 2H), 3.71 (s, 3H), 3.25 (s, 3H), 2.23 (t, J = 6.2 Hz, 2H), 2.00 – 1.91 (m, 2H). **^{13}C{^{1}H} NMR** (101 MHz, $CDCl_3$) δ 168.2, 152.5, 144.0, 113.1, 109.1, 66.8, 61.7, 61.6, 32.6, 21.5, 19.8. **HRMS** (m/z): $[M+H]^+$ calcd for $C_{10}H_{16}NO_3^+$, 198.1125; found, 198.1125.

2.4.10 (*E*)-*N*-Methoxy-3-(2-(methoxymethyl)-3,4-dihydro-2*H*-pyran-5-yl)-*N*-methylacrylamide (3l)

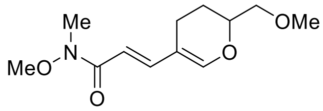

3l

Figure S11

Addition

Amide **3l** was prepared according to the general procedure A using a solution of nitroalkane **4a** (0.60 mmol, 97 mg) in MeOH (1 mL), MeONa (1.3 eq., 0.78 mmol, 0.17 g of 25% w/w solution of MeONa in MeOH), a solution of dihydropyrane **2l** (3.0 eq., 1.80 mmol, 231 mg) in MeOH (2 mL) and solution of CAN (2.2 eq., 1.32 mmol, 724 mg) in MeOH (3 mL).

Elimination

Elimination proceeded in DCM (6 mL) using DBU (1.5 eq., 0.900 mmol, 0.137 g, 0.134 mL) for 1 h for step 1 and at 0-5 °C (ice-bath) using HCl (2.0 eq., 1.20 mmol, 0.300 mL of 4 M HCl in dioxane) for 1 h for step 2. The crude product was purified by a column chromatography (gradient eluent hexane:EtOAc 90:10 to 50:50 to EtOAc) affording product **3l** (51 mg, 35%) as a pale-orange oil.

^{1}H NMR (300 MHz, $CDCl_3$) δ 7.30 (d, J = 15.3 Hz, 1H), 6.90 (bs, 1H), 6.21 (dd, J = 15.3, 0.7 Hz, 1H), 4.18 – 4.02 (m, 1H), 3.69 (s, 3H), 3.58 – 3.57 (m, 2H), 3.41 (s, 3H), 3.24 (s, 3H), 2.31 – 2.20 (m, 2H), 2.03 – 1.89 (m, 1H), 1.75 (m, 1H). **^{13}C{^{1}H} NMR** (151 MHz, $CDCl_3$) δ 168.1, 151.9, 143.6, 112.9, 109.5, 75.5, 74.6, 61.7, 59.5, 32.7, 23.3, 19.6. **HRMS** (*m/z*): $[M+H]^+$ calcd for $C_{12}H_{20}NO_4^+$, 242.1387; found, 242.1388.

2.4.11 Allyl (*E*)-5-(3-methoxy-3-oxoprop-1-en-1-yl)-3,4-dihydropyridine-1(2*H*)-carboxylate (3m)

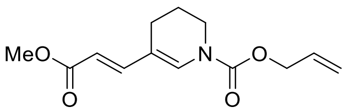

3m

Figure S12

Addition

Carbamate **3m** was prepared according to the general procedure A using a solution of nitroalkane **4b** (0.60 mmol, 80 mg) in MeOH (2 mL), MeONa (1.3 eq., 0.78 mmol, 0.17 g of 25% w/w solution of MeONa in MeOH), solution of enecarbamate **2d** (3.0 eq., 1.80 mmol, 301 mg) in MeOH (1 mL) and solution of CAN (2.2 eq., 1.32 mmol, 724 mg) in MeOH (3 mL).

Elimination

Elimination proceeded in DCM (6 mL) using DBU (1.5 eq., 0.90 mmol, 0.14 g, 0.13 mL) for step 1 and HCl (2.0 eq., 1.20 mmol, 0.300 mL of 4 M HCl in dioxane) for step 2. The crude product was purified by column chromatography (gradient eluent hexane:EtOAc 90:10 to 75:25 to 50:50), affording product **3m** (89 mg, 59%) as a pale-yellow oil.

^{1}H NMR (300 MHz, $CDCl_3$) δ 7.40 – 7.27 (m, 2H), 5.96 (ddt, *J* = 17.2, 10.4, 5.7 Hz, 1H), 5.71 (d, *J* = 15.5 Hz, 1H), 5.44 – 5.19 (m, 2H), 4.74 – 4.66 (m, 2H), 3.74 (s, 3H), 3.69 – 3.62 (m, 2H), 2.21 (t, *J* = 6.3 Hz, 2H), 1.91 (q, *J* = 6.2 Hz, 2H). **^{13}C{^{1}H} NMR** (75 MHz, $CDCl_3$) δ 168.2, 152.7, 146.2, 133.1, 132.1, 118.8, 115.7, 112.6, 67.3, 66.6, 51.5, 42.5, 21.1, 20.8. **HRMS** (*m/z*): $[M+H]^+$ calcd for $C_{13}H_{18}NO_4^+$, 252.1230; found, 252.1233.

2.4.12 *tert*-Butyl (*E*)-5-(3-methoxy-3-oxoprop-1-en-1-yl)-3,4-dihydropyridine-1(2*H*)-carboxylate (3n)

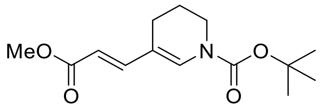

3n

Figure S13

Addition

Carbamate **3n** was prepared according to the general procedure A using a solution of nitroalkane **4b** (0.60 mmol, 80 mg) in MeOH (3 mL), MeONa (1.3 eq., 0.78 mmol, 0.17 g of 25% w/w solution of MeONa in MeOH), enecarbamate **2f** (3.0 eq., 1.80 mmol, 330 mg, 0.334 mL) and a solution of CAN (2.2 eq., 1.32 mmol, 724 mg) in MeOH (3 mL).

Elimination

Elimination proceeded in DCM (6 mL) using DBU (1.5 eq., 0.900 mmol, 0.137 g, 0.134 mL) for step 1 and at 0-5 °C (ice-bath) HCl (2.0 eq., 1.20 mmol, 0.300 mL of 4 M HCl in dioxane) for step 2. The crude product was purified by column chromatography (eluent DCM to DCM:Et_2O 80:20), affording product **3n** (64 mg, 40%) as a yellow oil.

^{1}H NMR (600 MHz, $CDCl_3$) δ 7.32 (d, *J* = 15.5 Hz, 1H), 7.20 (bs, 1H), 5.68 (d, *J* = 15.5 Hz, 1H), 3.74 (s, 3H), 3.58 (s, 2H), 2.18 (t, *J* = 6.2 Hz, 2H), 1.89 (p, *J* = 6.2 Hz, 2H), 1.51 (s, 9H). **^{13}C{^{1}H} NMR** (151 MHz, $CDCl_3$) δ 168.4, 151.9, 146.6, 134.2, 114.7, 111.8, 82.2, 51.4, 41.9, 28.3, 21.1, 20.9. **HRMS** (*m/z*): $[M+H]^+$ calcd for $C_{14}H_{22}NO_4^+$, 268.1543; found, 268.1543.

2.4.13 *tert*-Butyl (*E*)-6-(3-methoxy-3-oxoprop-1-en-1-yl)-2,3,4,5-tetrahydro-1*H*-azepine-1-carboxylate (3o)

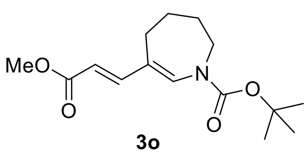

3o

Figure S14

Addition

Carbamate **3o** was prepared according to the general procedure A using a solution of nitroalkane **4b** (0.60 mmol, 80 mg) in MeOH (3 mL), MeONa (1.3 eq., 0.78 mmol, 0.17 g of 25% w/w solution of MeONa in MeOH), enamide **2i** (3.0 eq., 1.80 mmol, 355 mg, 0.346 mL) and a solution of CAN (2.2 eq., 1.32 mmol, 724 mg) in MeOH (3 mL).

Elimination

Elimination proceeded in DCM (6 mL) using DBU (1.5 eq., 0.900 mmol, 0.137 g, 0.134 mL) for step 1 and at 0-5 °C (ice-bath) HCl (2.0 eq., 1.20 mmol, 0.300 mL of 4 M HCl in dioxane) for step 2. The crude product was purified by column chromatography twice (gradient eluent hexane:EtOAc 95:5 to 75:25) and then (gradient eluent hexane to hexane:DCM 25:75 to 15:85), affording product **3o** (63 mg, 37%) as a yellow oil.

^{1}H NMR (600 MHz, $CDCl_3$) δ 7.33 (d, J = 15.5 Hz, 1H), 7.02 (bs, 1H), 5.72 (d, J = 15.5 Hz, 1H), 3.79 – 3.72 (m, 5H), 2.42 – 2.33 (m, 2H), 1.90 – 1.79 (m, 4H), 1.51 (s, 9H). **^{13}C{^{1}H} NMR** (151 MHz, $CDCl_3$) δ 168.3, 153.1, 148.6, 139.8, 122.1, 112.9, 82.0, 51.5, 31.1, 28.39, 26.9, 24.8, 23.8. **HRMS** (*m/z*): $[M+H]^+$ calcd for $C_{15}H_{24}NO_4^+$, 282.1670; found, 282.1691.

2.4.14 Methyl (*E*)-3-(1-tosyl-1,4,5,6-tetrahydropyridin-3-yl)acrylate (3p)

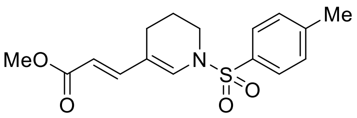

3p

Figure S15

Addition

Sulfonamide **3p** was prepared according to the general procedure A using a solution of nitroalkane **4b** (0.60 mmol, 80 mg) in MeOH (1 mL), MeONa (1.3 eq., 0.78 mmol, 0.17 g of 25% w/w solution of MeONa in MeOH), a mixture of enamide **2g** (3.0 eq., 1.80 mmol, 427 mg) and MeOH (3 mL) and a solution of CAN (2.2 eq., 1.32 mmol, 724 mg) in MeOH (2 mL).

Elimination

Elimination proceeded in DCM (6 mL) using DBU (1.5 eq., 0.900 mmol, 0.137 g, 0.134 mL) for step 1 and HCl (2.0 eq., 1.20 mmol, 0.300 mL of 4 M HCl in dioxane) for step 2. The crude product was purified by column chromatography (gradient eluent hexane:EtOAc 90:10 to 85:15 to 75:25), affording product **3p** (91 mg, 47%) as a yellow oil.

^{1}H NMR (600 MHz, $CDCl_3$) δ 7.68 – 7.65 (m, 2H), 7.35 – 7.27 (m, 3H), 7.14 (s, 1H), 5.65 (d, *J* = 15.0 Hz, 1H), 3.74 (s, 3H), 3.44 – 3.37 (m, 2H), 2.43 (s, 3H), 2.08 (t, *J* = 6.5 Hz, 2H), 1.84 – 1.77 (m, 2H). **^{13}C{^{1}H} NMR** (151 MHz, $CDCl_3$) δ 168.0, 145.6, 144.5, 134.8, 132.7, 130.2, 127.2, 116.6, 113.0, 51.5, 43.8, 21.7, 20.7, 20.6. **HRMS** (*m/z*): $[M+H]^+$ calcd for $C_{16}H_{20}NO_4S^+$, 322.1108; found, 322.1110.

2.4.15 Allyl (*E*)-5-(3-ethoxy-3-oxoprop-1-en-1-yl)-3,4-dihydropyridine-1(2*H*)-carboxylate (3q)

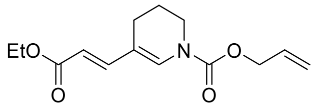

3q

Figure S16

Addition

Carbamate **3q** was prepared according to the general procedure A using a solution of nitroalkane **4c** (0.60 mmol, 88 mg) in MeOH (2 mL), MeONa (1.3 eq., 0.78 mmol, 0.17 g, 0.18 mL of 25% w/w solution of MeONa in MeOH), solution of enecarbamate **2d** (3.0 eq., 1.80 mmol, 301 mg) in MeOH (1 mL) and solution of CAN (2.2 eq., 1.32 mmol, 724 mg) in MeOH (3 mL).

Elimination

Elimination proceeded in DCM (6 mL) using DBU (1.5 eq., 0.90 mmol, 0.14 g, 0.13 mL) for step 1 and HCl (3.0 eq., 1.80 mmol, 0.450 mL of 4 M HCl in dioxane) for step 2. The crude product was purified by column chromatography (gradient eluent hexane:EtOAc 90:10 to 75:25 to 50:50), affording carbamate **3q** (68 mg, 43%) as a pale-yellow solid.

^{1}H NMR (400 MHz, $CDCl_3$) δ 7.41 – 7.22 (m, 2H), 5.96 (ddt, *J* = 17.2, 10.4, 5.7 Hz, 1H), 5.71 (d, *J* = 15.5 Hz, 1H), 5.41 – 5.25 (m, 2H), 4.69 (dt, *J* = 5.7, 1.4 Hz, 2H), 4.20 (q, *J* = 7.1 Hz, 2H), 3.70 – 3.62 (m, 2H), 2.21 (t, *J* = 6.3 Hz, 2H), 1.91 (p, *J* = 6.2 Hz, 2H), 1.30 (t, *J* = 7.1 Hz, 3H). **^{13}C{^{1}H} NMR** (101 MHz, $CDCl_3$) δ 167.8, 153.2, 145.9, 132.8, 132.2, 118.8, 115.8, 113.1, 67.3, 60.2, 42.5, 20.9, 14.5. **HRMS** (*m/z*): $[M+H]^+$ calcd for $C_{14}H_{20}NO_4^+$, 266.1387; found, 266.1389.

2.4.16 Benzyl (*E*)-3-(1-tosyl-1,4,5,6-tetrahydropyridin-3-yl)acrylate (3r)

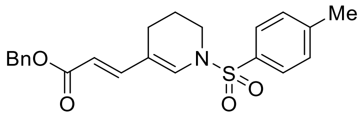

3r

Figure S17

Addition

Sulfonamide **3r** was prepared according to the general procedure A using a mixture of nitroalkane **4d** (0.600 mmol, 126 mg) in MeOH (2 mL), MeONa (1.3 eq., 0.78 mmol, 0.17 g of 25% w/w solution of MeONa in MeOH), mixture of enamide **2g** (3.0 eq., 1.80 mmol, 427 mg) and MeOH (3 mL) and solution of CAN (2.2 eq., 1.32 mmol, 724 mg) in MeOH (2 mL).

Elimination

Elimination proceeded in DCM (6 mL) using DBU (1.5 eq., 0.900 mmol, 0.137 g, 0.134 mL) for step 1 and HCl (2.0 eq., 1.20 mmol, 0.300 mL of 4 M HCl in dioxane) for step 2. The crude product was purified by column chromatography (gradient eluent toluene:EtOAc 99:1 to 95:5), affording product **3r** (101 mg, 42%) as a yellow oil.

^{1}H NMR (300 MHz, $CDCl_3$) δ 7.69 – 7.63 (m, 2H), 7.41 – 7.28 (m, 8H), 7.14 (s, 1H), 5.70 (dd, *J* = 15.5, 0.6 Hz, 1H), 5.19 (s, 2H), 3.47 – 3.33 (m, 2H), 2.43 (s, 3H), 2.07 (t, *J* = 6.3 Hz, 2H), 1.84 – 1.74 (m, 2H). **^{13}C{^{1}H} NMR** (75 MHz, $CDCl_3$) δ 167.3, 145.9, 144.5, 136.5, 134.8, 132.9, 130.2, 128.7, 128.3, 128.3, 127.1, 116.6, 113.0, 66.1, 43.8, 21.7, 20.6, 20.5. **HRMS** (*m/z*): $[M+H]^+$ calcd for $C_{22}H_{24}NO_4S^+$, 398.1421; found, 398.1426.

2.4.17 (*E*)-*N*,*N*-Dimethyl-3-(1-tosyl-1,4,5,6-tetrahydropyridin-3-yl)acrylamide (3s)

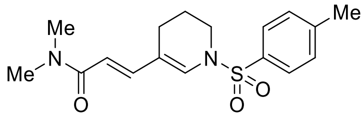

3s

Figure S18

Addition

Sulfonamide **3s** was prepared according to the general procedure A using a mixture of nitroalkane **4e** (0.60 mmol, 88 mg) in MeOH (1 mL), MeONa (1.3 eq., 0.78 mmol, 0.17 g, 0.18 mL of 25% w/w solution of MeONa in MeOH), mixture of enamide **2g** (3.0 eq., 1.80 mmol, 427 mg) and MeOH (2 mL) and solution of CAN (2.2 eq., 1.32 mmol, 724 mg) in MeOH (3 mL).

Elimination

Elimination proceeded in DCM (6 mL) using DBU (1.5 eq., 0.900 mmol, 0.137 g, 0.134 mL) for 1 h for step 1 and at 0-5 °C (ice-bath) HCl (2.0 eq., 1.20 mmol, 0.300 mL of 4 M HCl in dioxane) for 1 h for step 2. The crude product was purified by column chromatography (gradient eluent hexane:EtOAc 90:10 to 50:50 to EtOAc), affording product **3s** (86 mg, 43%) as a pale-orange solid.

^{1}H NMR (600 MHz, $CDCl_3$) δ 7.67 – 7.62 (m, 2H), 7.33 – 7.23 (m, 3H), 7.07 (bs, 1H), 6.06 (d, *J* = 14.9 Hz, 1H), 3.40 – 3.35 (m, 2H), 3.05 (bs, 3H), 3.00 (bs, 3H), 2.41 (s, 3H), 2.11 – 2.06 (m, 2H), 1.82 – 1.75 (m, 2H). **^{13}C{^{1}H} NMR** (151 MHz, $CDCl_3$) δ 167.3, 144.3, 143.1, 134.8, 131.4, 130.1, 127.1, 117.2, 112.6, 43.8, 37.4, 35.9, 21.7, 21.0, 20.6. **HRMS** (m/z): $[M+H]^+$ calcd for $C_{17}H_{23}N_2O_3S^+$, 335.1424; found, 335.1427.

2.4.18 (*E*)-Dimethyl (2-(1-tosyl-1,4,5,6-tetrahydropyridin-3-yl)vinyl)phosphonate (3t)

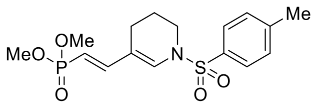

3t

Figure S19

Addition

Sulfonamide **3t** was prepared according to the general procedure A using a solution of nitroalkane **4f** (0.600 mmol, 110 mg) in MeOH (1 mL), MeONa (1.3 eq., 0.78 mmol, 0.17 g of 25% w/w solution of MeONa in MeOH), a solution of enamide **2g** (3.0 eq., 1.80 mmol, 427 mg) in MeOH (2 mL) and a solution of CAN (2.2 eq., 1.32 mmol, 724 mg) in MeOH (3 mL).

Elimination

Elimination proceeded in DCM (6 mL) using DBU (1.5 eq., 0.900 mmol, 0.137 g, 0.134 mL) for 0.5 h for step 1 and HCl (2.0 eq., 1.20 mmol, 0.300 mL of 4 M HCl in dioxane) for 4 h for step 2. The crude product was purified by column chromatography (gradient eluent hexane:EtOAc 90:10 to 50:50 to EtOAc), affording product **3t** (93 mg, 42%) as a pale-orange solid.

^{1}H NMR (600 MHz, $CDCl_3$) δ 7.69 – 7.62 (m, 2H), 7.35 – 7.29 (m, 2H), 7.13 – 7.03 (m, 2H), 5.39 – 5.30 (m, 1H), 3.72 (d, *J* = 11.0 Hz, 6H), 3.42 – 3.37 (m, 2H), 2.43 (s, 3H), 2.07 – 2.05 (m, 2H), 1.83 – 1.76 (m, 2H). **^{13}C{^{1}H} NMR** (151 MHz, $CDCl_3$) 150.0 (d, *J* = 7.4 Hz), 144.5, 134.8, 132.8, 130.2, 127.2, 116.9 (d, *J* = 25.3 Hz), 106.33 (d, *J* = 195.8 Hz), 52.46 (d, *J* = 5.6 Hz), 43.8, 21.7, 20.5, 20.4. **^{31}P NMR** (243 MHz, $CDCl_3$) δ 24.0. **HRMS** (m/z): $[M+H]^+$ calcd for $C_{16}H_{23}NO_5PS^+$, 372.1029; found, 372.1030.

2.4.19 (*E*)-5-(2-(tert-Butylsulfonyl)vinyl)-1-tosyl-1,2,3,4-tetrahydropyridine (3u)

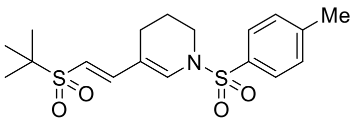

3u

Figure S20

Addition

To a mixture of nitroalkane **4g** (0.600 mmol, 117 mg), MeOH (6 mL), and MeCN (6 mL) cooled to -30 °C, MeONa (1.3 eq., 0.78 mmol, 0.17 g of 25% w/w solution of MeONa in MeOH) was added dropwise. A solution of enamide **2g** (3.0 eq., 1.80 mmol, 427 mg) in MeCN (3 mL) was added next, followed by the addition of a solution of CAN (2.2 eq., 1.32 mmol, 724 mg) in MeOH (3 mL), and the mixture was stirred vigorously at -30 °C. After 15 minutes, the mixture was warmed to 0-5 °C (ice-bath), and a saturated aqueous solution of $Na_2S_2O_3$ (10 mL) and water (10 mL) were added, and the mixture was extracted with EtOAc (2 × 15 mL). The combined organic phases were washed with brine (10 mL), dried with Na_2SO_4, and concentrated *in vacuo*. This residue was used in the elimination phase.

Elimination

Elimination proceeded in DCM (6 mL) using DBU (1.5 eq., 0.900 mmol, 0.137 g, 0.134 mL) for step 1 and at 0-5 °C (ice-bath), HCl (2.0 eq., 1.20 mmol, 0.300 mL of 4 M HCl in dioxane) for step 2. The crude product was purified by column chromatography (eluent hexane:EtOAc 90:10 to 50:50 to EtOAc), affording sulfonamide **3u** (122 mg, 53%) as a yellow oil.

^{1}H NMR (300 MHz, $CDCl_3$) δ 7.72 – 7.64 (m, 2H), 7.40 – 7.30 (m, 2H), 7.23 (s, 1H), 7.14 (dd, *J* = 15.0, 0.6 Hz, 1H), 5.96 (dd, *J* = 15.0, 0.6 Hz, 1H), 3.49 – 3.37 (m, 2H), 2.44 (s, 3H), 2.08 (t, *J* = 6.3 Hz, 2H), 1.92 – 1.78 (m, 2H), 1.36 (s, 9H). **^{13}C{^{1}H} NMR** (75 MHz, $CDCl_3$) δ 147.2, 144.8, 134.8, 134.6, 130.3, 127.2, 114.7, 114.4, 58.9, 43.6, 23.6, 21.8, 20.8, 20.4. **HRMS** (*m/z*): $[M+H]^+$ calcd for $C_{18}H_{26}NO_4S_2^+$, 384.1298; found, 384.1299.

2.4.20 (*E*)-5-Styryl-3,4-dihydro-2*H*-pyran (3v)

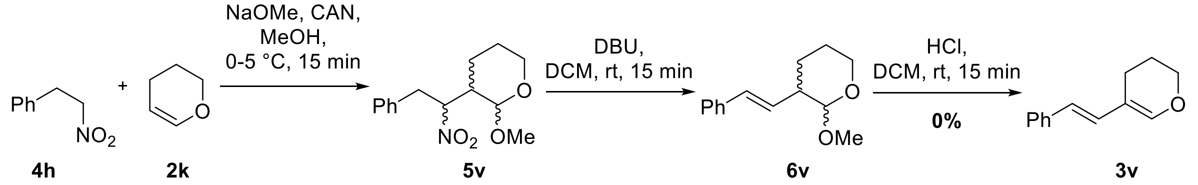

Scheme S9

Addition

To a solution of nitroalkane **4h** (0.60 mmol, 0.091 g) in MeOH (2 mL) cooled to 0-5 °C (ice-bath) was added MeONa (1.2 eq., 0.78 mmol, 0.17 g of 25% w/w solution in MeOH) immediately followed by the addition of a solution of dihydropyran (**2k**) (3.0 eq., 1.80 mmol, 0.151 g) in MeOH (1 mL) and a solution of CAN (2.2 eq., 1.32 mmol, 0.724 g) in MeOH (3 mL). The resulting mixture was vigorously stirred (500 rpm) at 0-5 °C (ice-bath). After 15 minutes, a saturated aqueous solution of $Na_2S_2O_3$ (10 mL) and water (10 mL) were added, and the mixture was extracted with EtOAc (2 × 10 mL). The combined organic phases were washed with brine (10 mL), dried (Na_2SO_4), and concentrated *in vacuo*. This residue containing adduct **5v** was used in the elimination phase.

Elimination

Nitro compound **5v** (0.6 mmol, theoretical amount from the addition) was dissolved in DCM (6 mL), and DBU (1.5 eq., 0.90 mmol, 0.13 mL) was added at room temperature. After 15 minutes, HCl (2 eq., 1.2 mmol, 0.30 mL of 4 M HCl in dioxane) was added, and the mixture was stirred at room temperature. After another 15 minutes (total elimination time was 0.5 h), water (15 mL) and EtOAc (30 mL) were added. Phases were separated, and the aqueous phase was further extracted with EtOAc (15 mL). Combined organic phases were washed with brine (15 mL), dried with Na_2SO_4, and concentrated *in vacuo*. According to the ^{1}H NMR analysis, the crude mixture contained neither **3v** nor **6v**, but did contain unreacted **5v**.

2.4.20.1 2-Methoxy-3-(1-nitro-2-phenylethyl)tetrahydro-2*H*-pyran (5v)

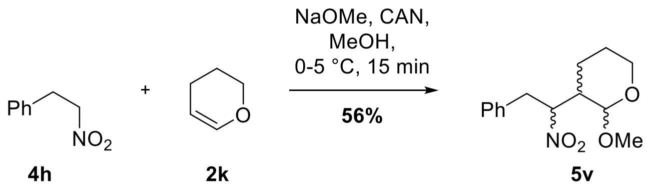

Scheme S10

Addition

To a solution of nitroalkane **4h** (0.60 mmol, 0.091 g) in MeOH (2 mL) cooled to 0-5 °C (ice-bath) was added MeONa (1.2 eq., 0.78 mmol, 0.17 g of 25% w/w solution in MeOH), immediately followed by the addition of a solution of dihydropyran (**2k**) (3.0 eq., 1.80 mmol, 151 mg, 163 μL) in MeOH (1 mL) and a solution of CAN (2.2 eq., 1.32 mmol, 0.724 g) in MeOH (3 mL). The resulting mixture was vigorously stirred (500 rpm) at 0-5 °C (ice-bath). After 15 minutes, a saturated aqueous solution of $Na_2S_2O_3$ (10 mL) and water (10 mL) were added, and the mixture was extracted with EtOAc (2 × 10 mL). The combined organic phases were washed with brine (10 mL), dried (Na_2SO_4), and concentrated *in vacuo*. This residue was purified by column chromatography (eluent hexane to hexane:EtOAc 95:5 to 90:10, yielding the adduct **5v** (89 mg, 56%) as a yellow oil.

^{1}H NMR ($CDCl_3$) and **$^{13}C\{^1H\}$ NMR** ($CDCl_3$): Due to the complexity of compound **5v** containing multiple diastereomers, the individual signals are not listed. However, the recorded NMR spectra are available in the section 4 on p. S58. **HRMS** (*m/z*): $[M+H]^+$ calcd for $C_{14}H_{20}NO_4^+$, 266.1387; found, 266.1386.

2.4.20.2 (*E*)-2-Methoxy-3-styryltetrahydro-2*H*-pyran (6v)

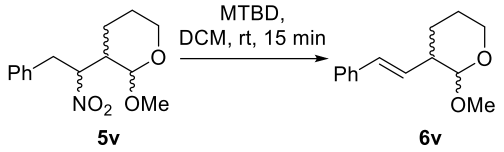

Scheme S11

Elimination

Nitro compound **5v** (0.6 mmol, theoretical amount from the addition) was dissolved in DCM (6 mL), and MTBD (1.5 eq., 0.900 mmol, 0.138 g, 0.129 mL) was added at room temperature. After 15 minutes the mixture concentrated *in vacuo*. According to the ^{1}H NMR analysis, the crude mixture did not contain **6v**, but contained unreacted **5v**.

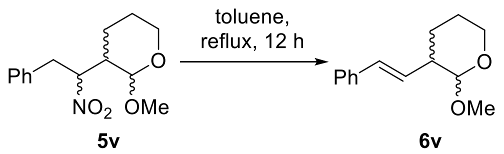

Scheme S12

Elimination

Nitro compound **5v** (0.6 mmol, theoretical amount from the addition) was dissolved in toluene (12 mL), and the mixture was stirred at reflux under argon. After 12 h, the mixture was cooled to rt and concentrated *in vacuo*. According to the ^{1}H NMR analysis, the crude mixture did not contain **6v**, but contained unreacted **5v**.

2.4.21.1 1-Methoxy-2-(1-nitro-2-phenylethyl)cyclohexane (5w)

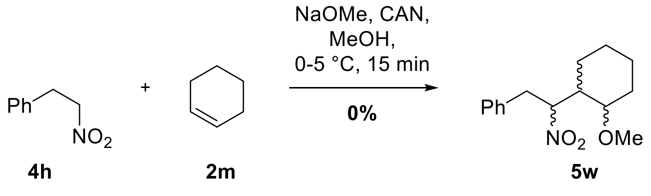

Scheme S13

Addition

To a solution of nitroalkane **4h** (0.60 mmol, 0.091 g) in MeOH (2 mL) cooled to 0-5 °C (ice-bath) was added MeONa (1.3 eq., 0.78 mmol, 0.17 g of 25% w/w solution in MeOH), immediately followed by the addition of a solution of cyclohexene (**2m**) (3.0 eq., 1.8 mmol, 0.148 g, 0.182 mL) in MeOH (1 mL) and a solution of CAN (2.2 eq., 1.32 mmol, 0.724 g) in MeOH (3 mL). The resulting mixture was vigorously stirred (500 rpm) at 0-5 °C (ice-bath). After 15 minutes, a saturated aqueous solution of $Na_2S_2O_3$ (10 mL) and water (10 mL) were added, and the mixture was extracted with EtOAc (2 × 10 mL). The combined organic phases were washed with brine (10 mL), dried (Na_2SO_4), and concentrated *in vacuo*. The ^{1}H NMR analysis indicated that **5w** was not present.

3. Mechanistic investigation

3.1 Experiment in the presence of radical scavenger TEMPO

3.1.1 2,2,6,6-Tetramethyl-1-(1-nitro-2-phenylethoxy)piperidine (16)

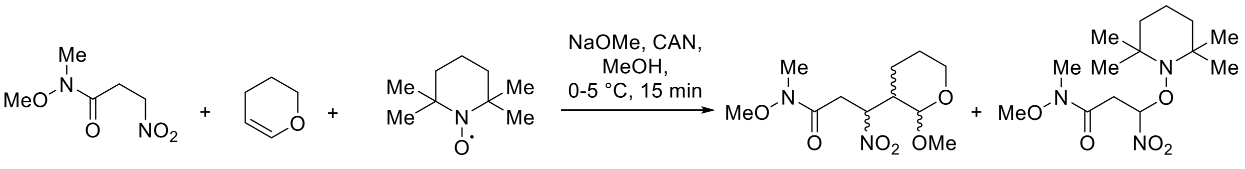

Scheme S14

To a mixture of nitroalkane **4a** (0.60 mmol, 0.097 g) in MeOH (2 mL) cooled to 0-5 °C (ice-bath) was added MeONa (1.3 eq., 0.78 mmol, 0.17 g of 25% w/w solution in MeOH) and TEMPO (**15**) (0.60 mmol, 0.094 g), immediately followed by the addition of a solution of dihydropyran (**2k**) (3.0 eq., 1.80 mmol, 0.151 g, 163 μL) in MeOH (1 mL) and a solution of CAN (2.2 eq., 1.32 mmol, 0.724 g) in MeOH (3 mL). The resulting mixture was vigorously stirred (500 rpm) at 0-5 °C. After 15 minutes, a saturated aqueous solution of $Na_2S_2O_3$ (10 mL) and water (10 mL) were added, and the mixture was extracted with EtOAc (2 × 10 mL). The combined organic phases were washed with brine (10 mL), dried (Na_2SO_4), and concentrated *in vacuo*. This residue was purified by column chromatography (gradient eluent hexane:EtOAc 90:10 to 85:15), yielding impure adduct **16** (0.152 g). This residue was purified again by column chromatography (gradient eluent hexane:DCM 50:50 to 10:90 to DCM to DCM:Et_2O 90:10), yielding the adduct **16** (0.107 g, 56%) as a yellow oil.

^{1}H NMR (300 MHz, $CDCl_3$) δ 6.03 (dd, *J* = 9.3, 3.6 Hz, 1H), 3.72 (s, 3H), 3.38 (dd, *J* = 16.7, 9.4 Hz, 1H), 3.16 (s, 3H), 3.07 (dd, *J* = 16.8, 3.6 Hz, 1H), 1.64 – 0.93 (s, 18H). **^{13}C{^{1}H} NMR** (151 MHz, $CDCl_3$) δ 167.9, 112.3, 61.8, 61.6, 60.1, 40.3, 40.1, 35.5, 33.4, 32.1, 31.6, 20.5, 20.2, 17.0. **HRMS** (*m/z*): $[M+H]^+$ calcd for $C_{14}H_{28}N_3O_5^+$, 318.2024; found, 318.2029.

3.2 HPLC analysis of the addition phase, transformation 4a to 5a

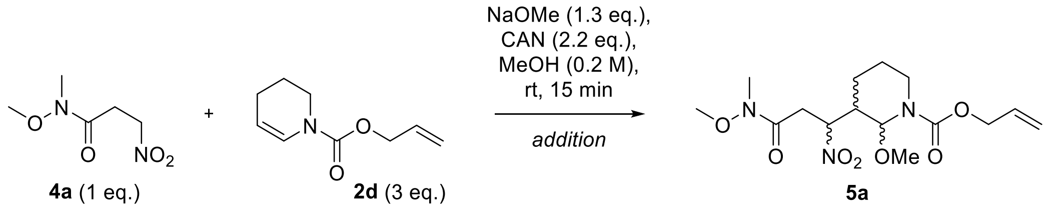

Scheme S15

The reaction was performed according to the general procedure A at rt and analyzed immediately after the addition of NaOMe. The HPLC indicated the presence of acrylamide **9**.

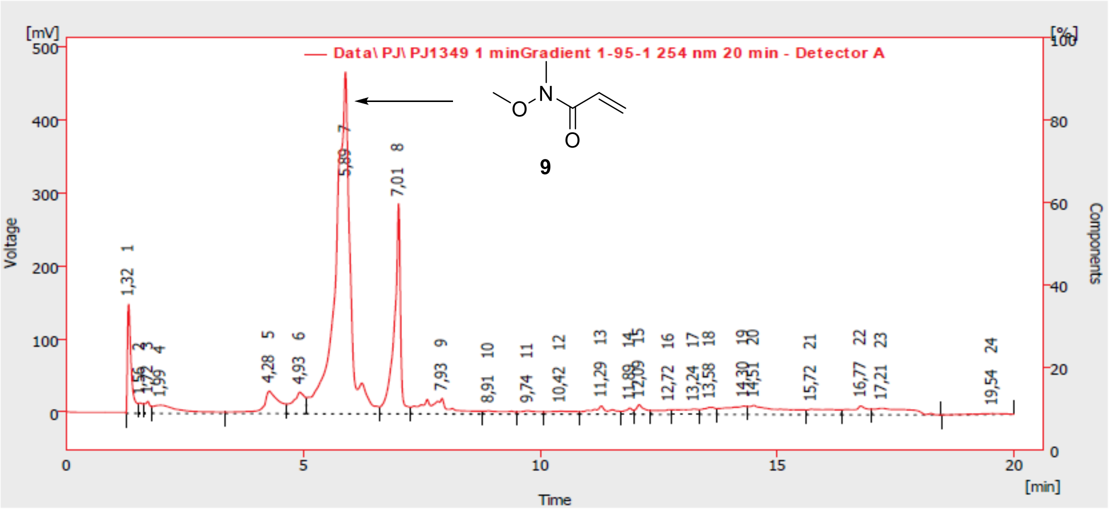

4. ^{1}H and ^{13}C{^{1}H} NMR spectra

4.1 ^{1}H and ^{13}C{^{1}H} NMR spectra of 4d

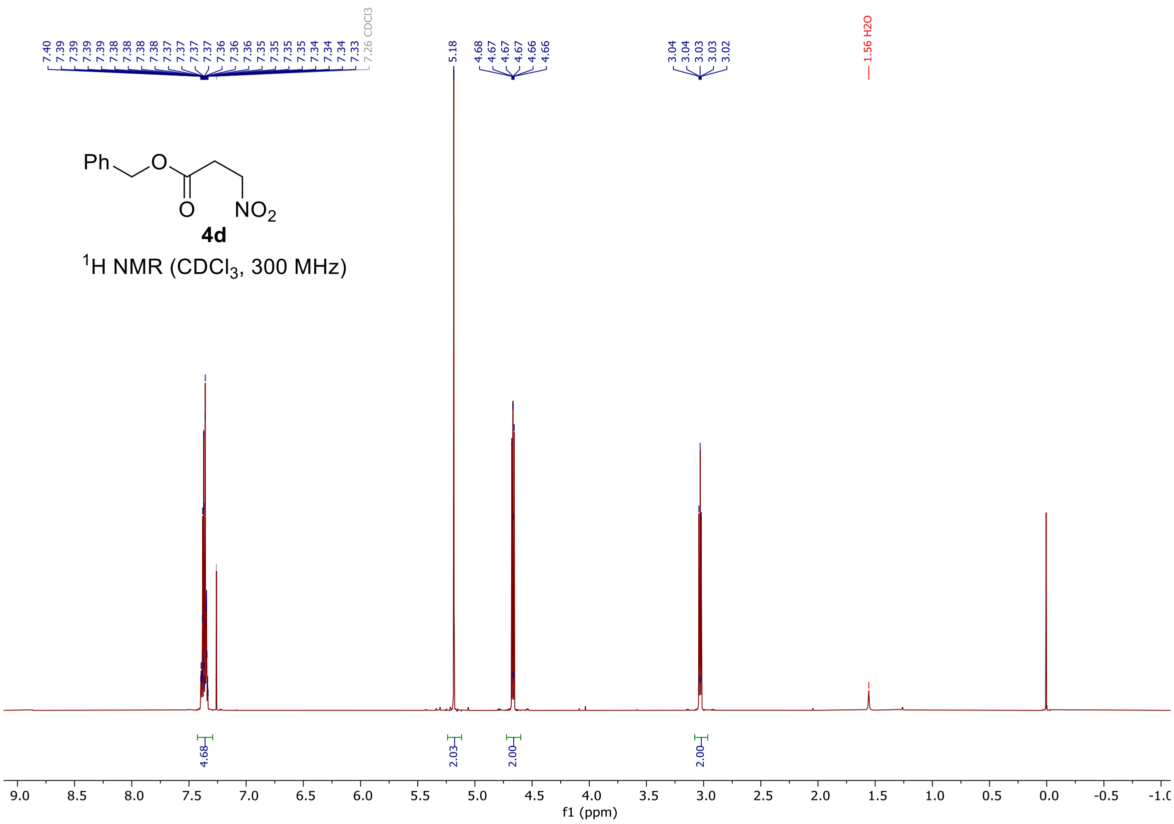

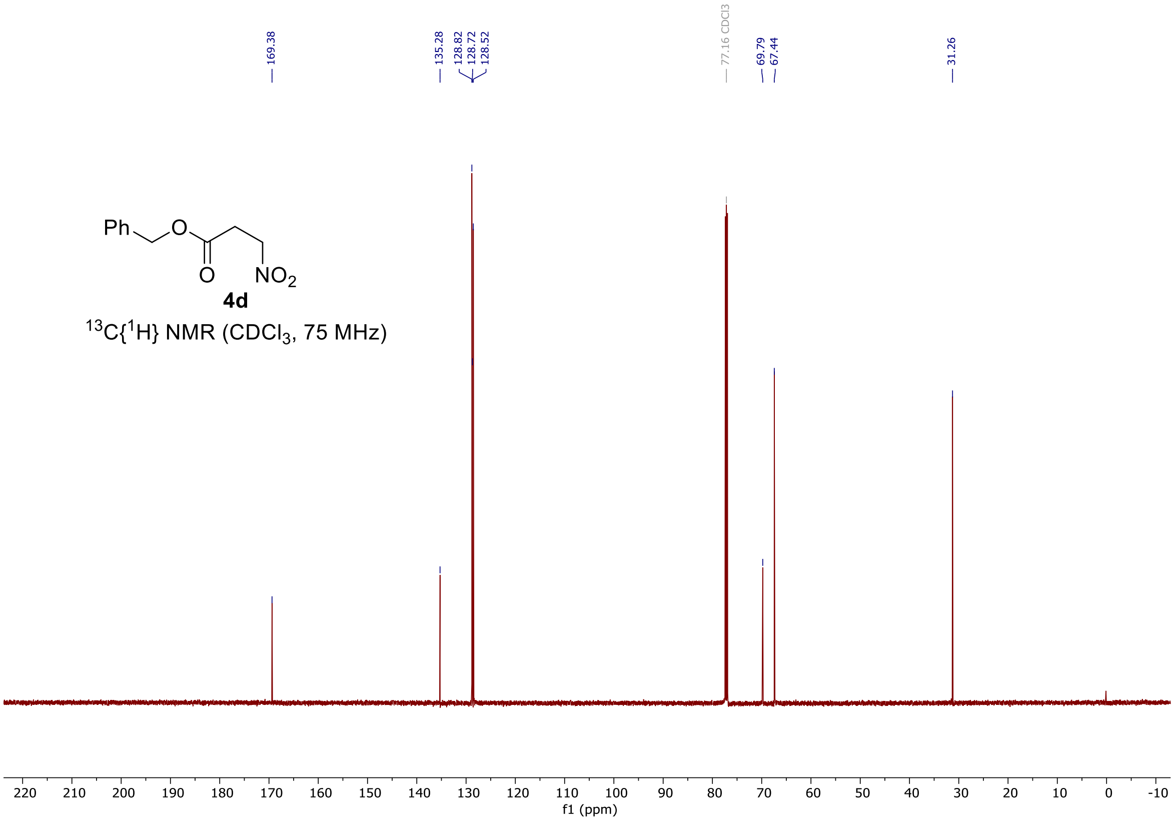

Figure S21

4.2 ^{1}H and $^{13}C\{^{1}H\}$ NMR spectra of 20

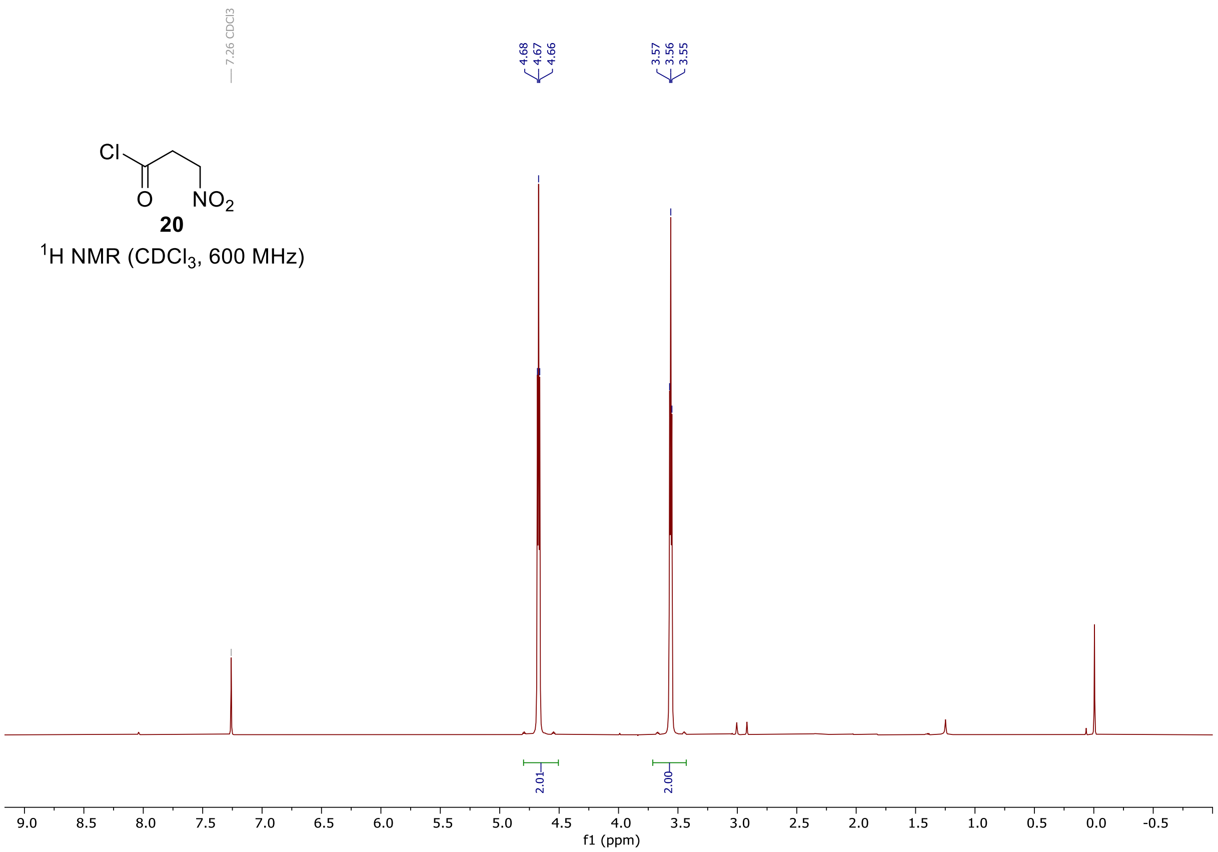

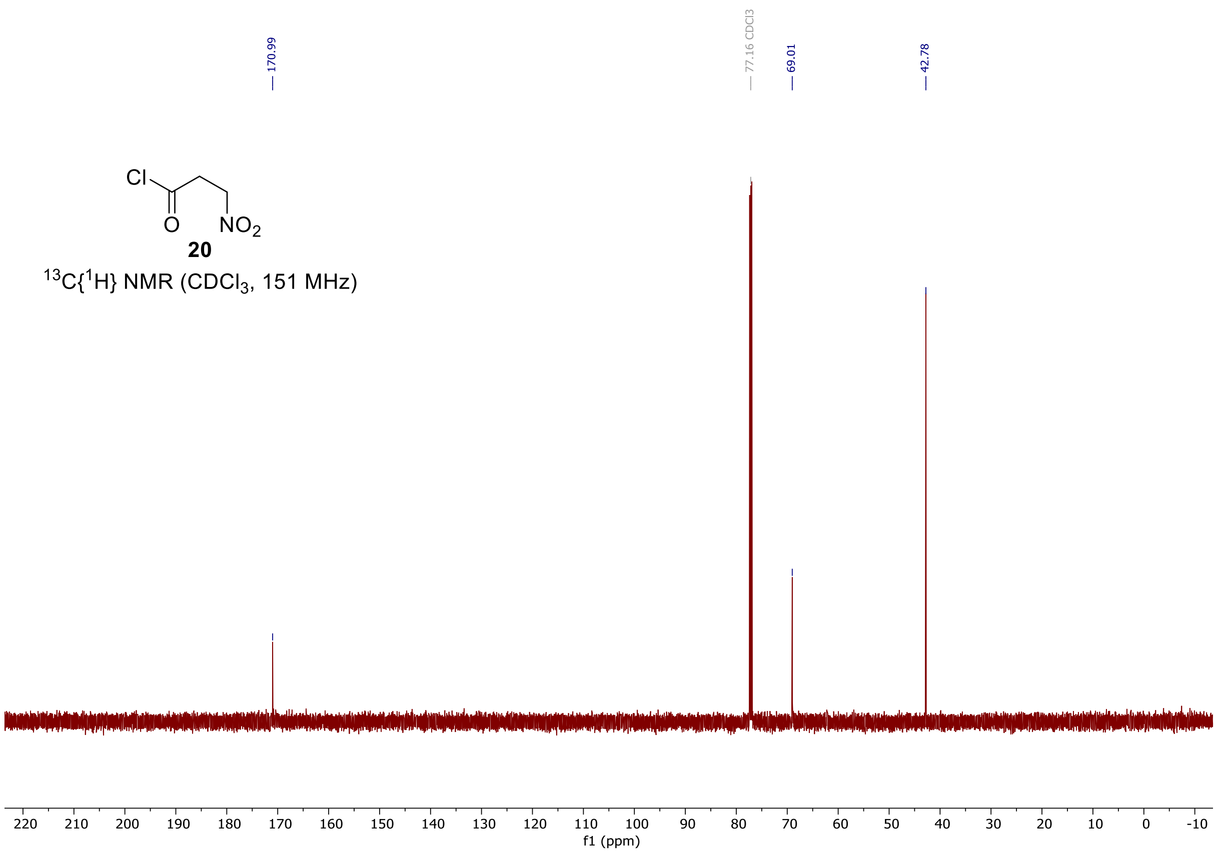

Figure S22

4.3 ^{1}H and $^{13}C\{^{1}H\}$ NMR spectra of 4e

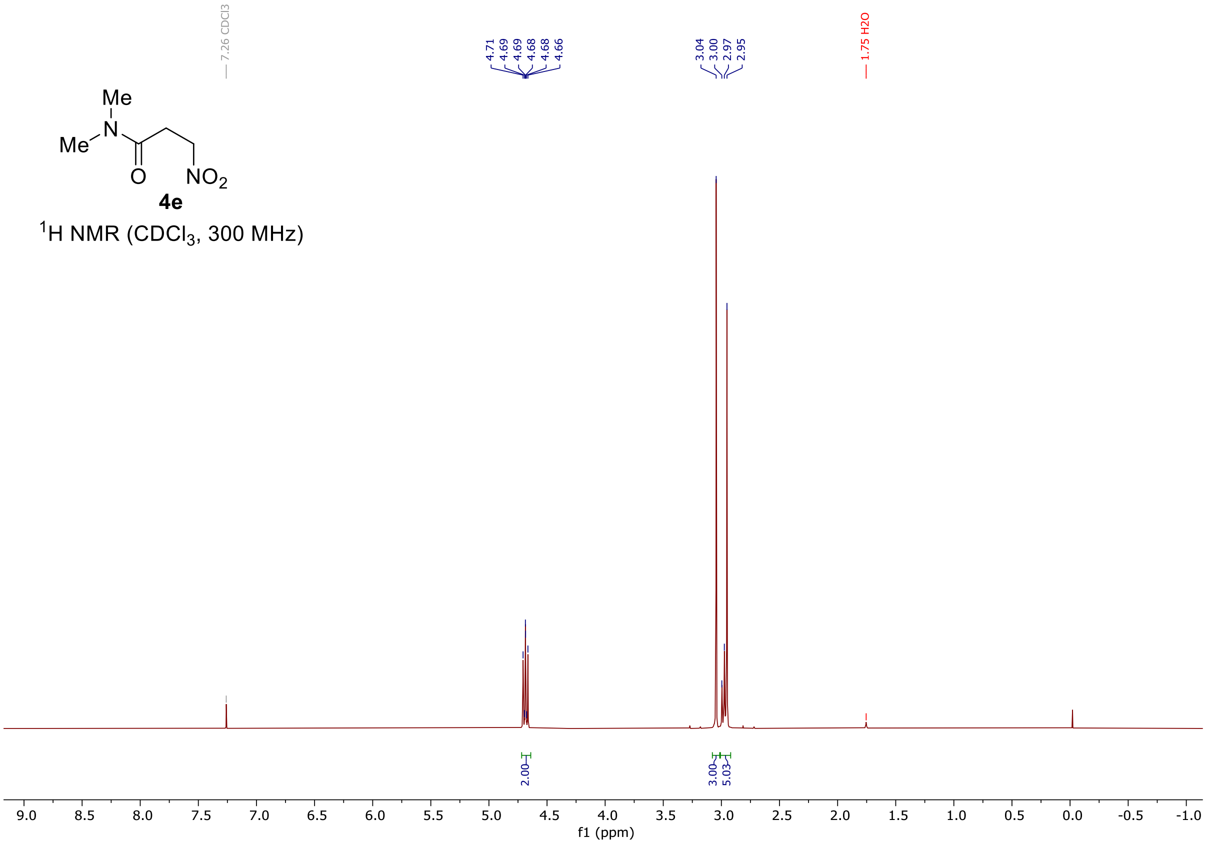

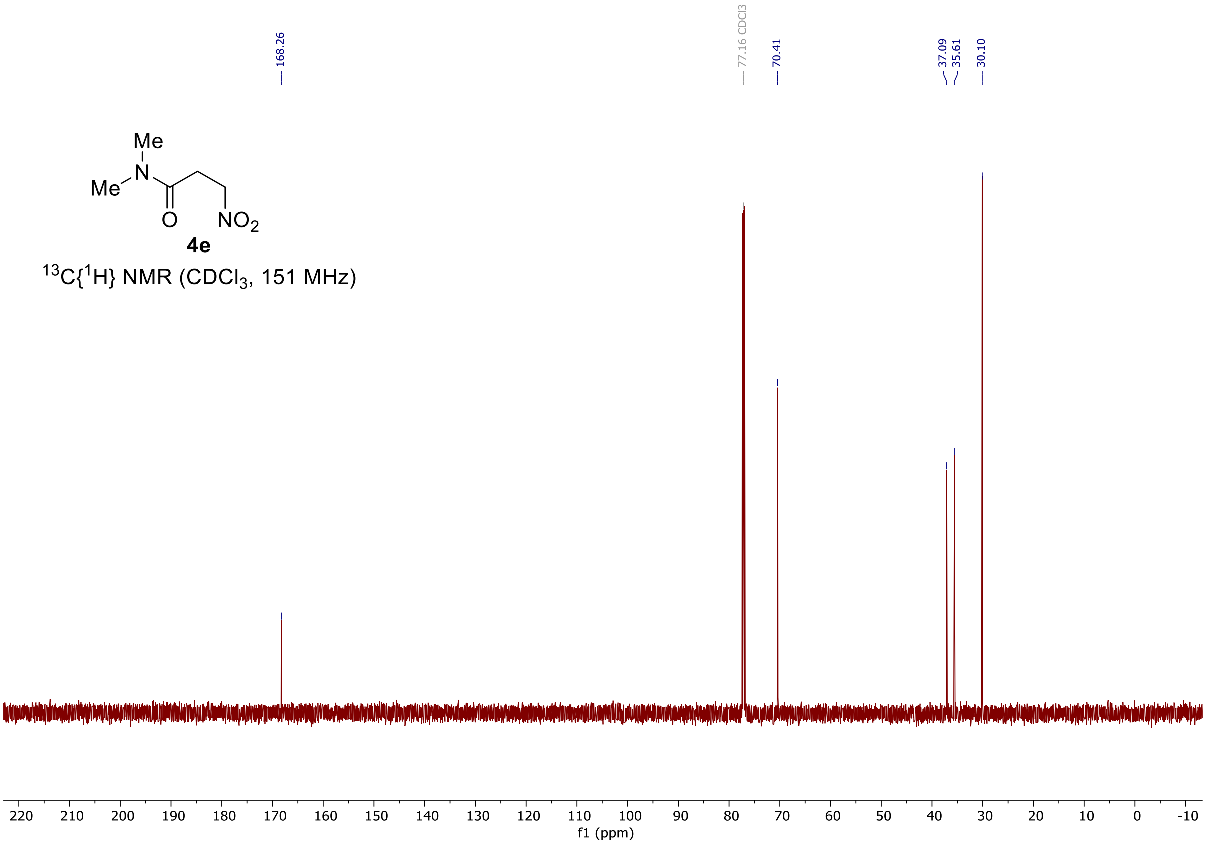

Figure S23

4.4 ^{1}H, $^{13}C\{^{1}H\}$ and ^{31}P NMR spectra of 4f

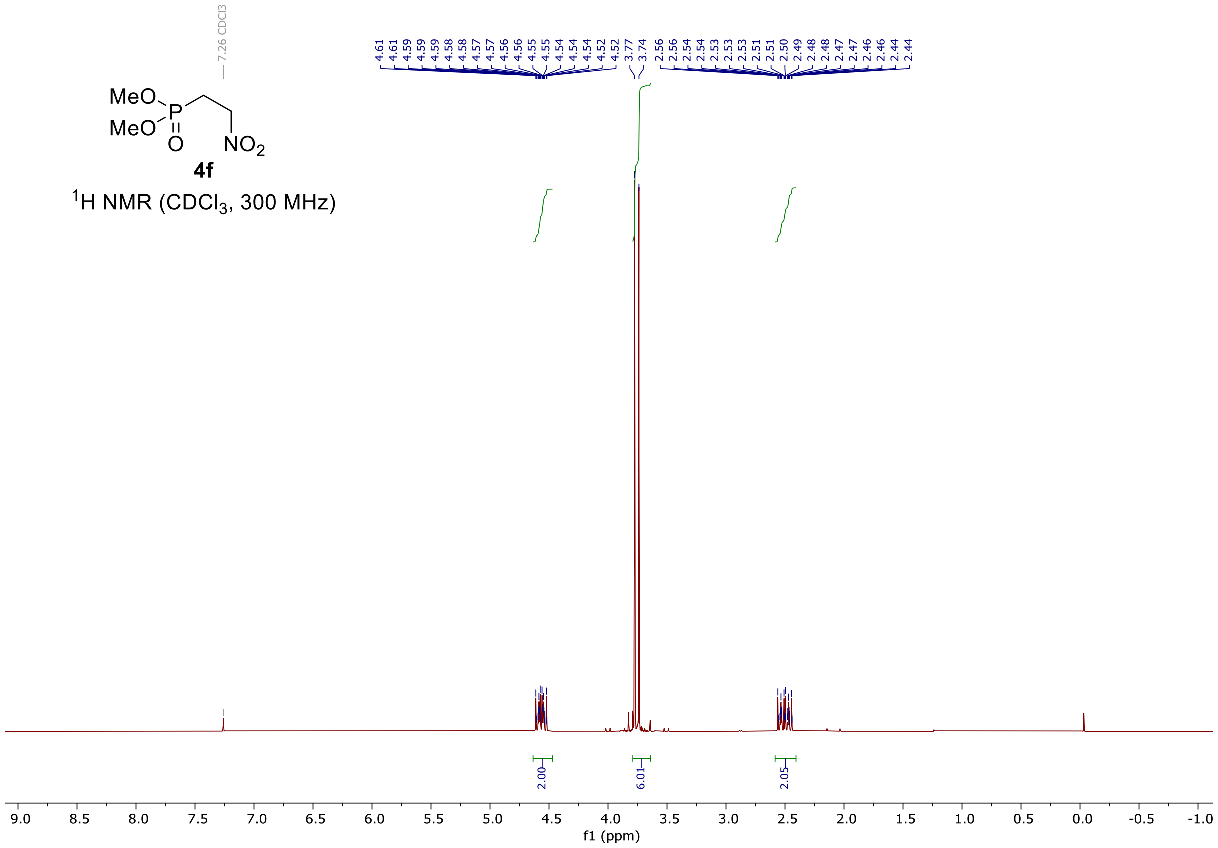

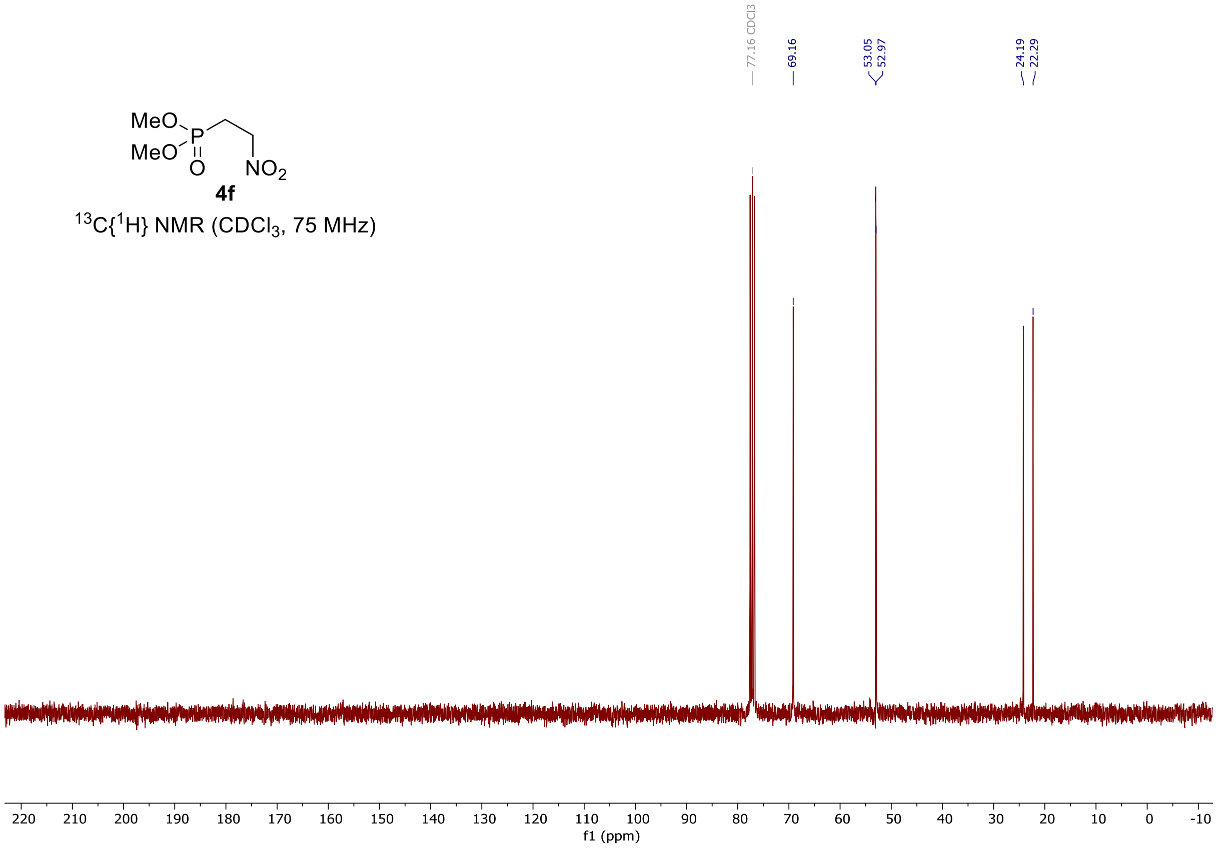

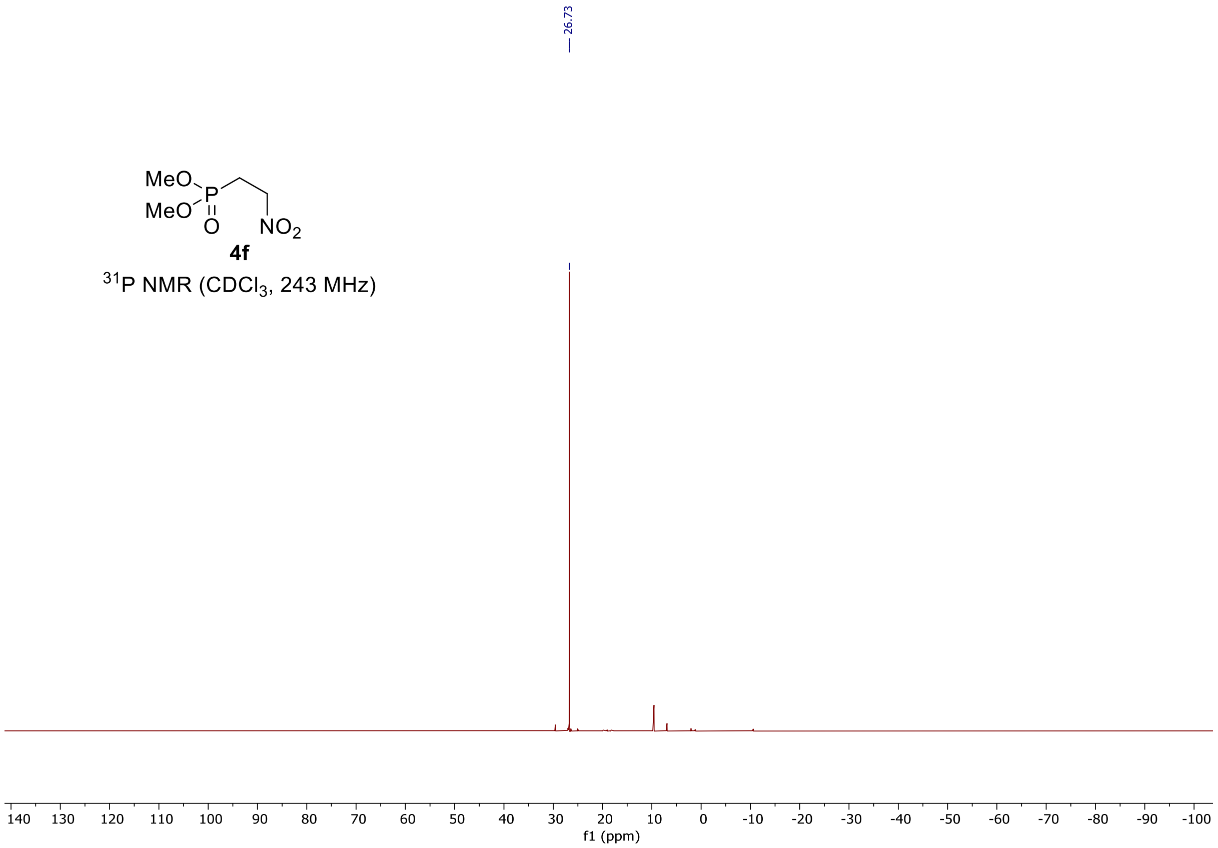
26.73
MeO
MeO
P
O
NO2
4f
31P NMR (CDCl3, 243 MHz)
140
130
120
110
100
90
80
70
60
50
40
30
20
10
0
-10
-20
-30
-40
-50
-60
-70
-80
-90
-100
f1 (ppm)

Figure S24

4.5 ^{1}H and $^{13}C\{^{1}H\}$ NMR spectra of 2d

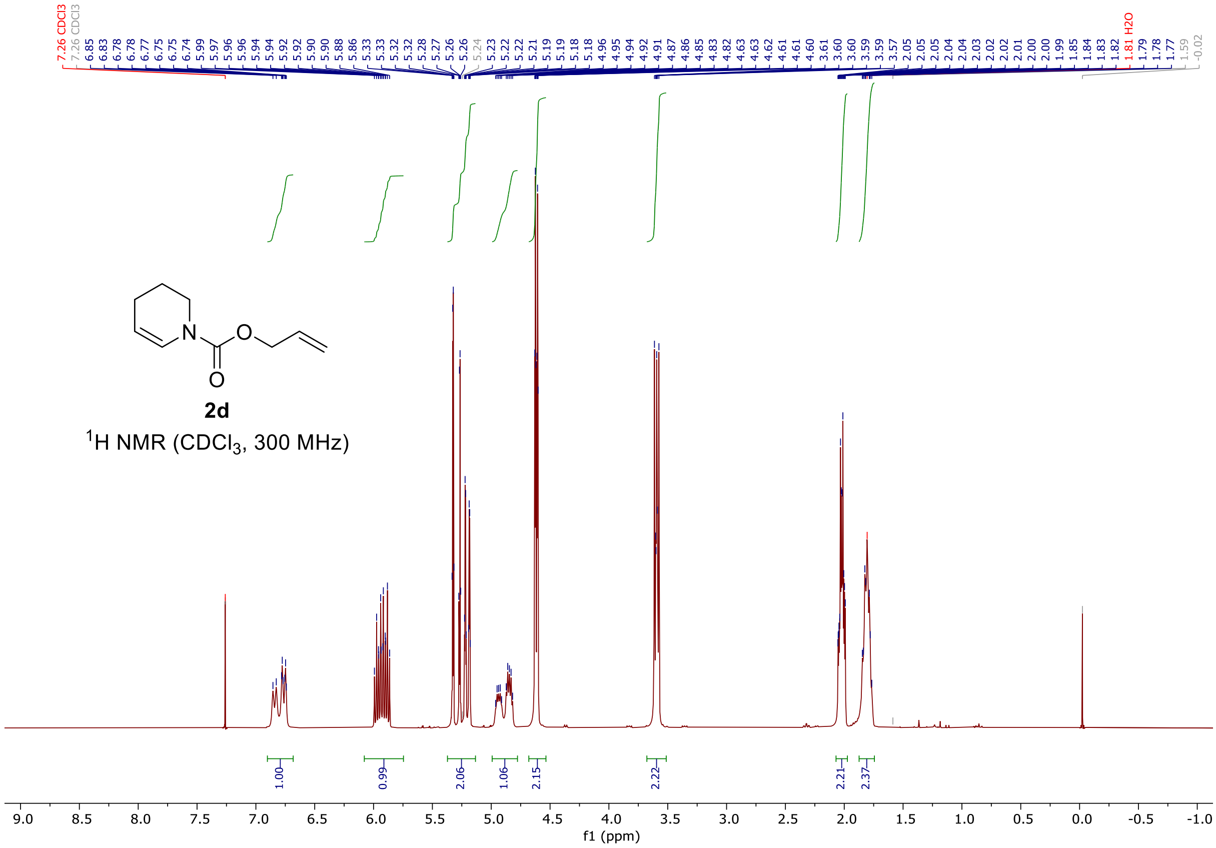

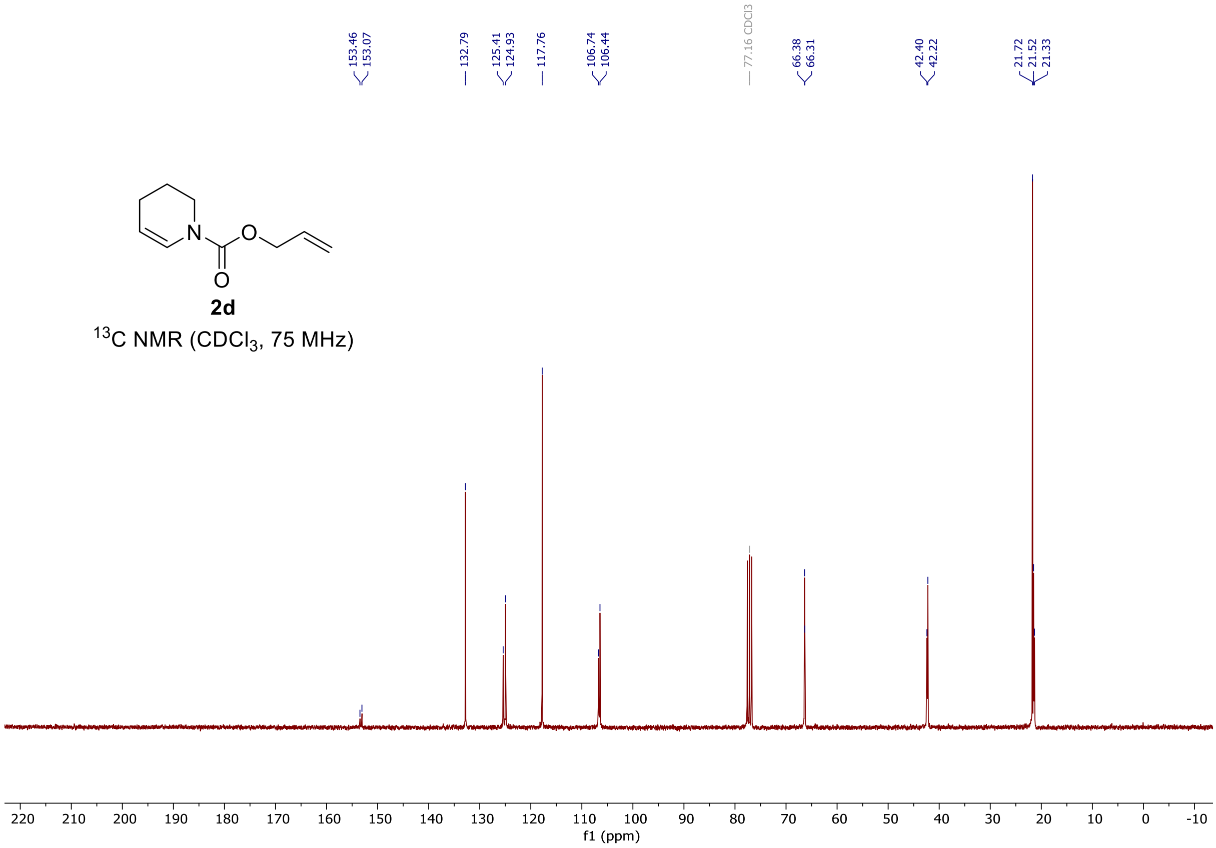

Figure S25

4.6 ^{1}H and ^{13}C{^{1}H} NMR spectra of 5a

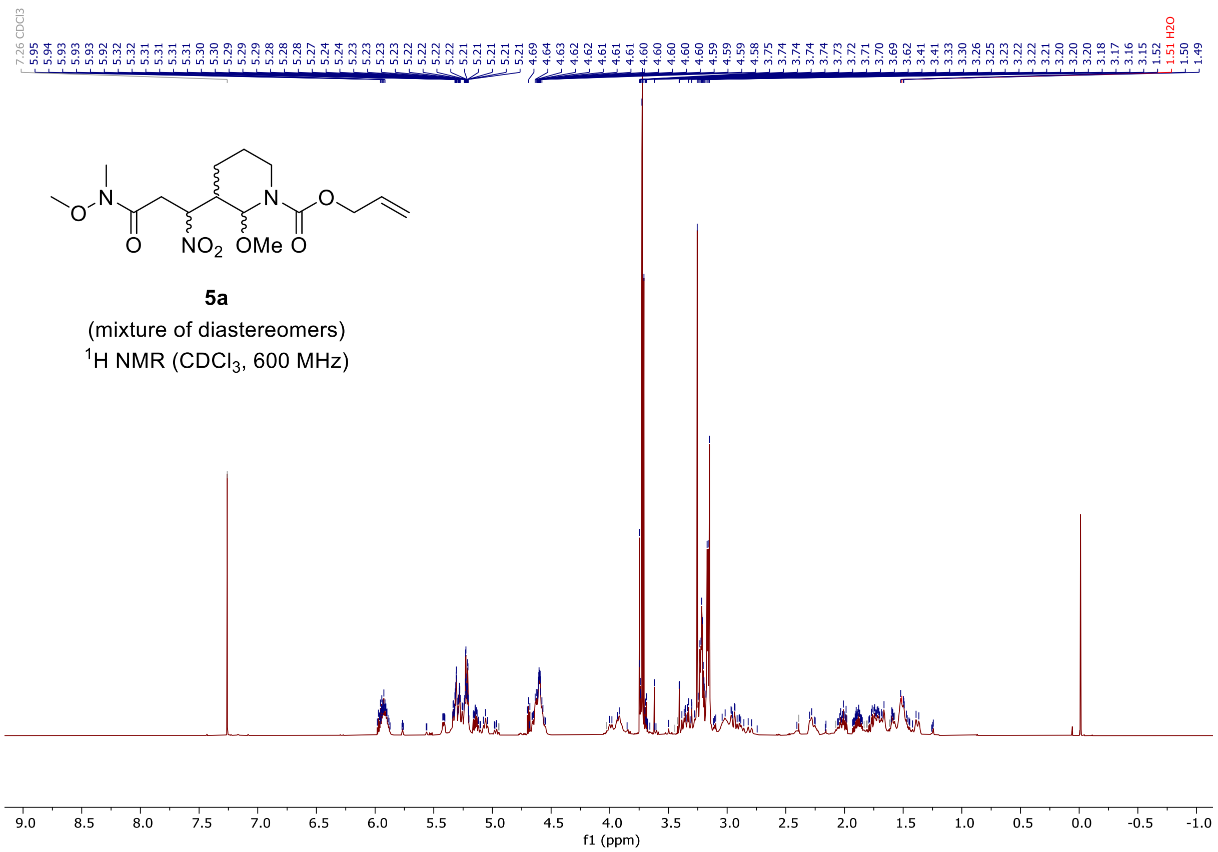

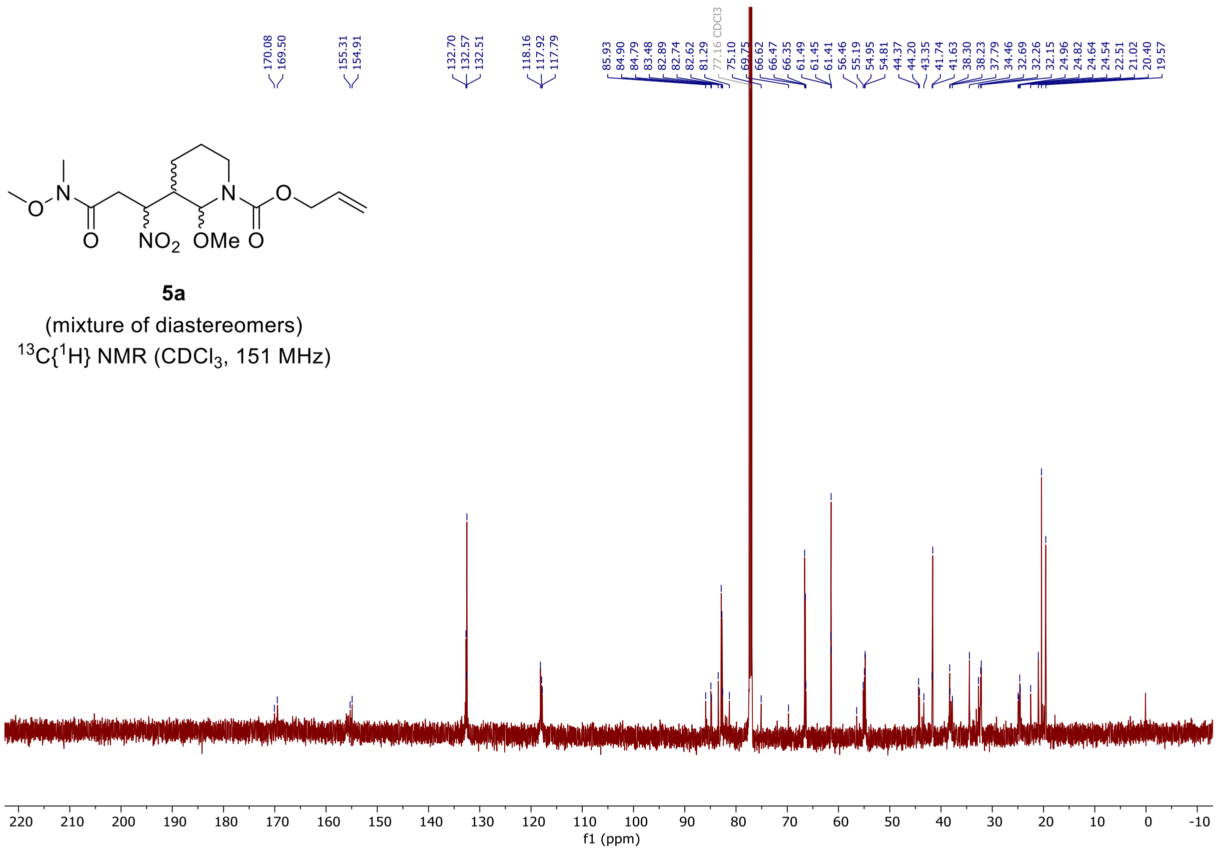

Figure S26

4.7 ^{1}H and $^{13}C\{^{1}H\}$ NMR spectra of 6a

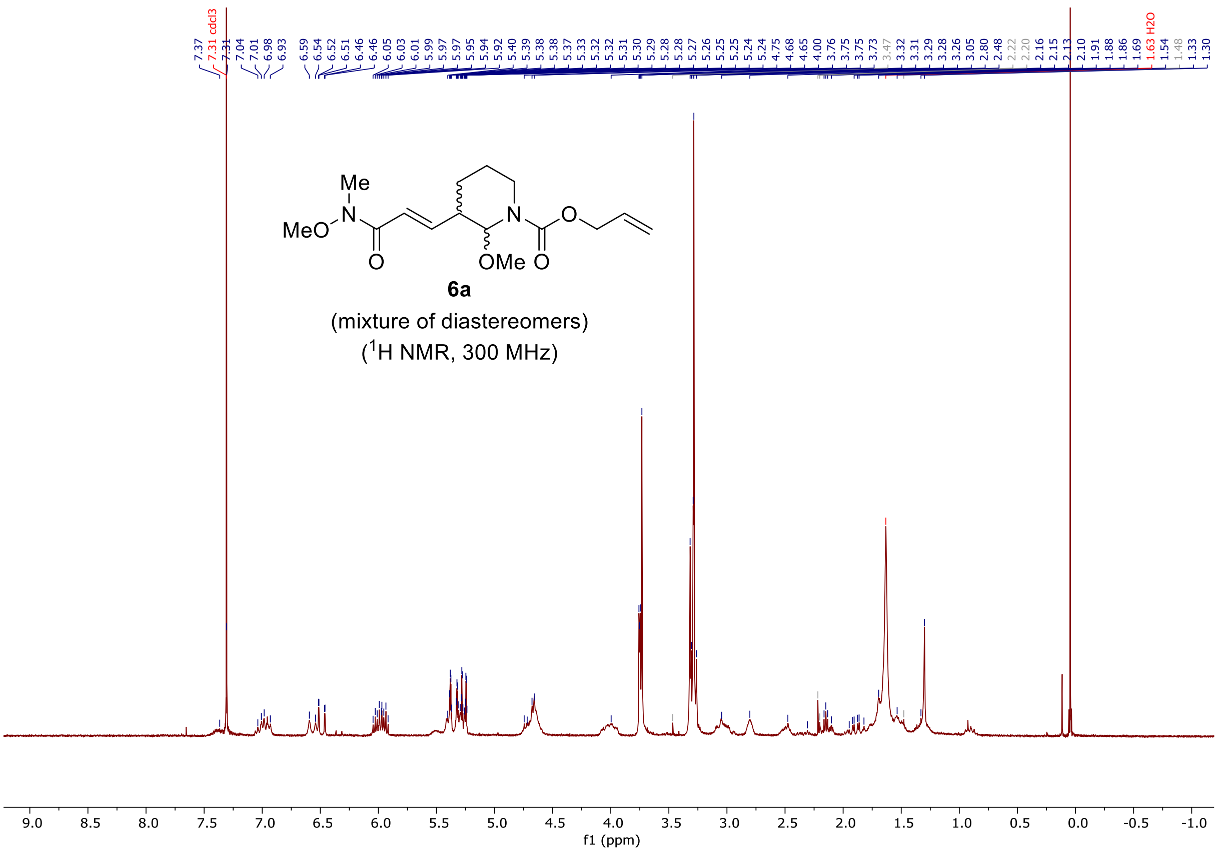

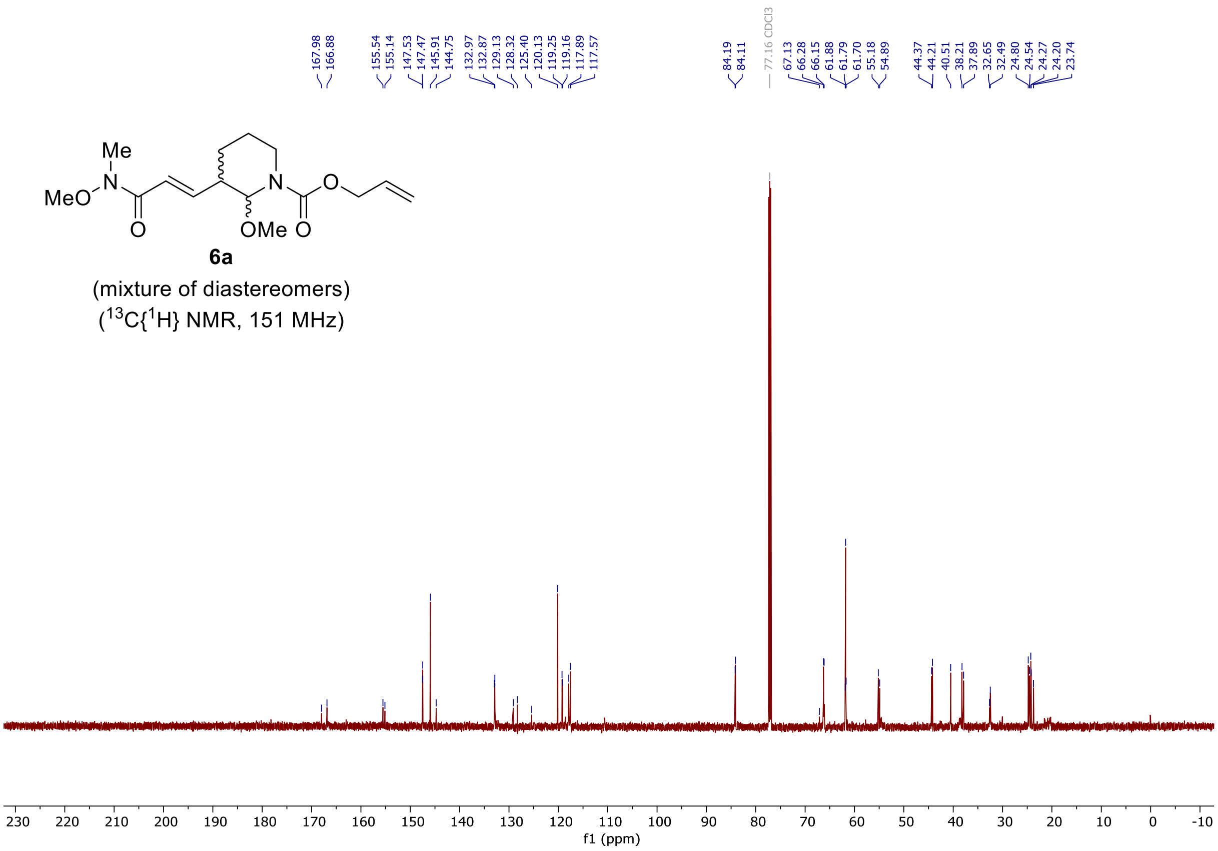

Figure S27

4.8 ^{1}H and ^{13}C{^{1}H} NMR spectra of 3d

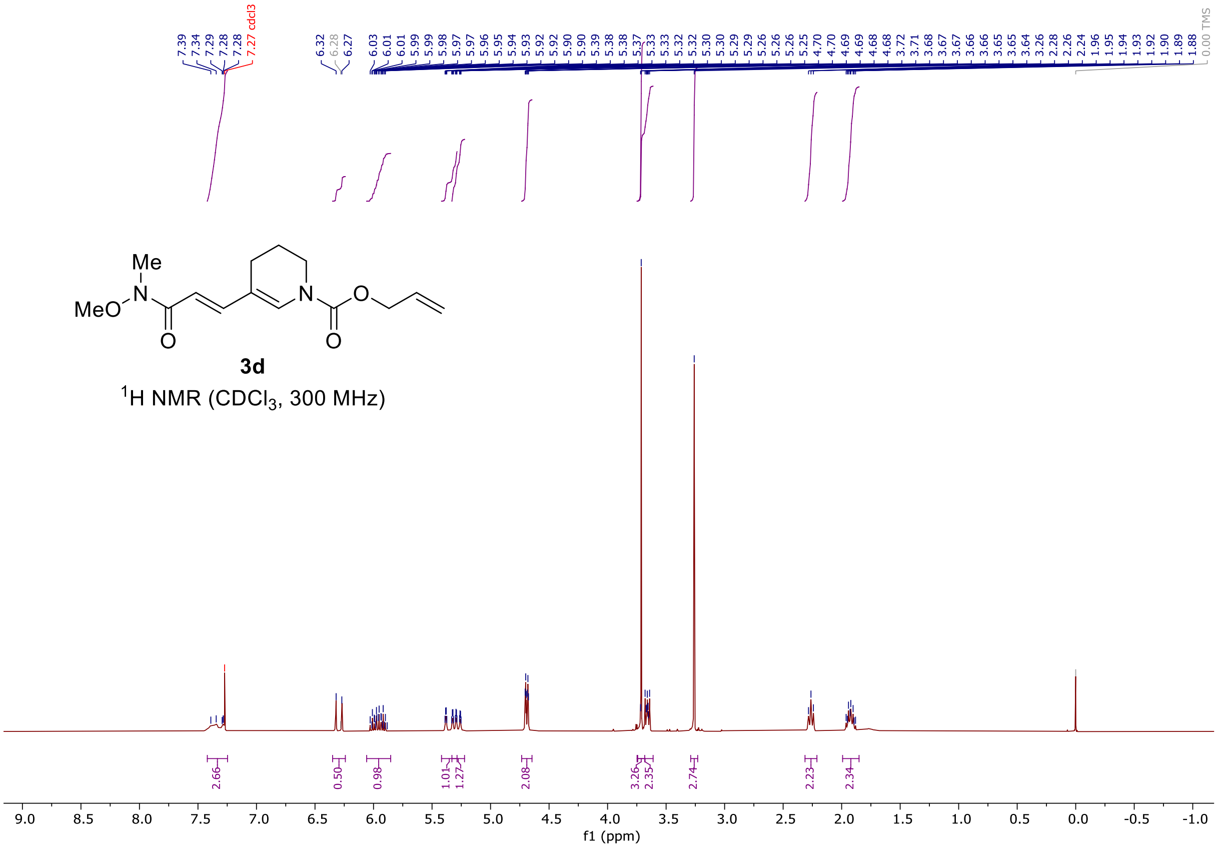

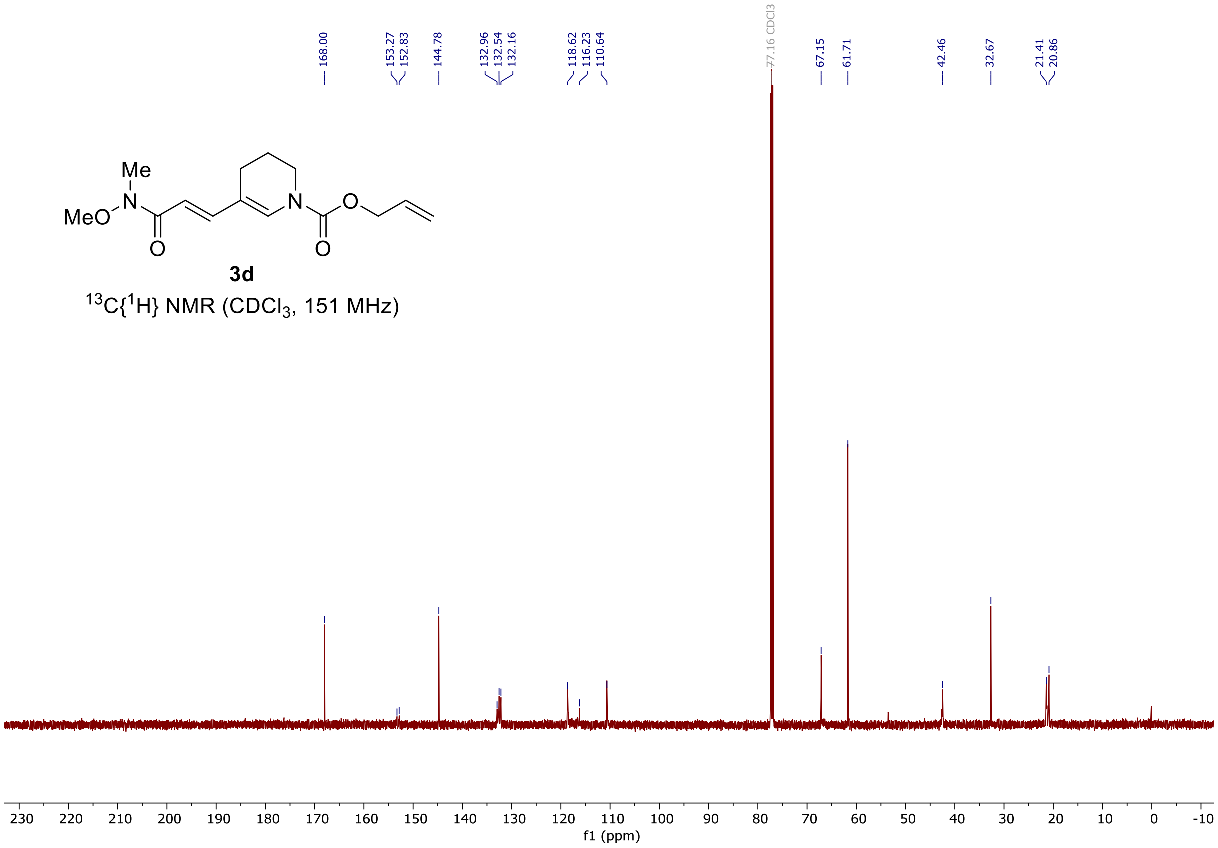

Figure S28

4.9 ^{1}H and ^{13}C{^{1}H} NMR of 3e

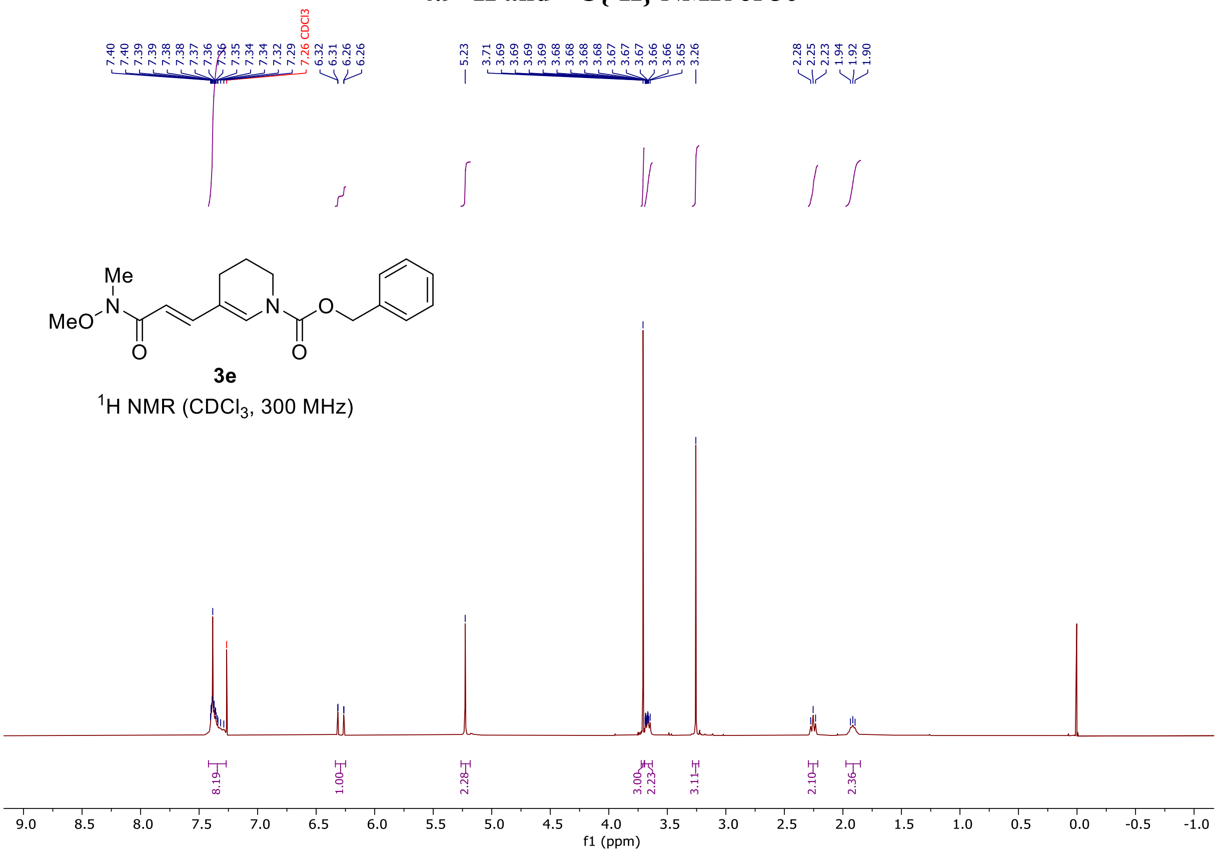

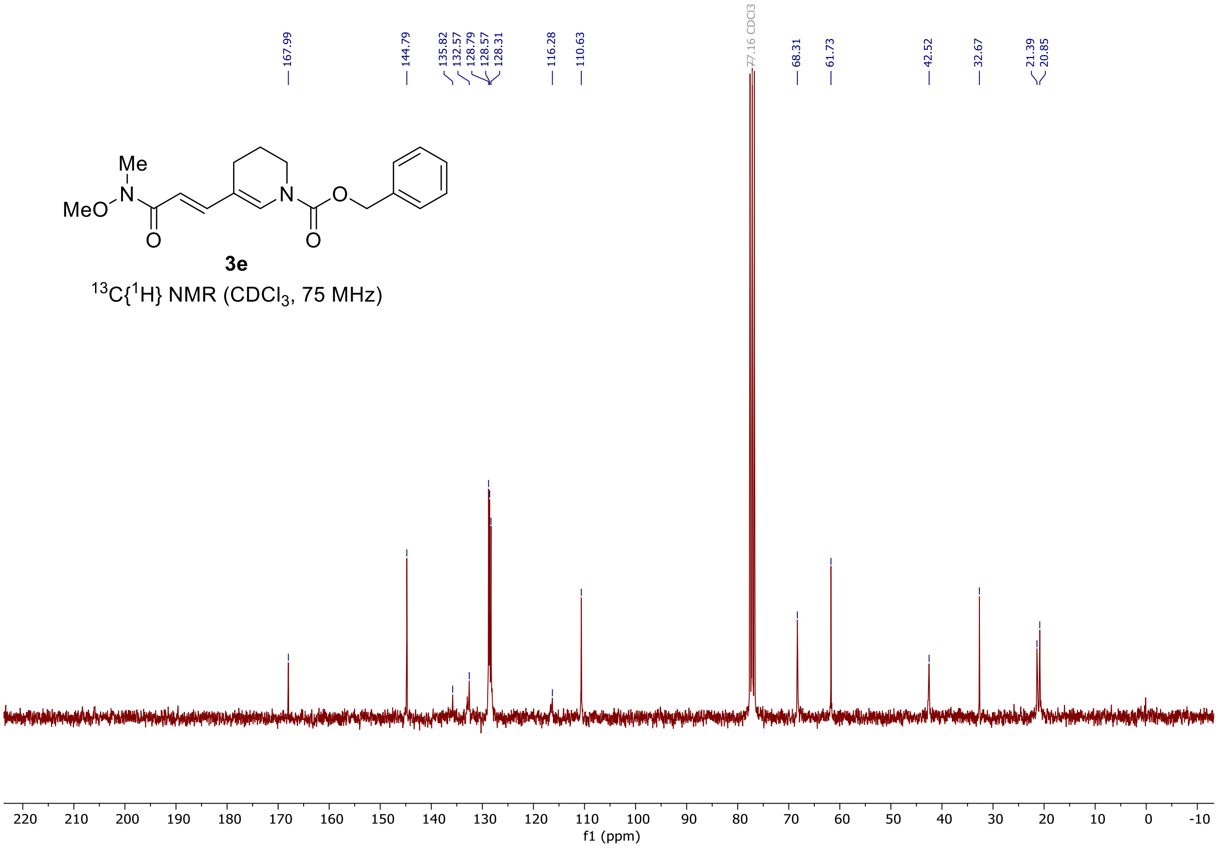

Figure S29

4.10 ^{1}H and ^{13}C{^{1}H} NMR spectra of 3f

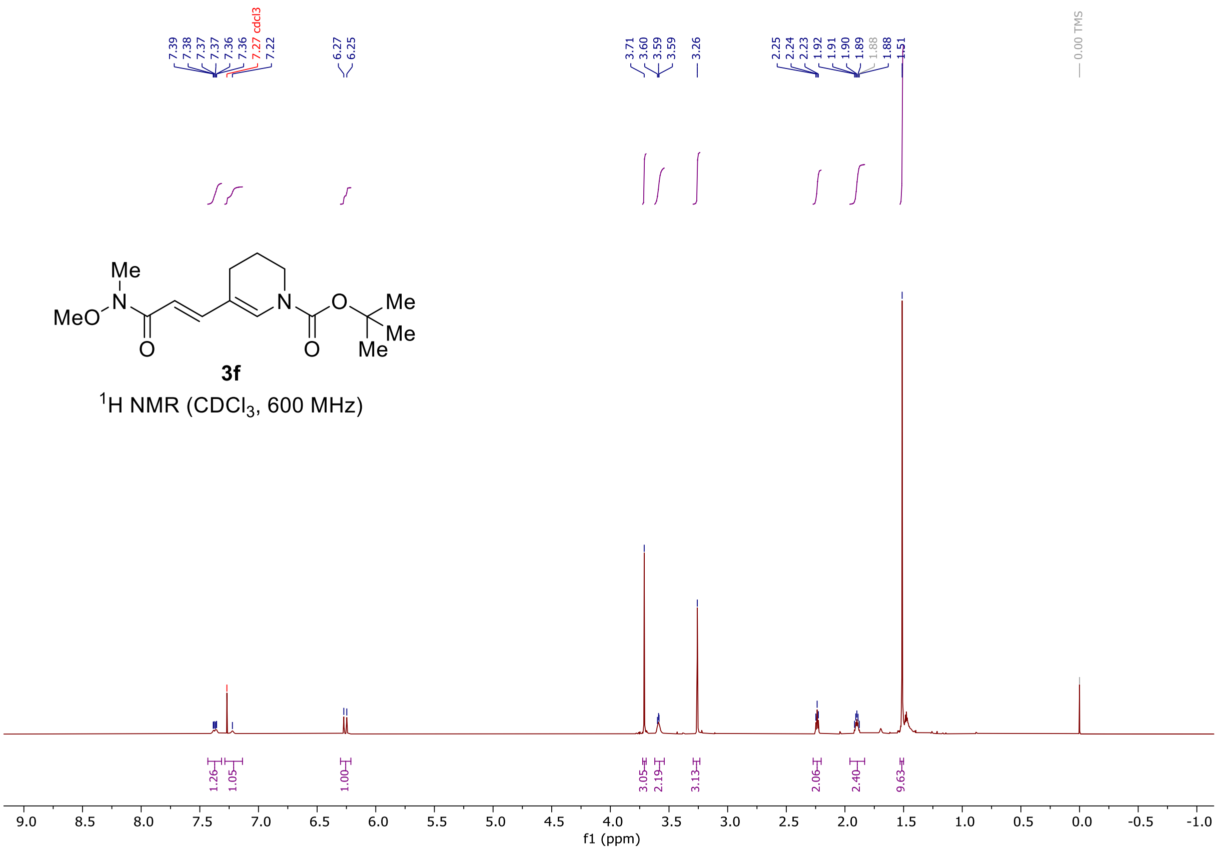

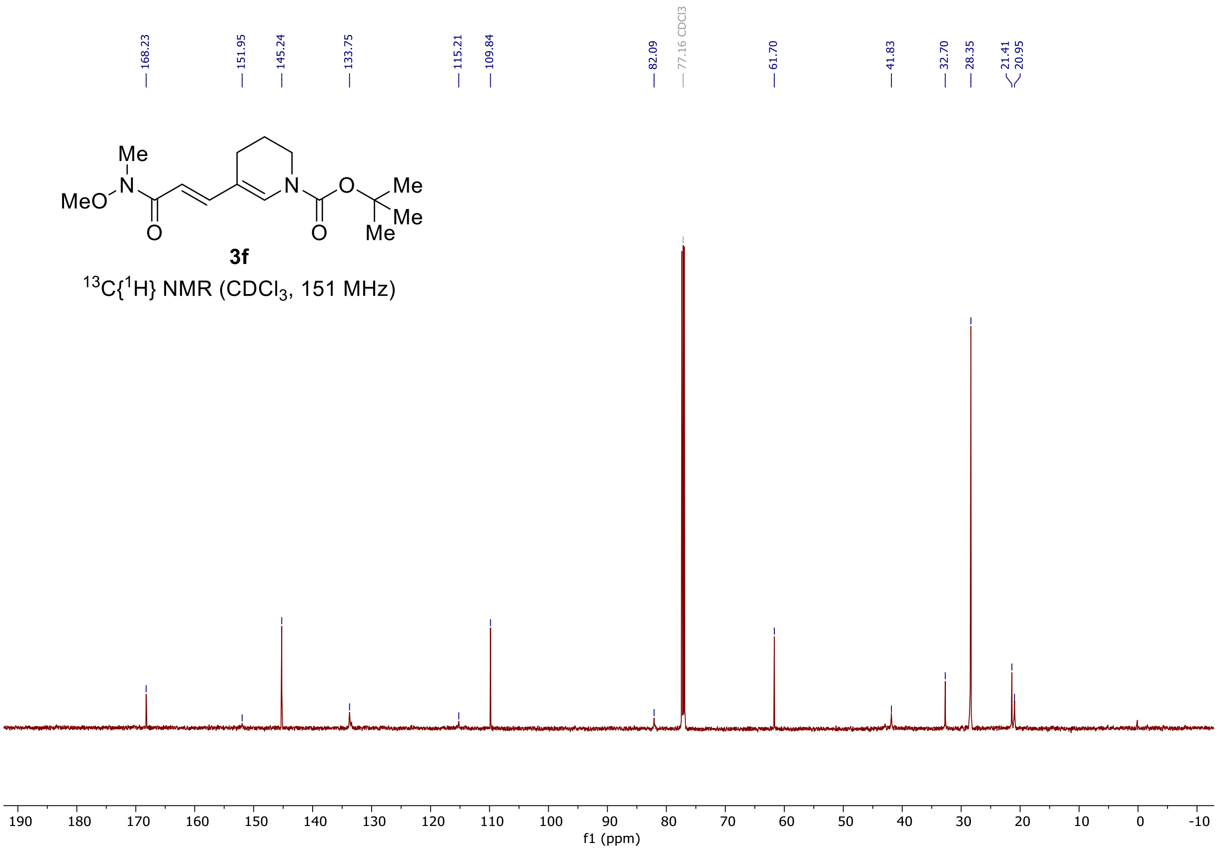

Figure S30

4.11 ^{1}H and ^{13}C{^{1}H} NMR spectra of 3g

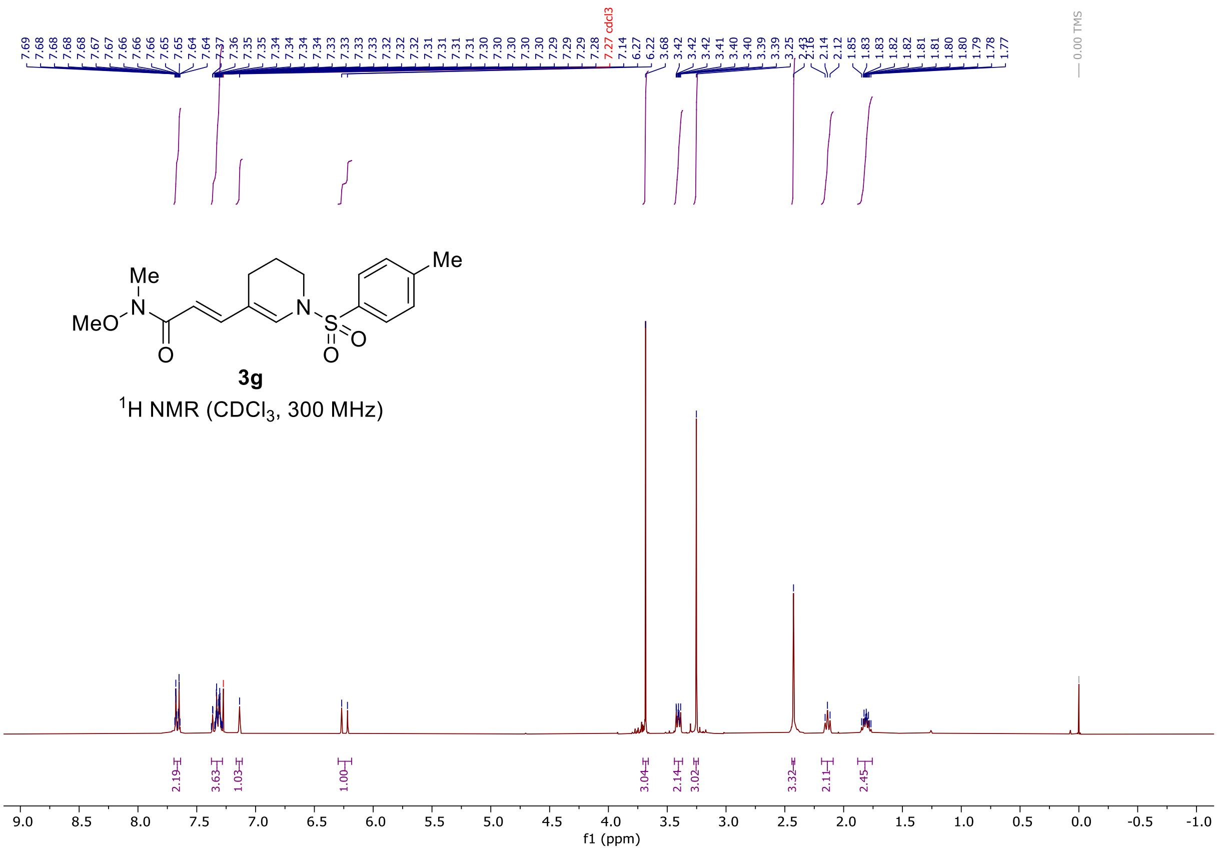

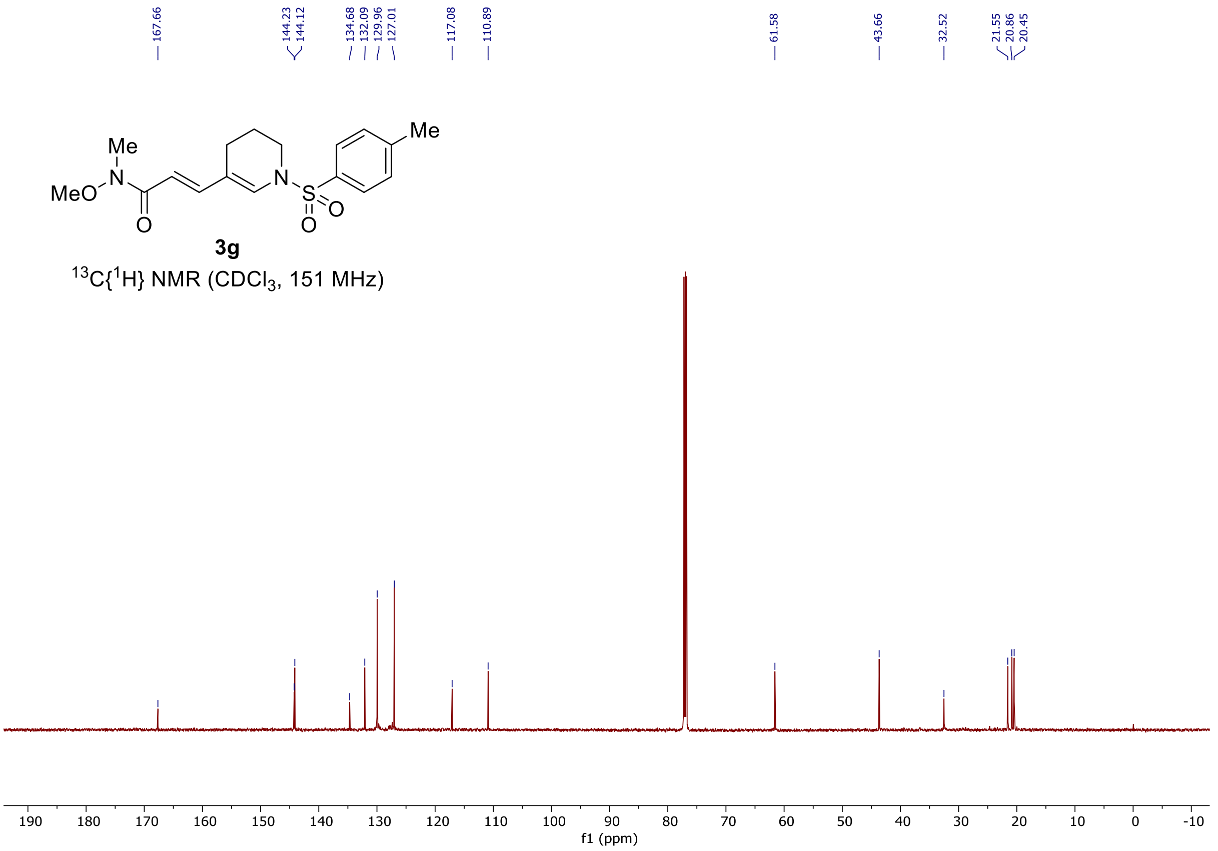

Figure S31

4.12 ^{1}H and ^{13}C{^{1}H} NMR spectra of 3h

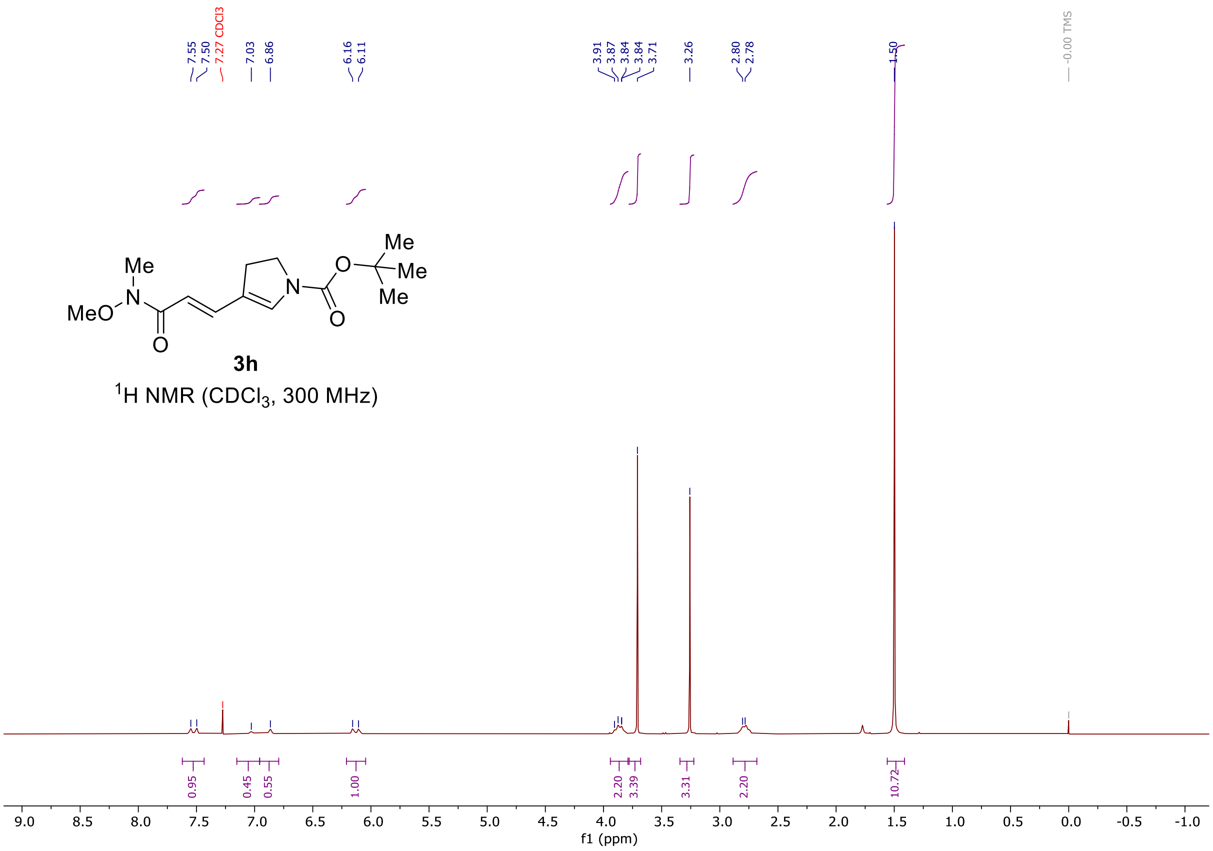

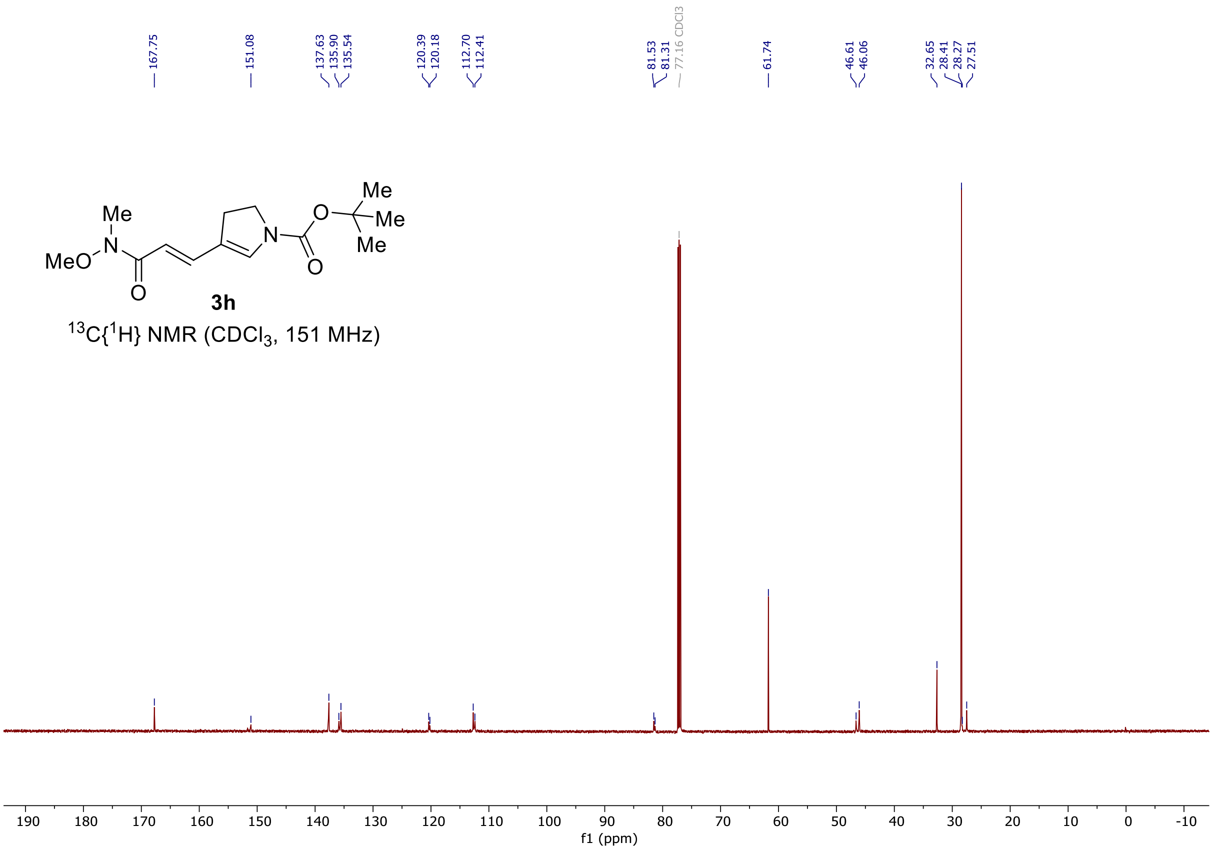

Figure S32

4.13 ^{1}H and $^{13}C\{^{1}H\}$ NMR spectra of 3i

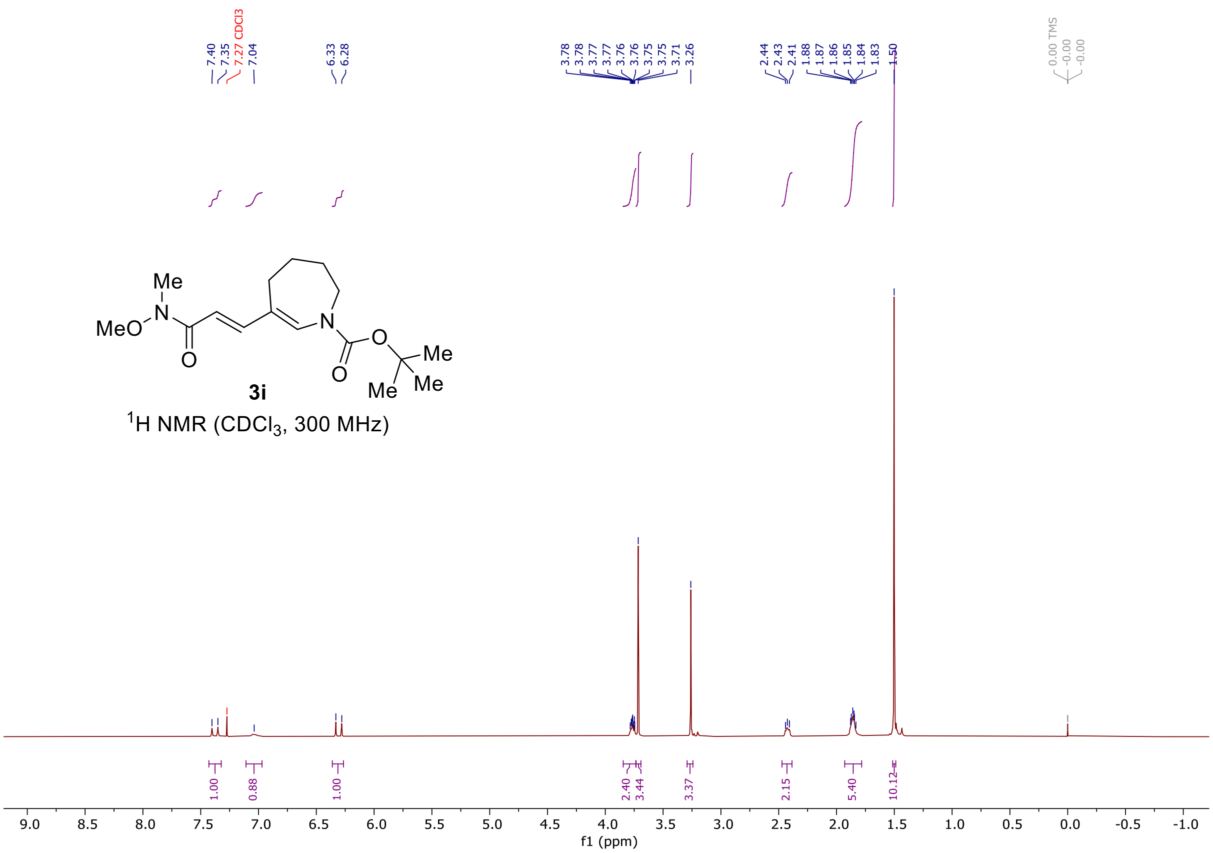

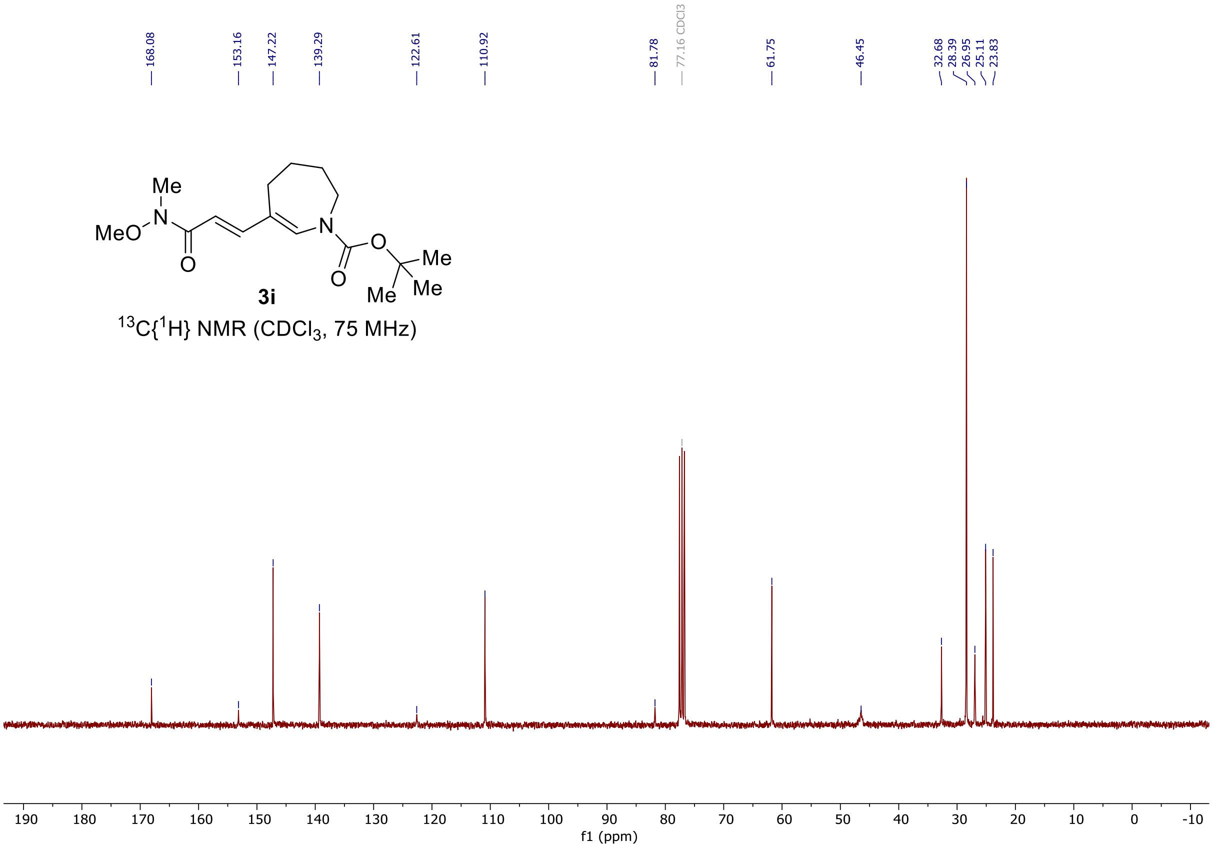

Figure S33

4.14 ^{1}H and ^{13}C{^{1}H} NMR spectra of 3j

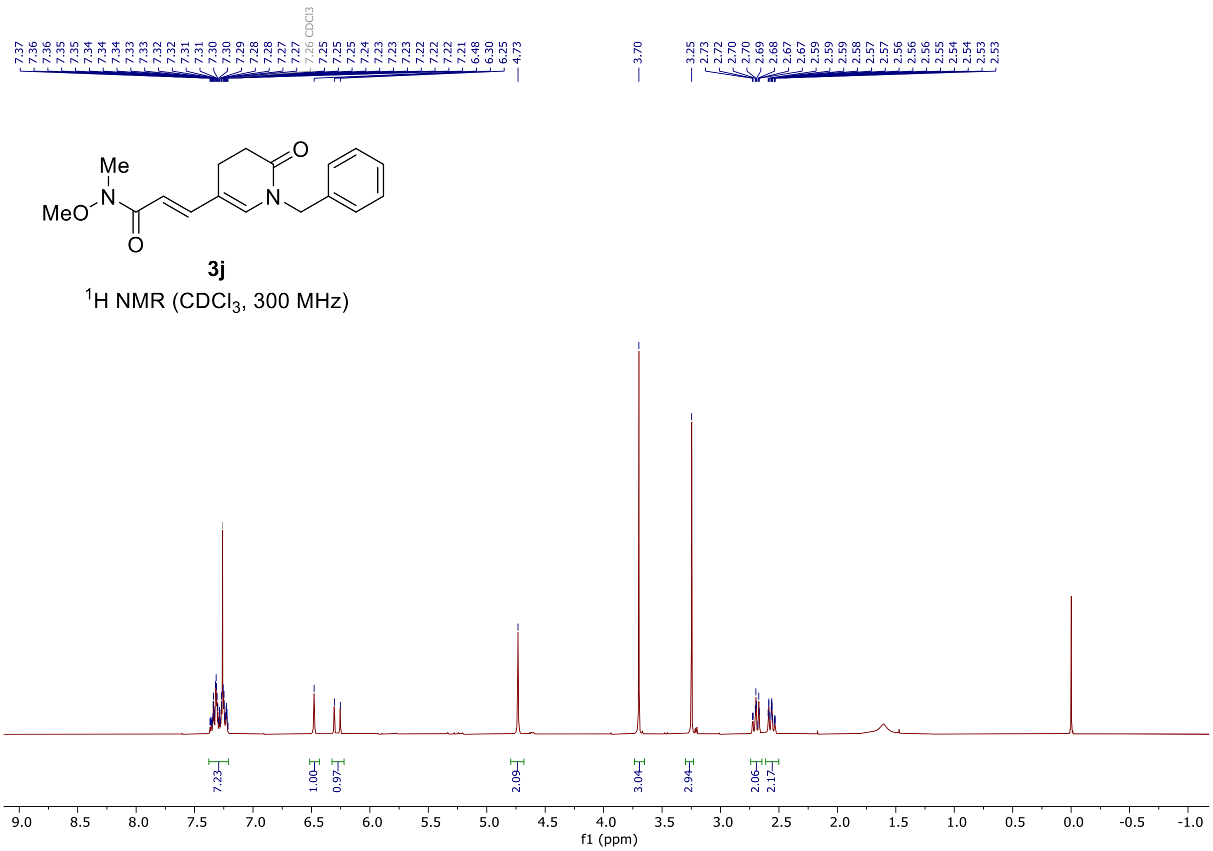

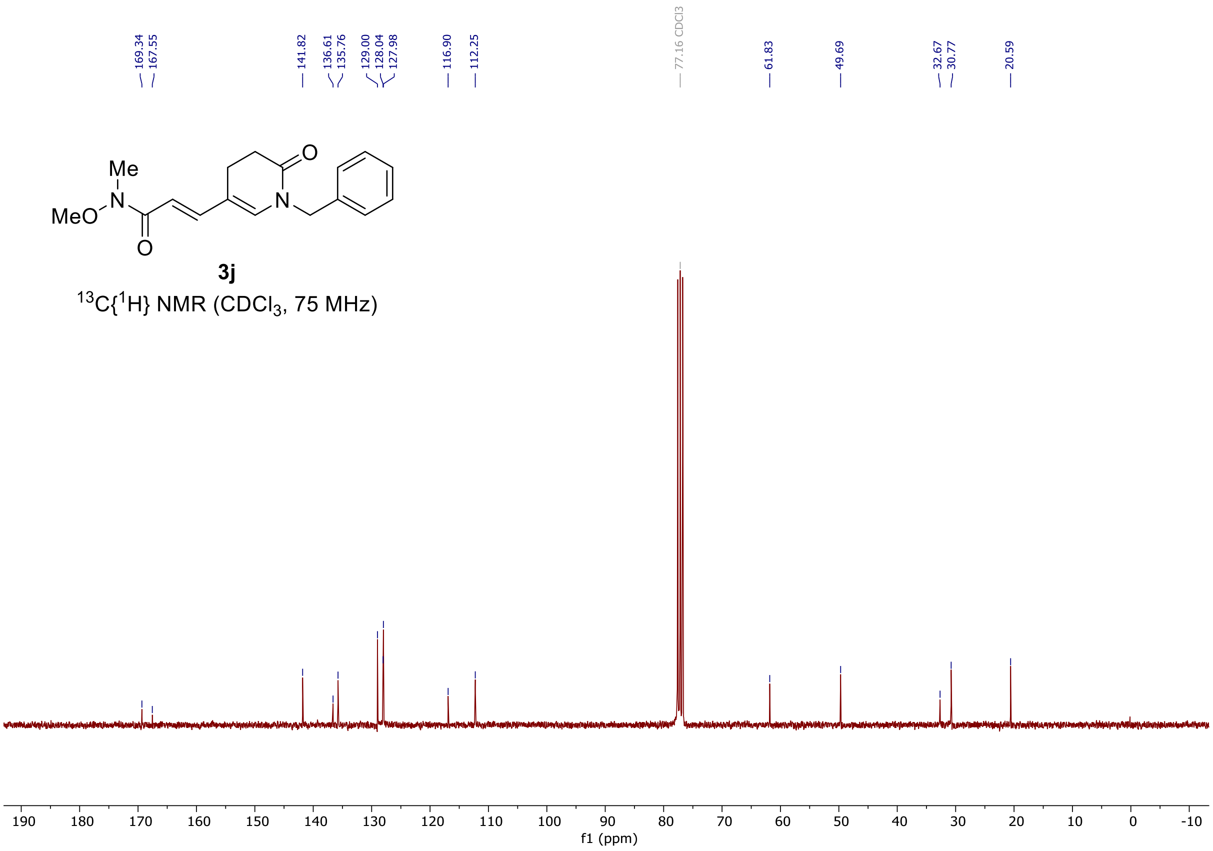

Figure S34

4.15 ^{1}H and ^{13}C{^{1}H} NMR spectra of 3k

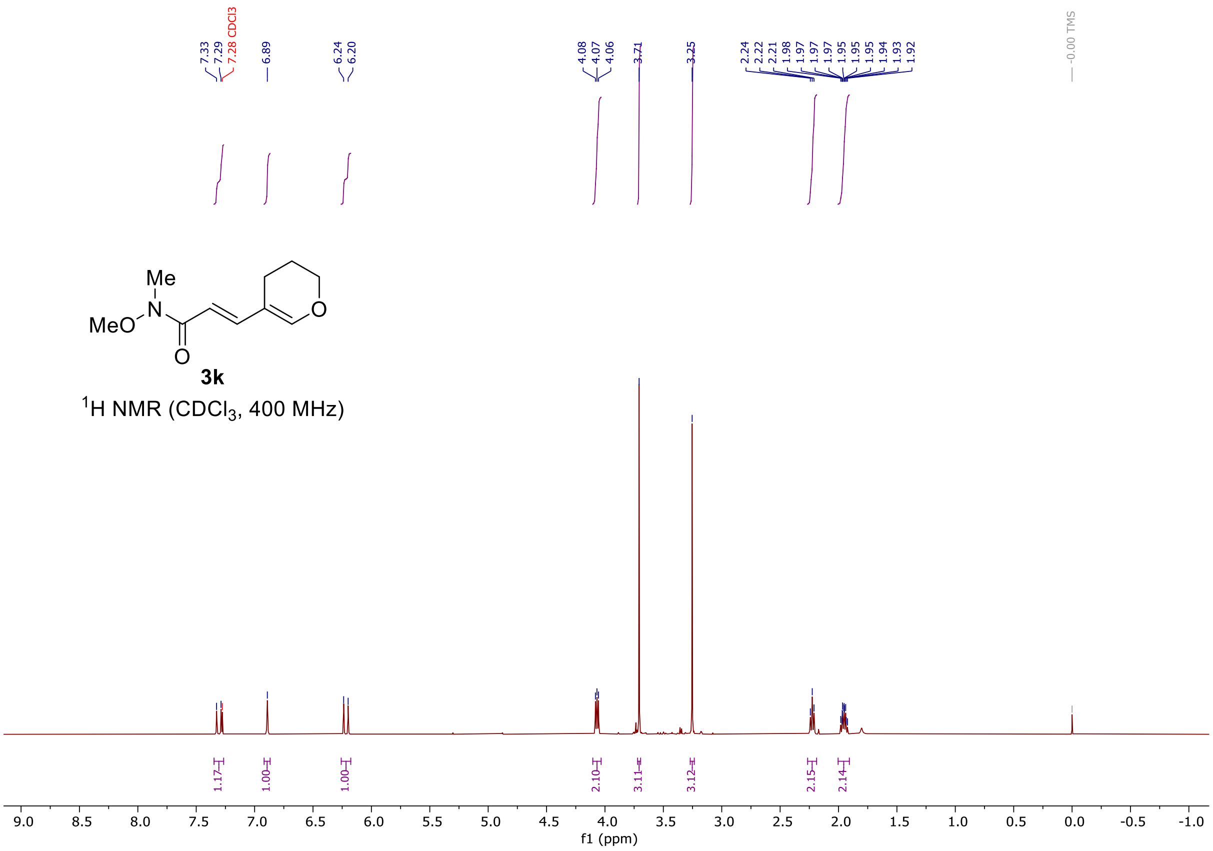

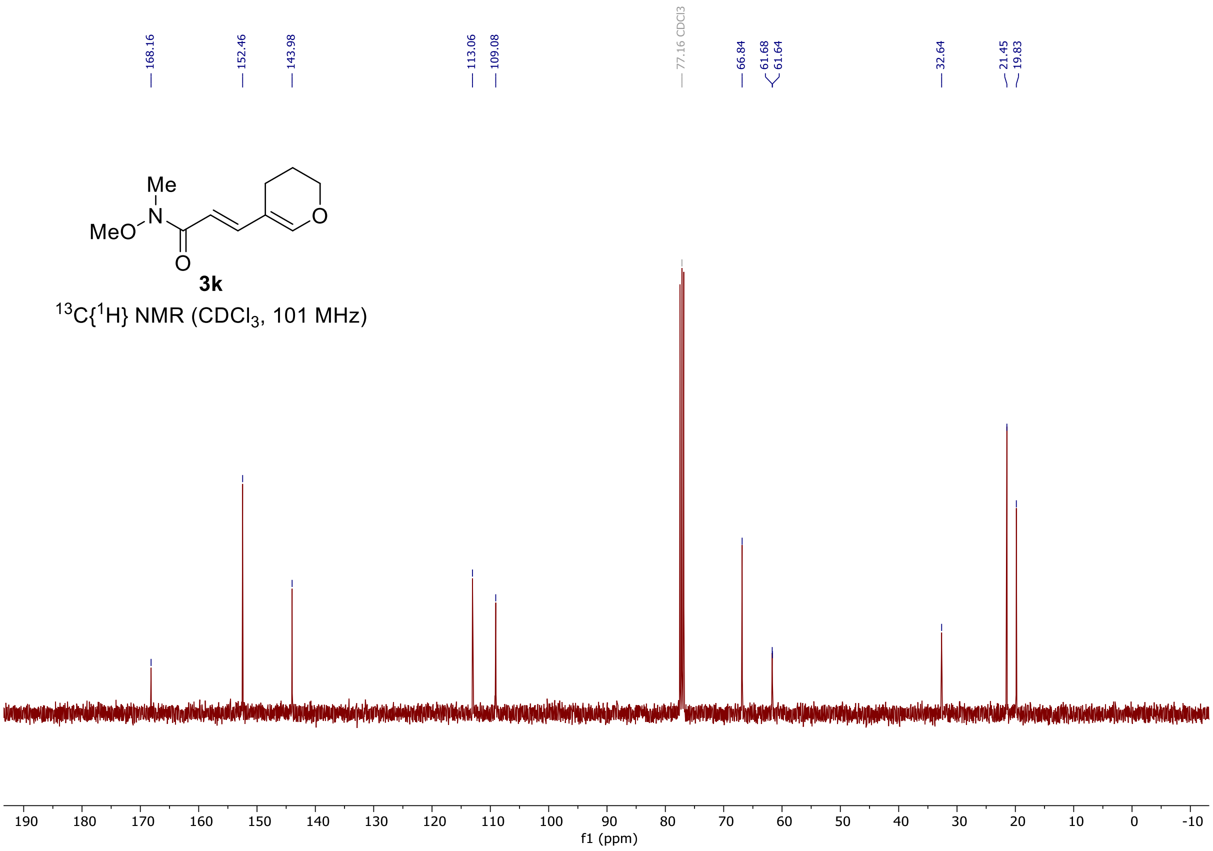

Figure S35

4.16 ^{1}H and ^{13}C{^{1}H} NMR spectra of 3l

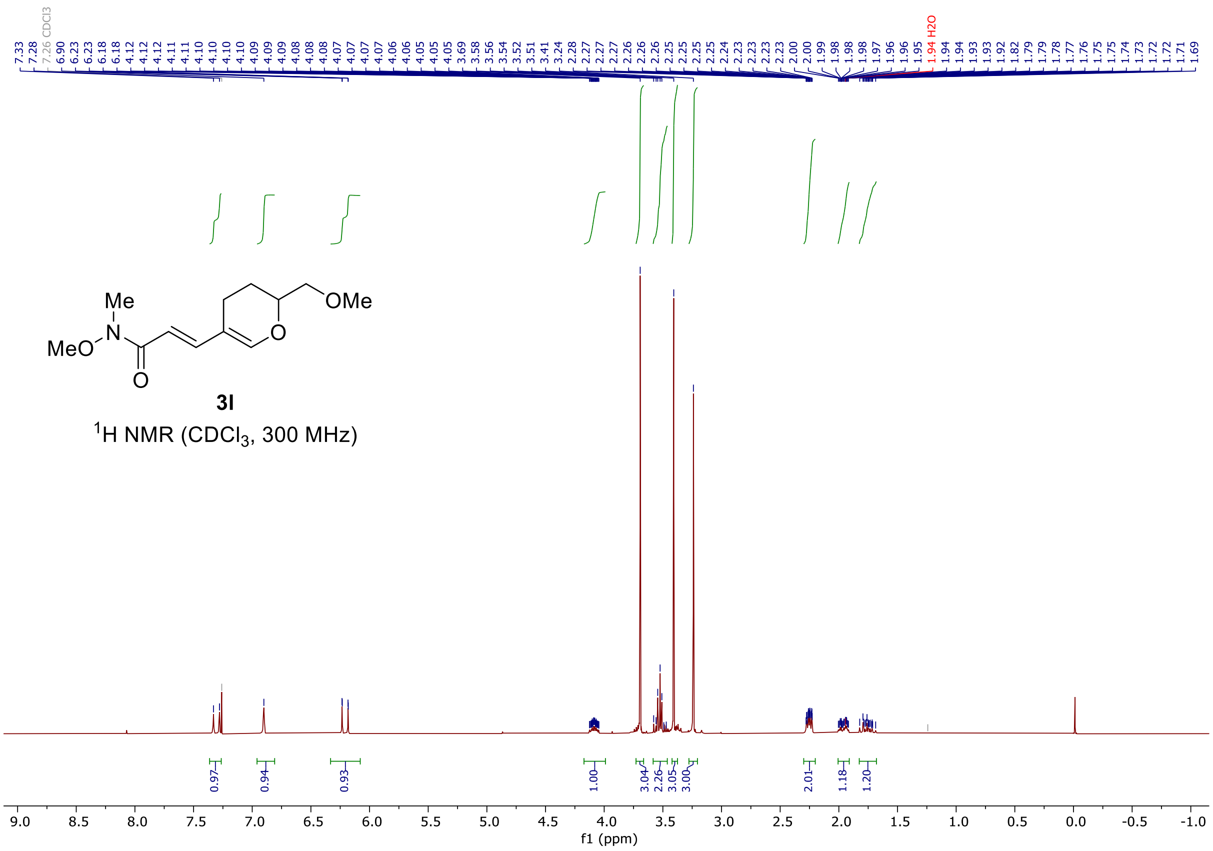

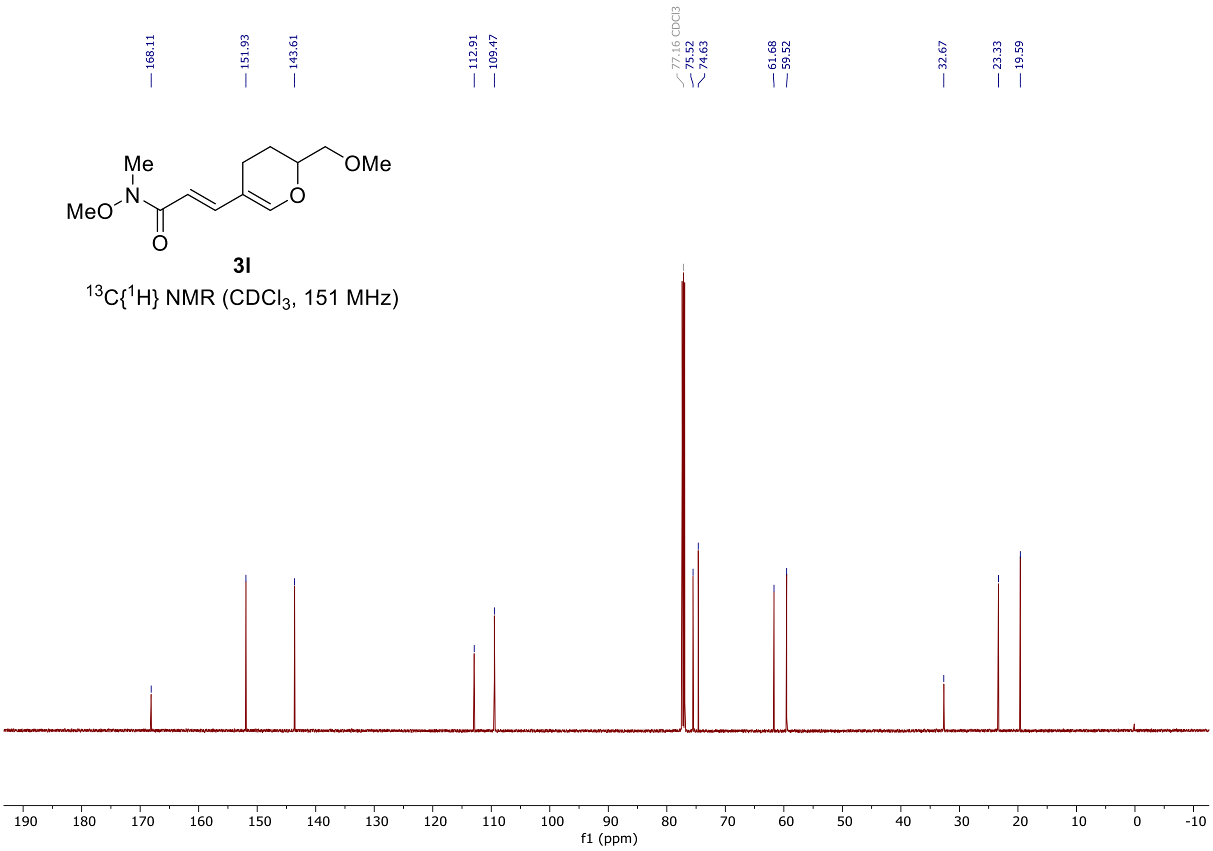

Figure S36

4.17 ^{1}H and $^{13}C\{^{1}H\}$ NMR spectra of 3m

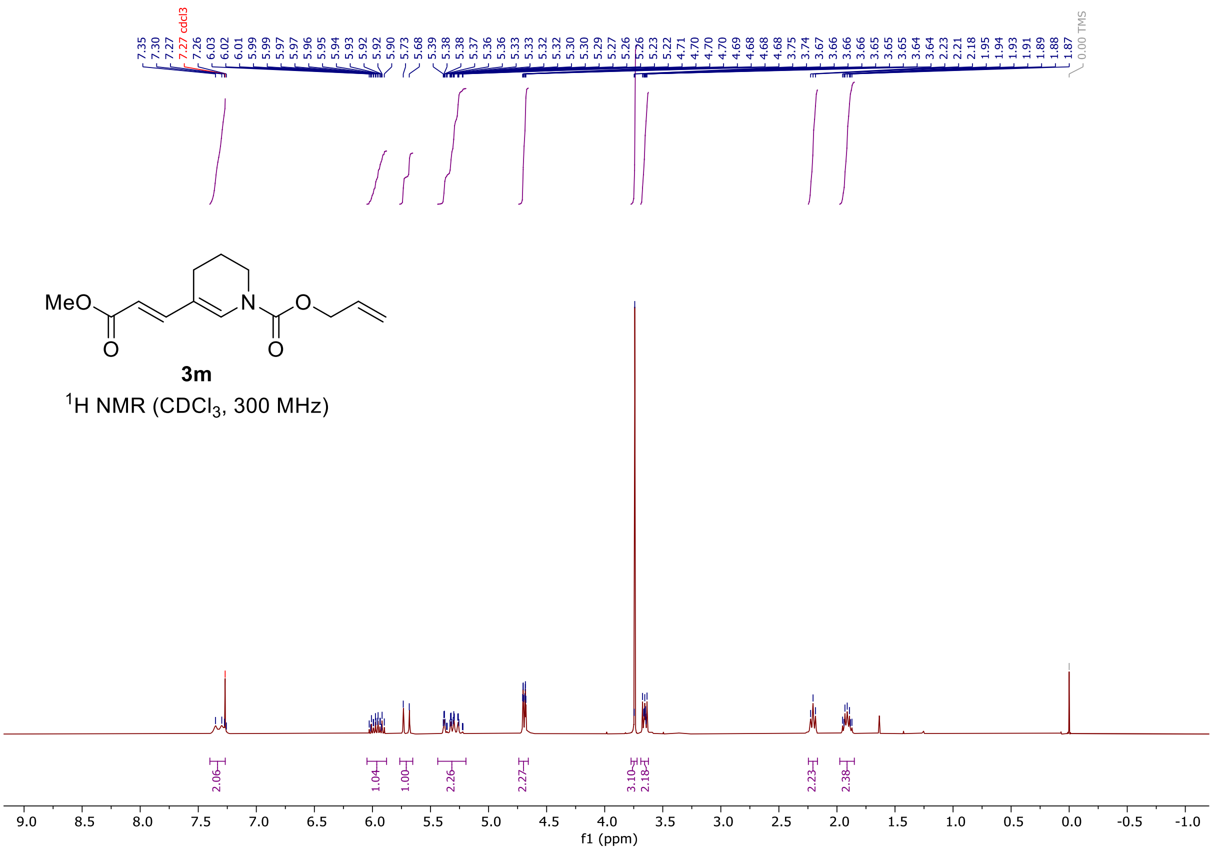

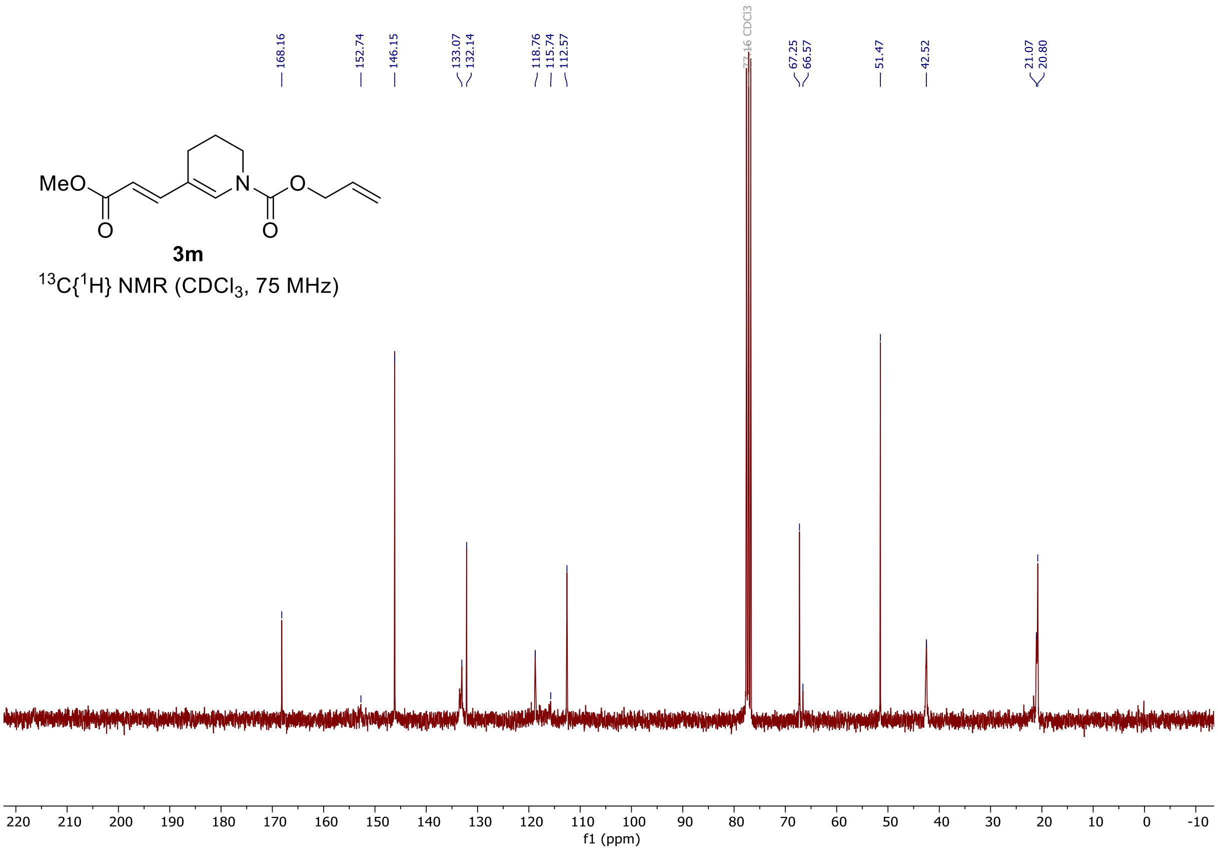

Figure S37

4.18 ^{1}H and ^{13}C{^{1}H} NMR spectra of 3n

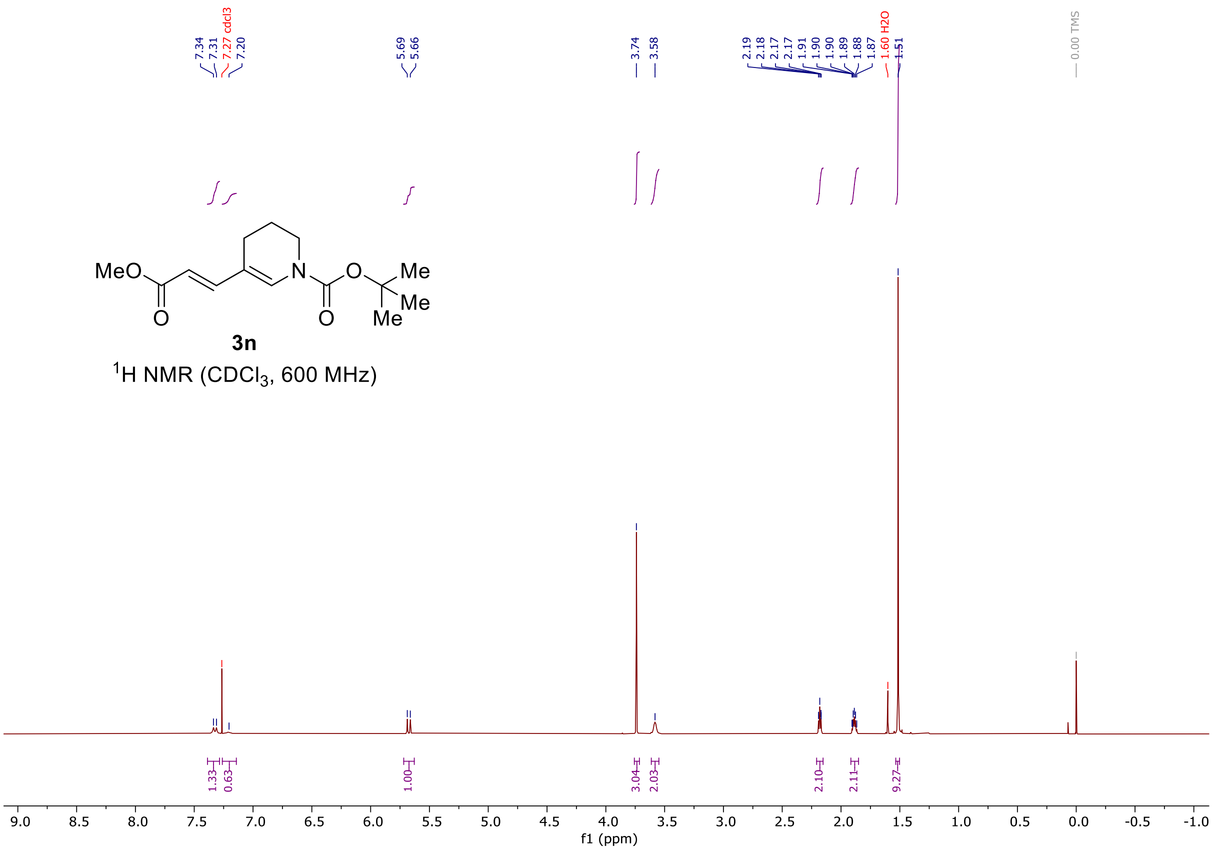

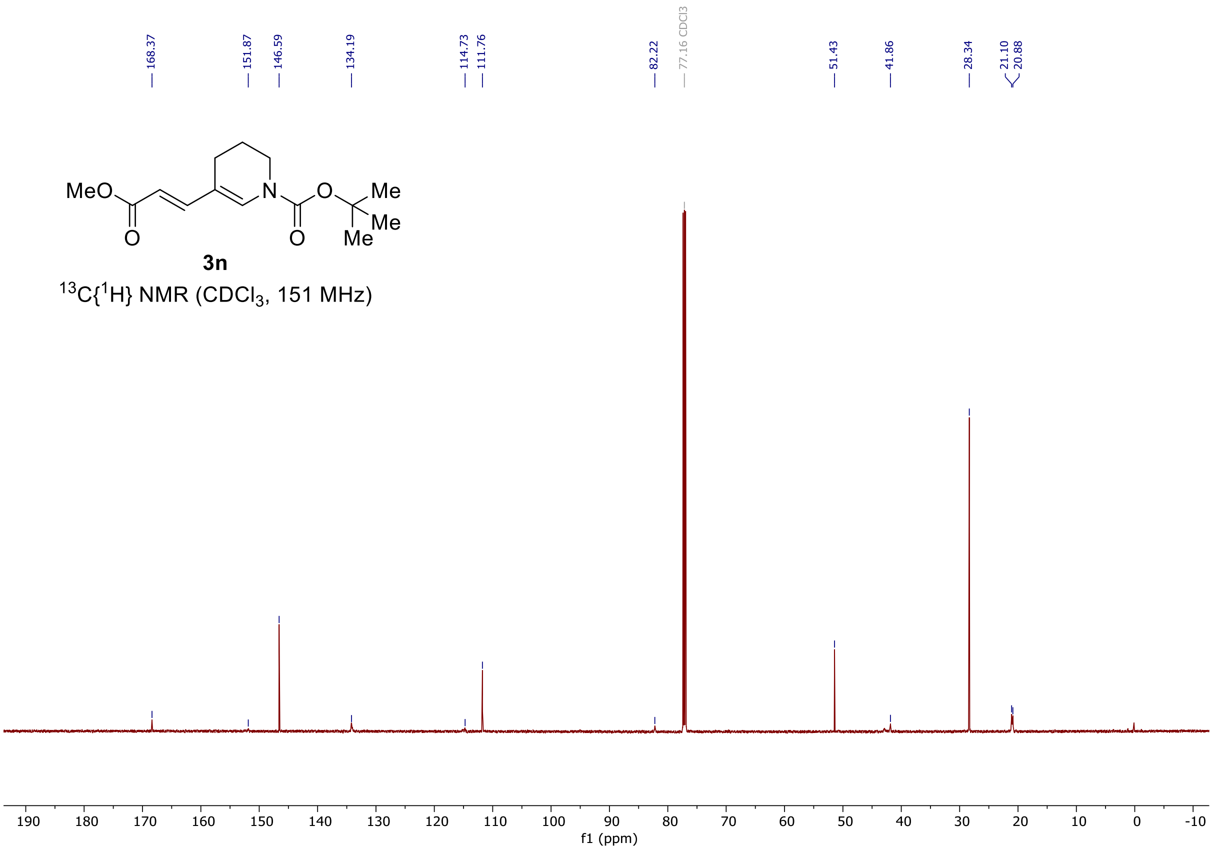

Figure S38

4.19 ^{1}H and ^{13}C{^{1}H} NMR spectra of 3o

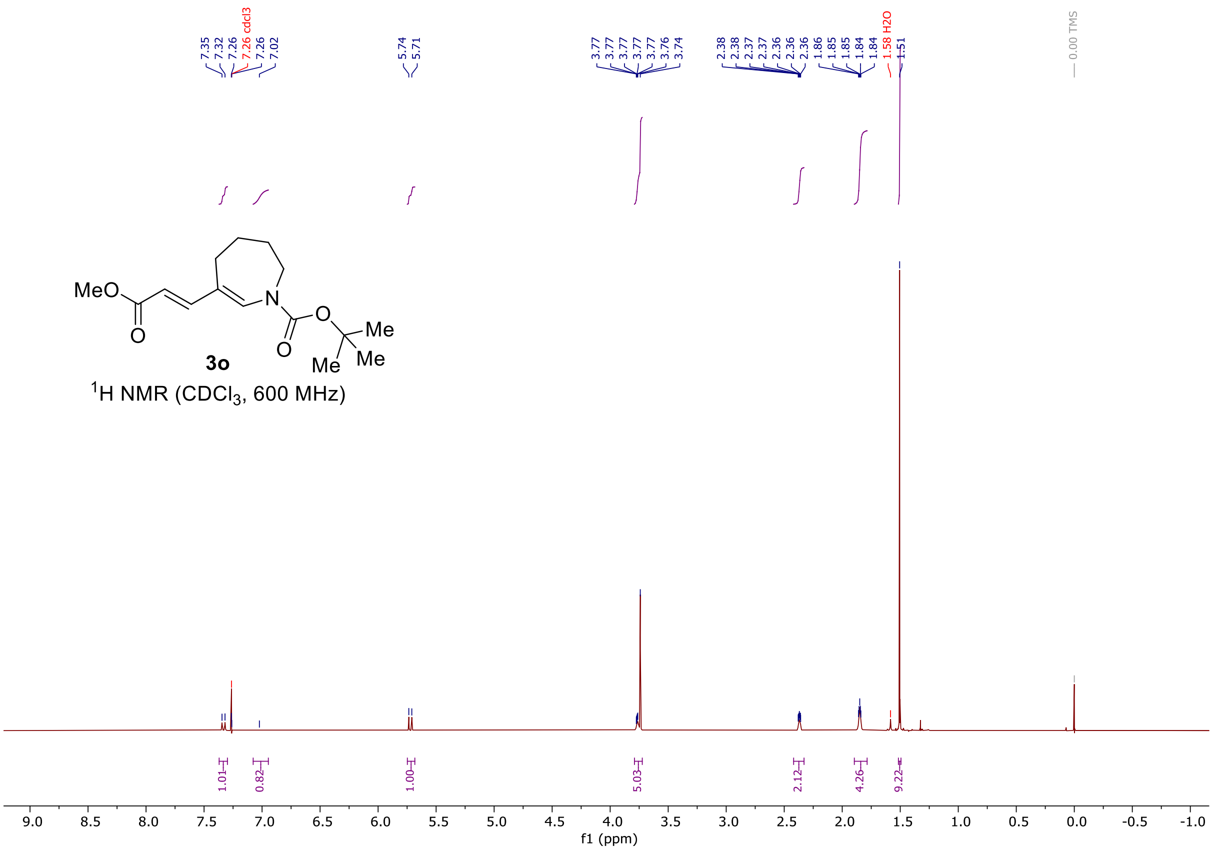

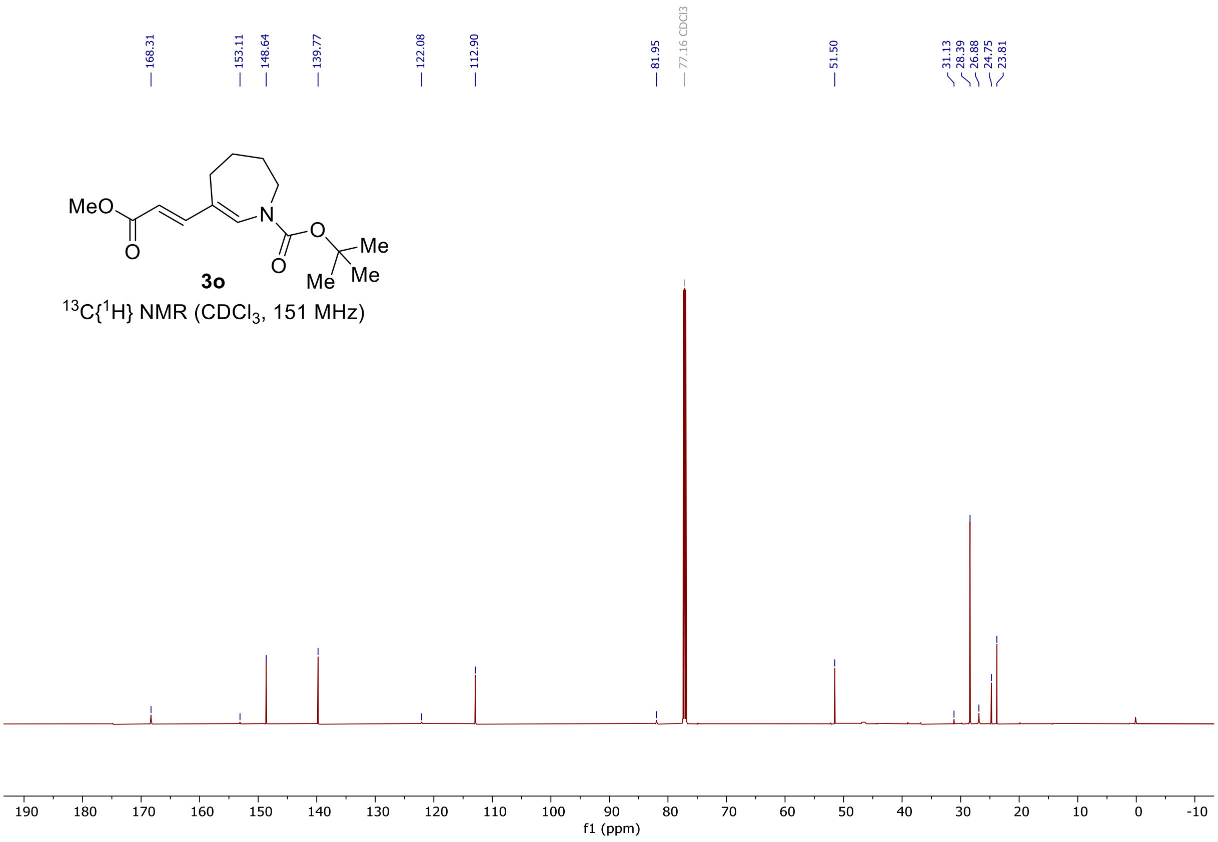

Figure S39

4.20 ^{1}H and ^{13}C{^{1}H} NMR spectra of 3p

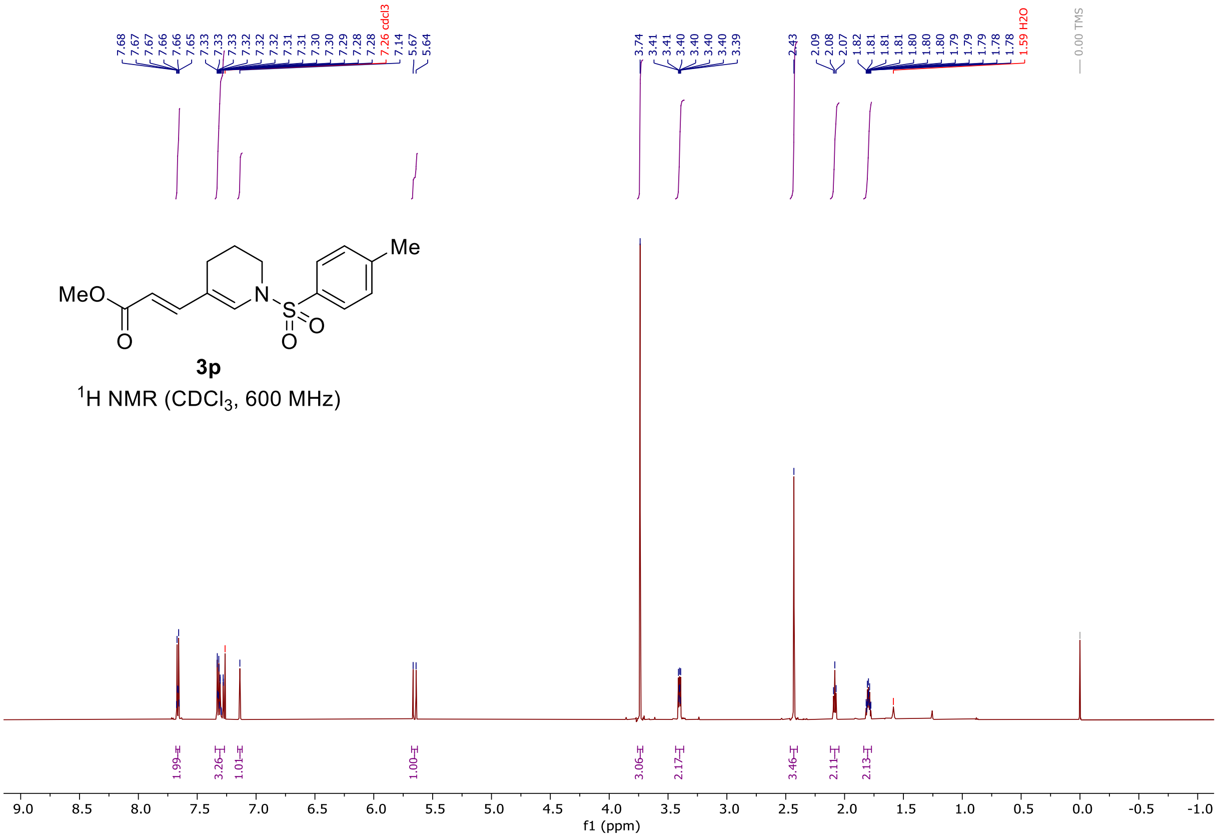

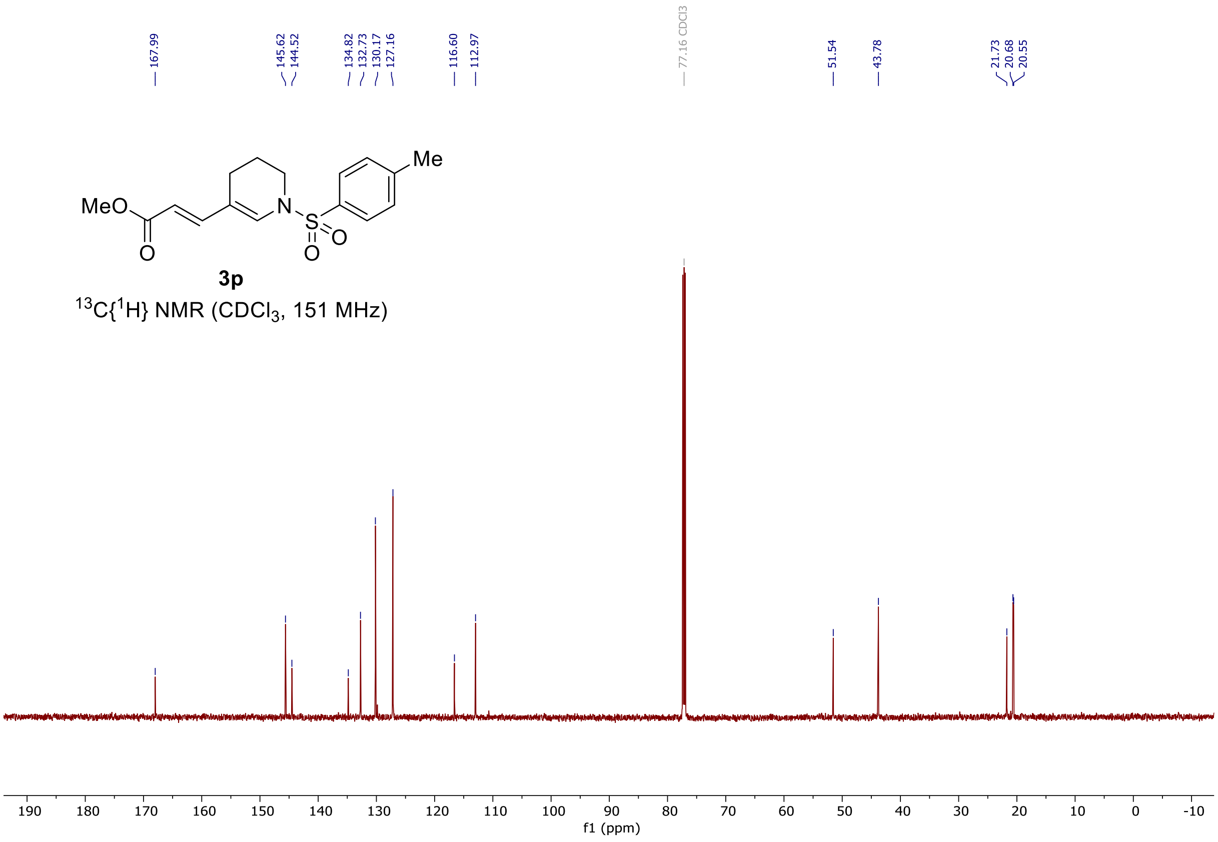

Figure S40

4.21 ^{1}H and $^{13}C\{^{1}H\}$ NMR spectra of 3q

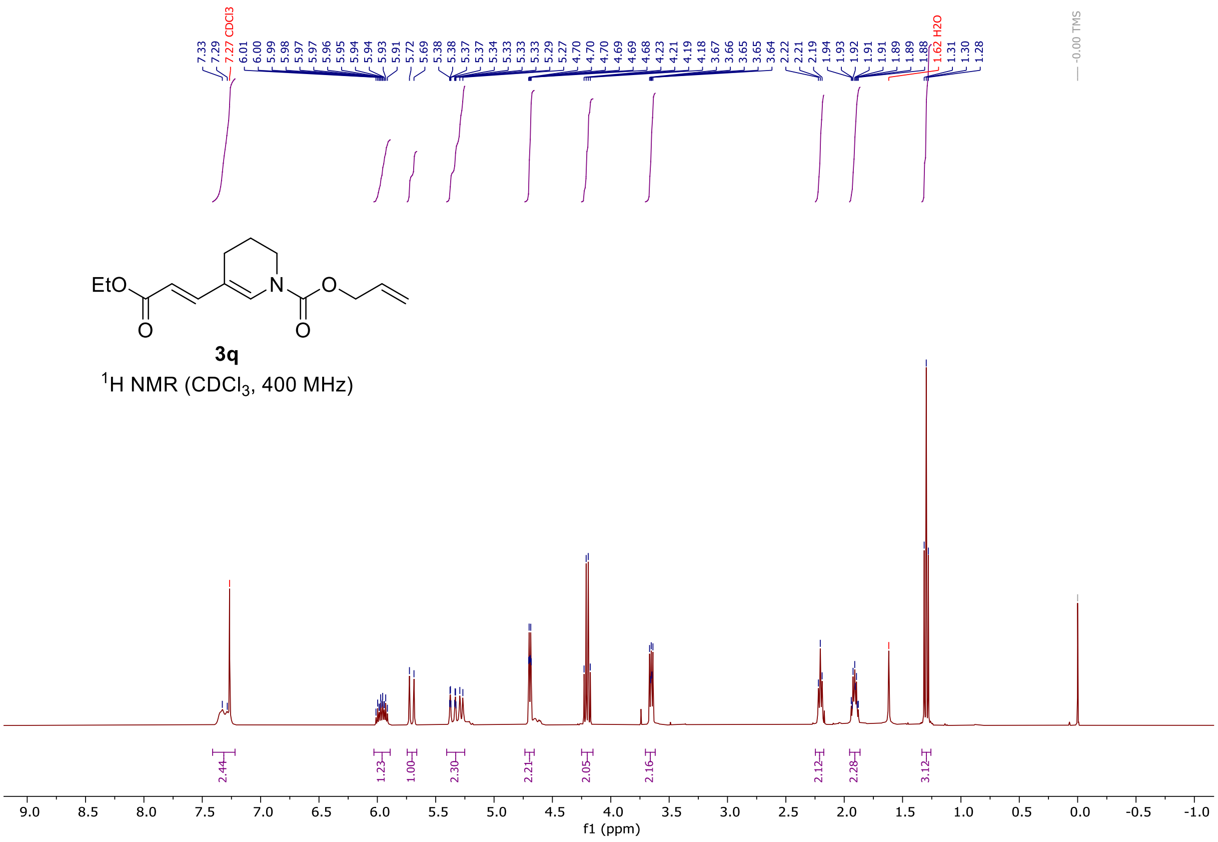

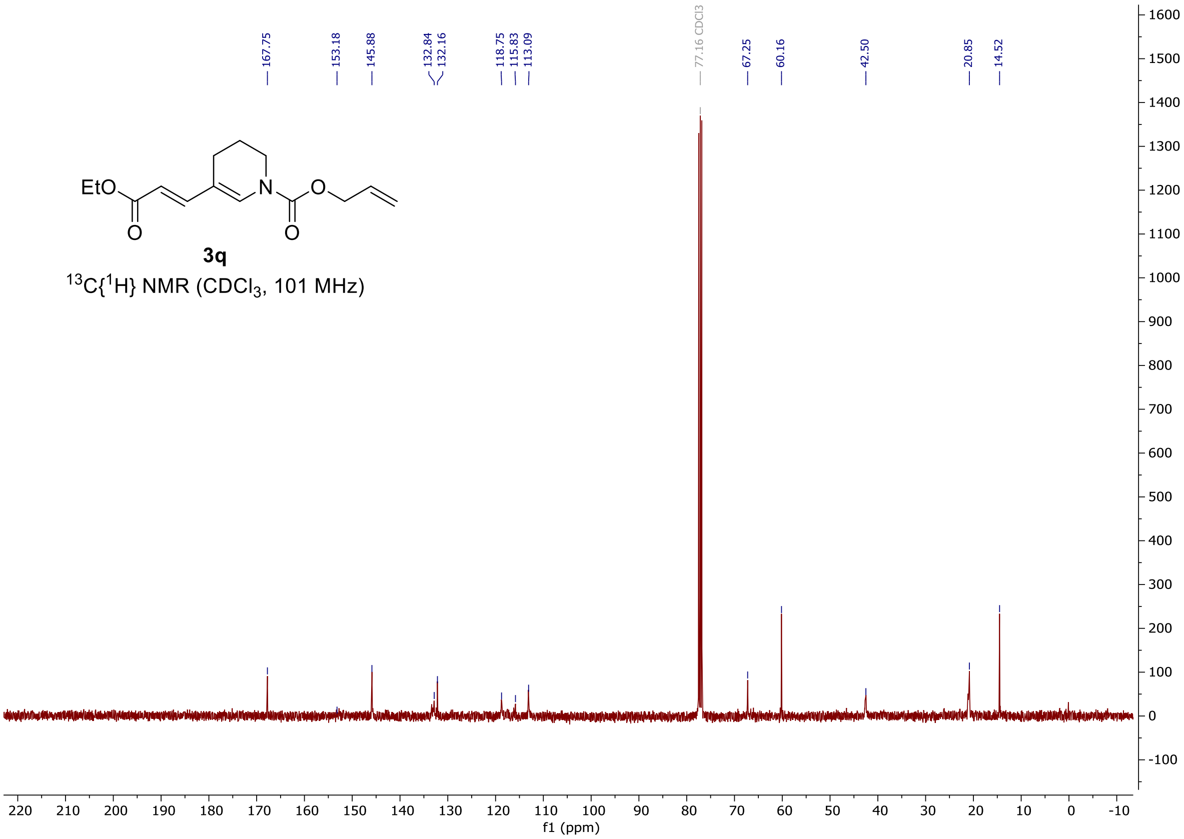

Figure S41

4.22 ^{1}H and ^{13}C{^{1}H} NMR spectra of 3r

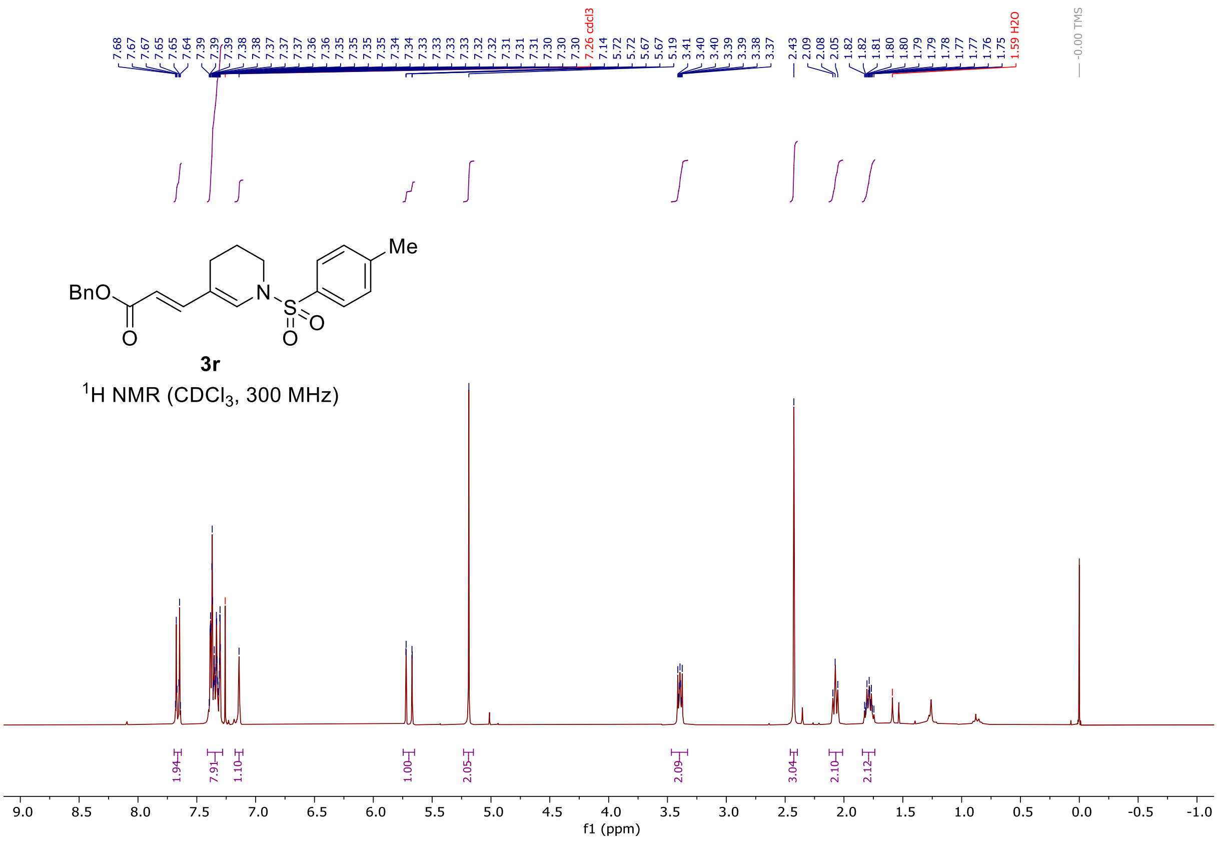

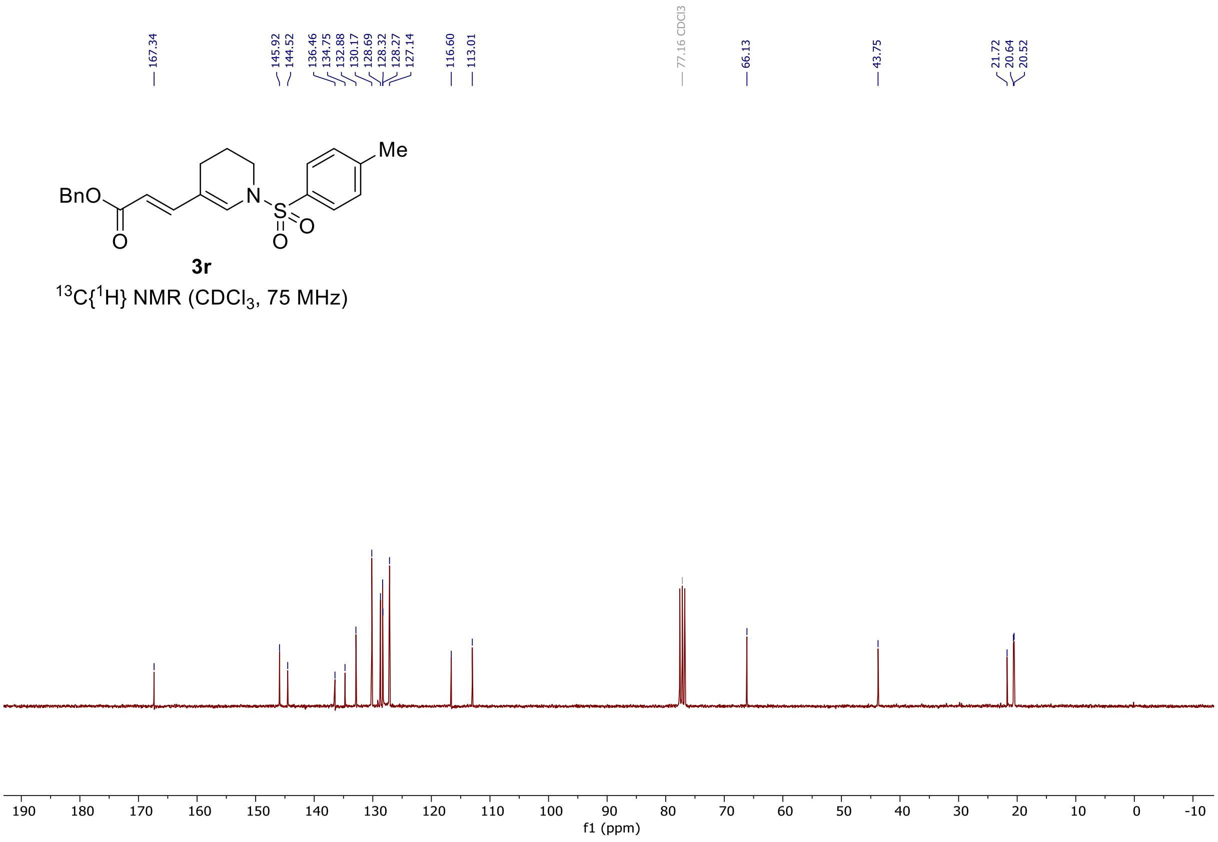

Figure S42

4.23 ^{1}H and ^{13}C{^{1}H} NMR spectra of 3s

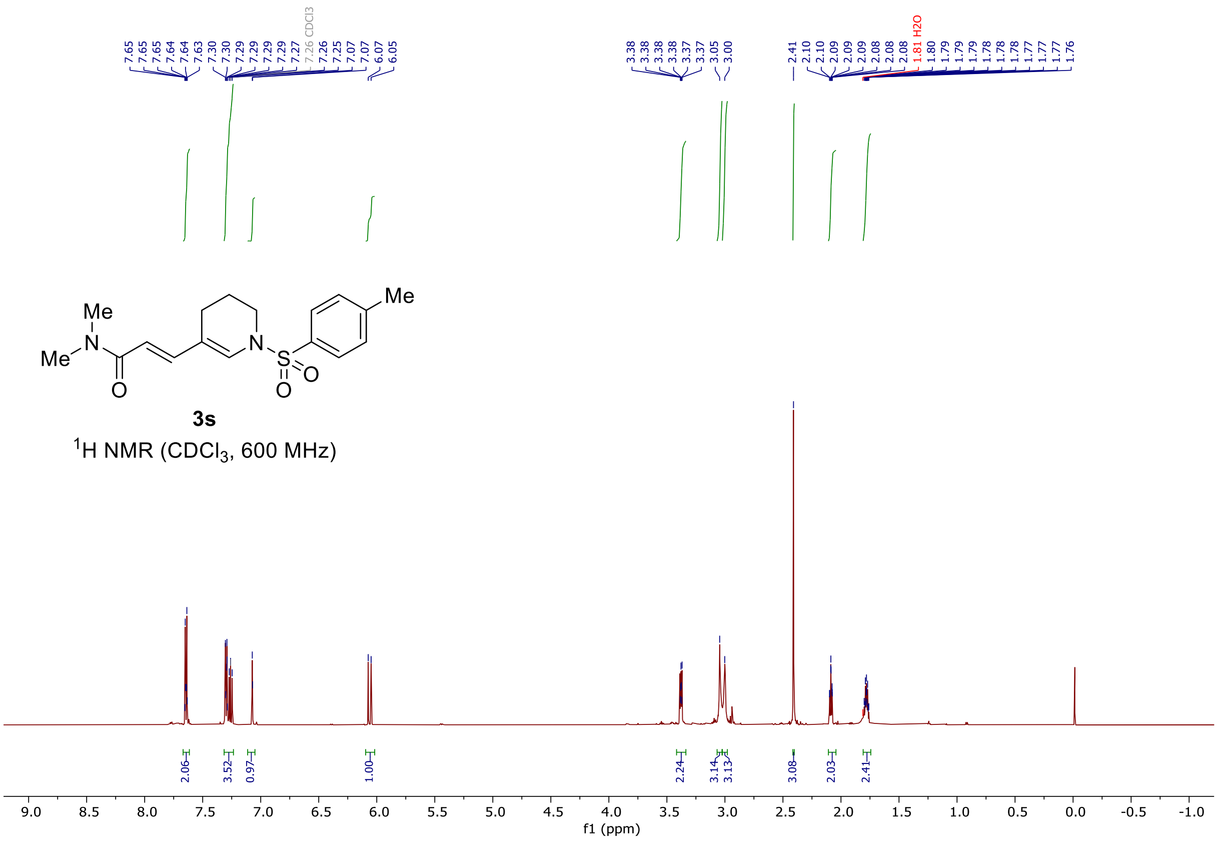

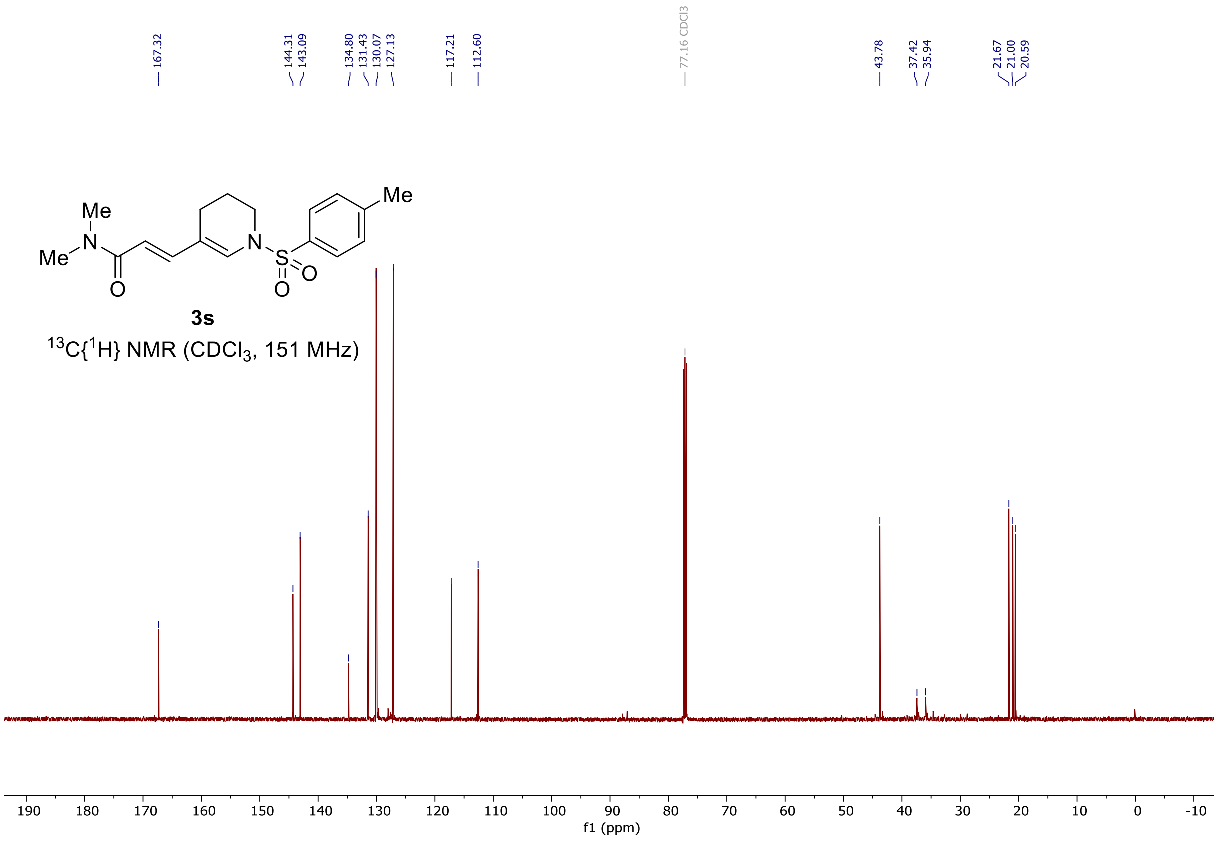

Figure S43

4.24 ^{1}H, $^{13}C\{^{1}H\}$ NMR and ^{31}P spectra of 3t

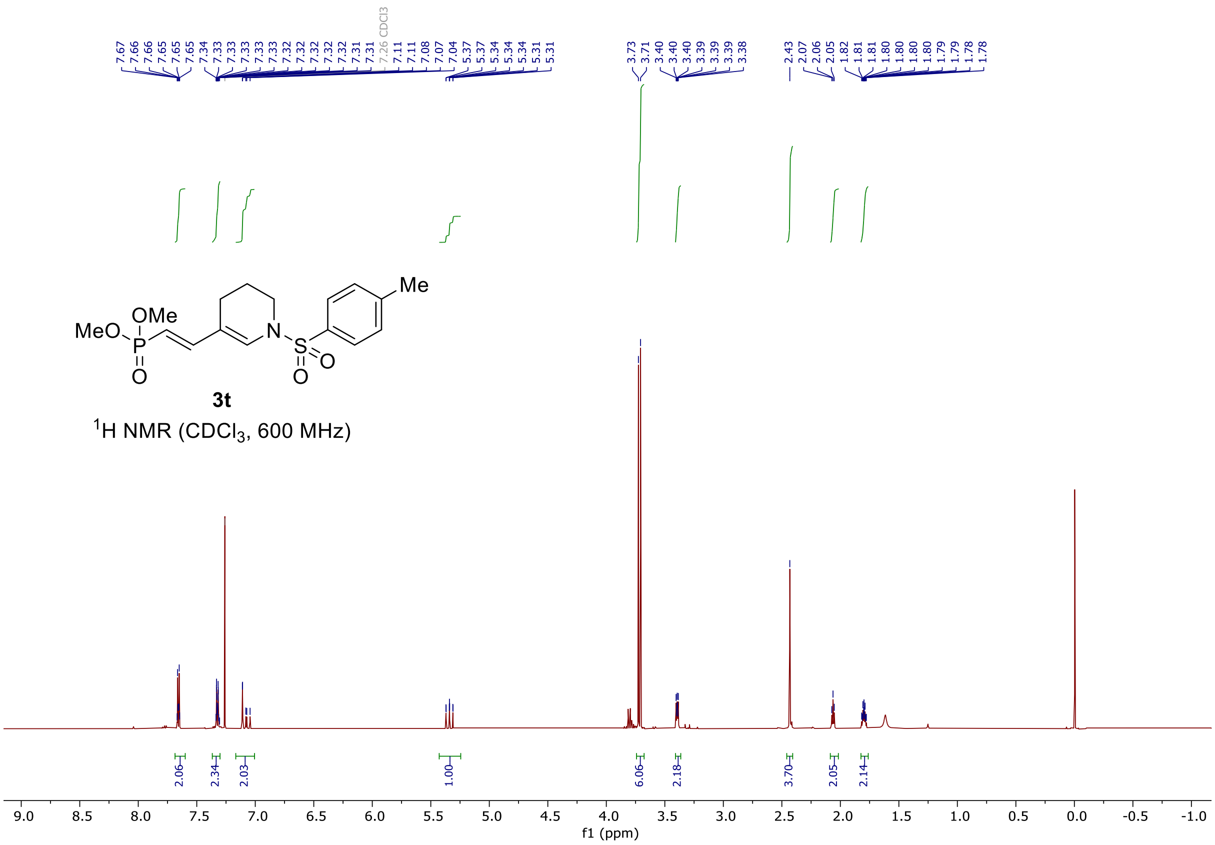

3t

^{1}H NMR ($CDCl_3$, 600 MHz)

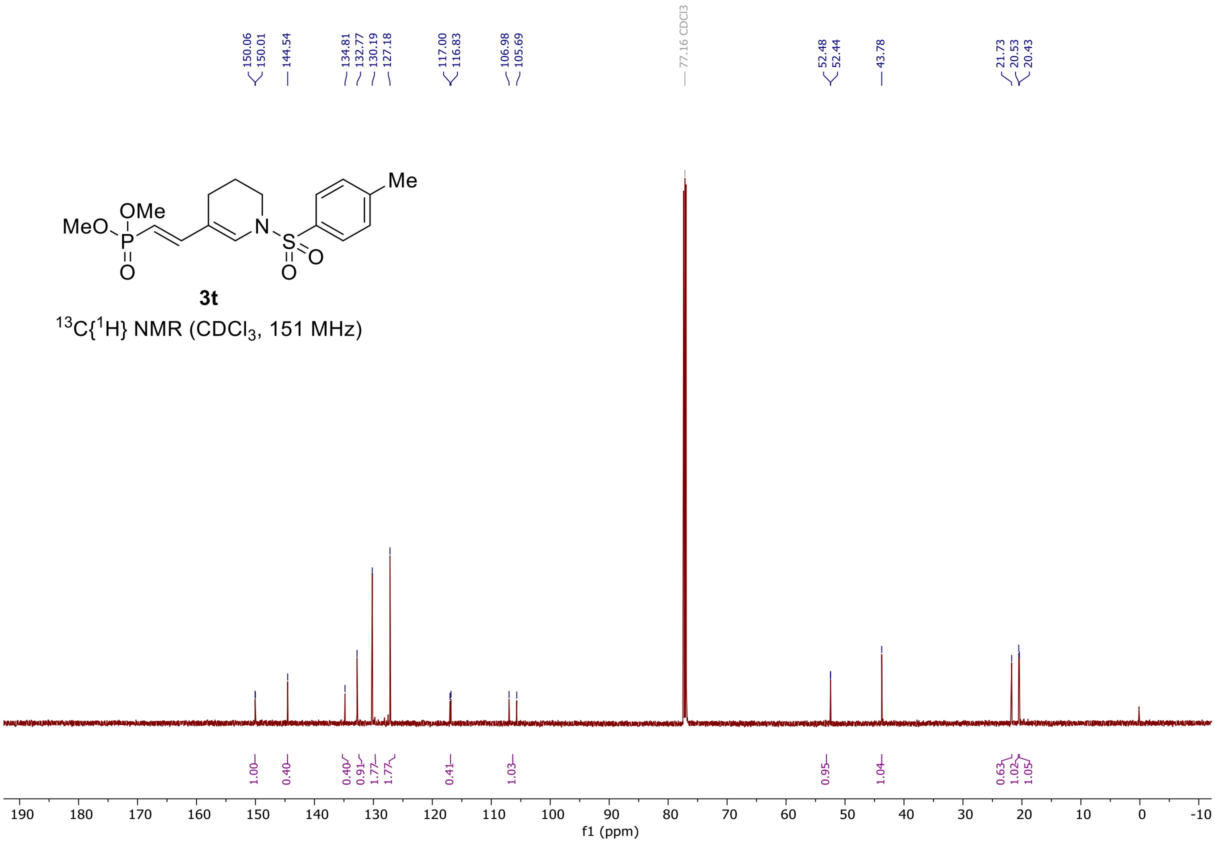

3t

$^{13}C\{^{1}H\}$ NMR ($CDCl_3$, 151 MHz)

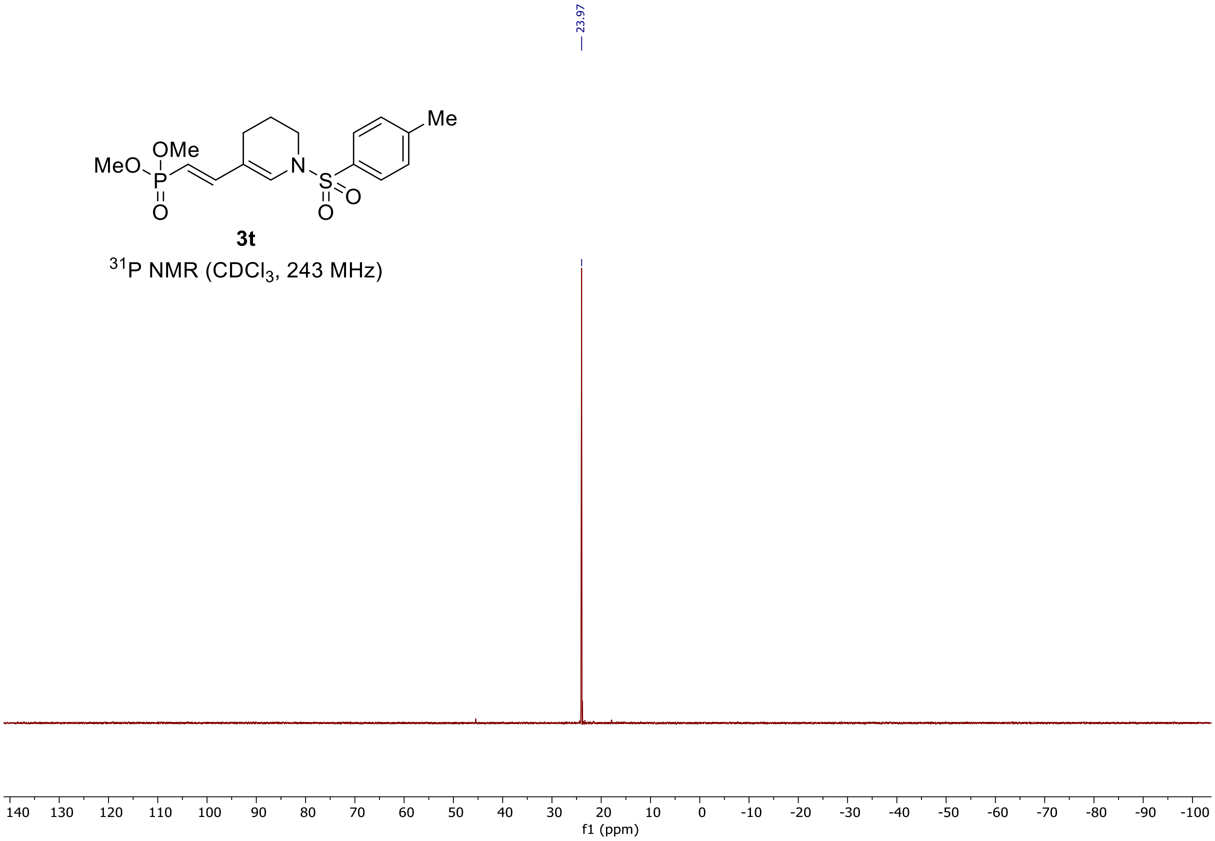

23.97
MeO
OMe
P
O
N
S
O
O
Me
3t
31P NMR (CDCl3, 243 MHz)
140 130 120 110 100 90 80 70 60 50 40 30 20 10 0 -10 -20 -30 -40 -50 -60 -70 -80 -90 -100
f1 (ppm)

Figure S44

4.25 ¹H and ¹³C{¹H} NMR spectra of 3u

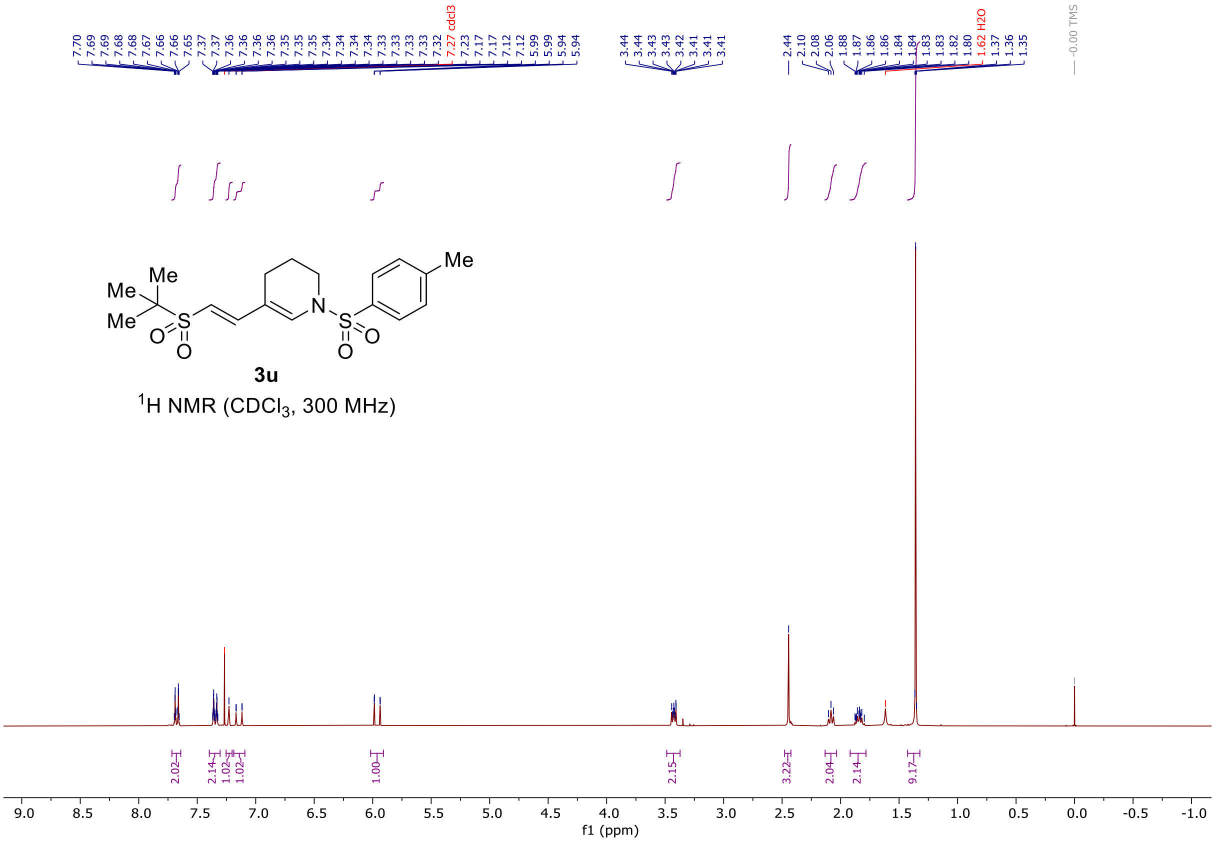

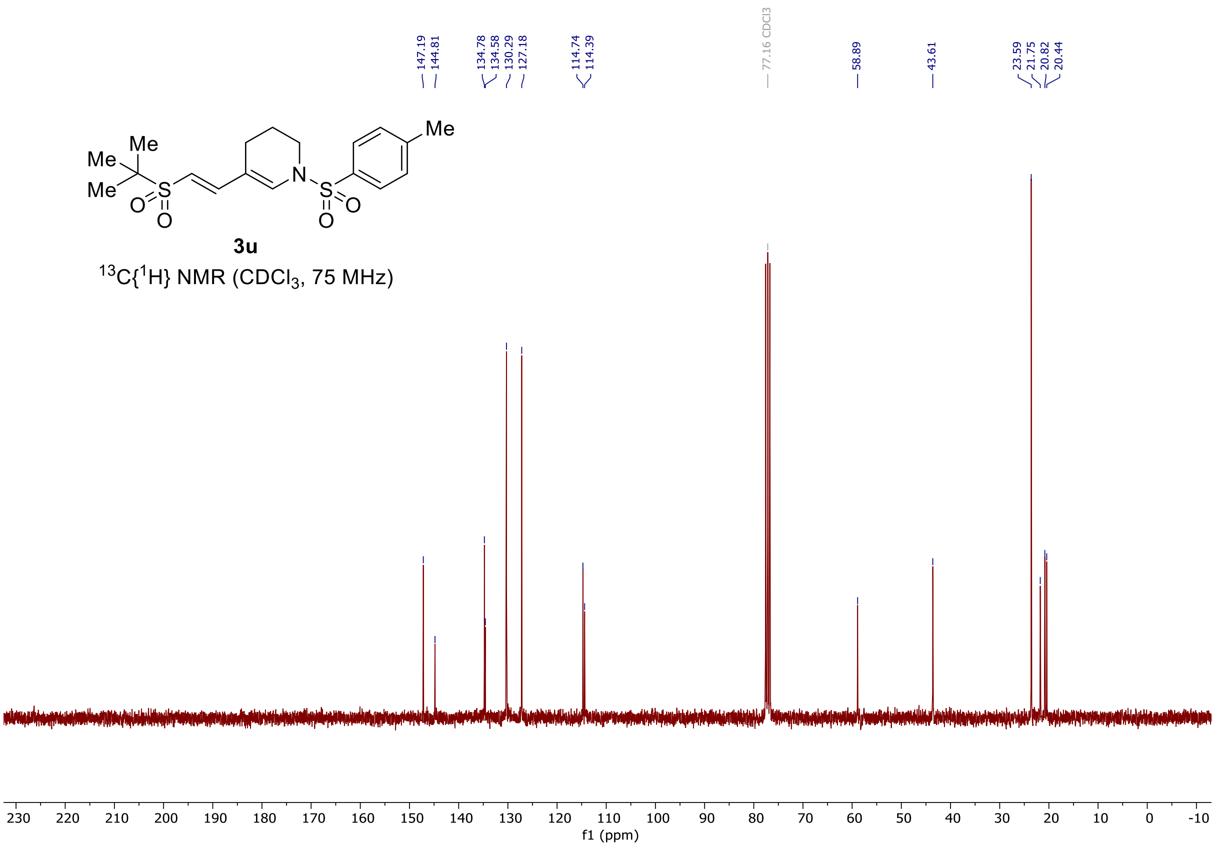

Figure S45

4.26 ^{1}H and ^{13}C{^{1}H} NMR spectra of 5v

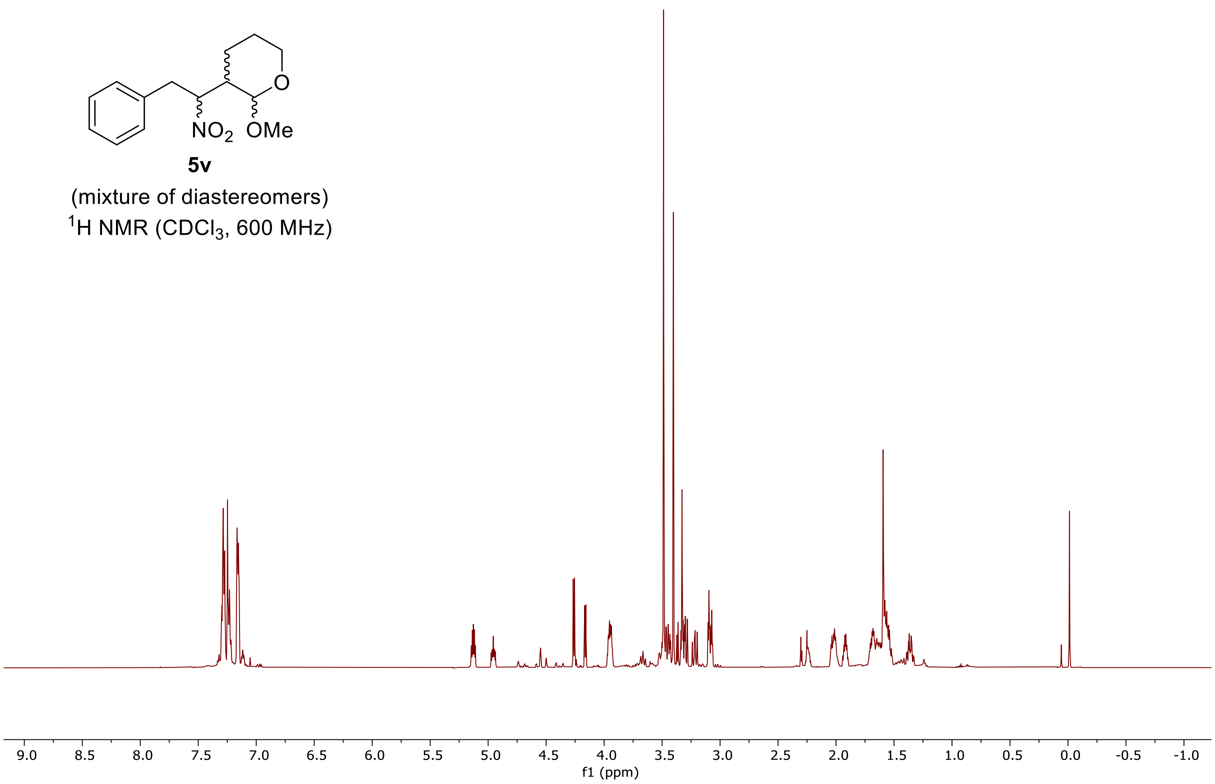

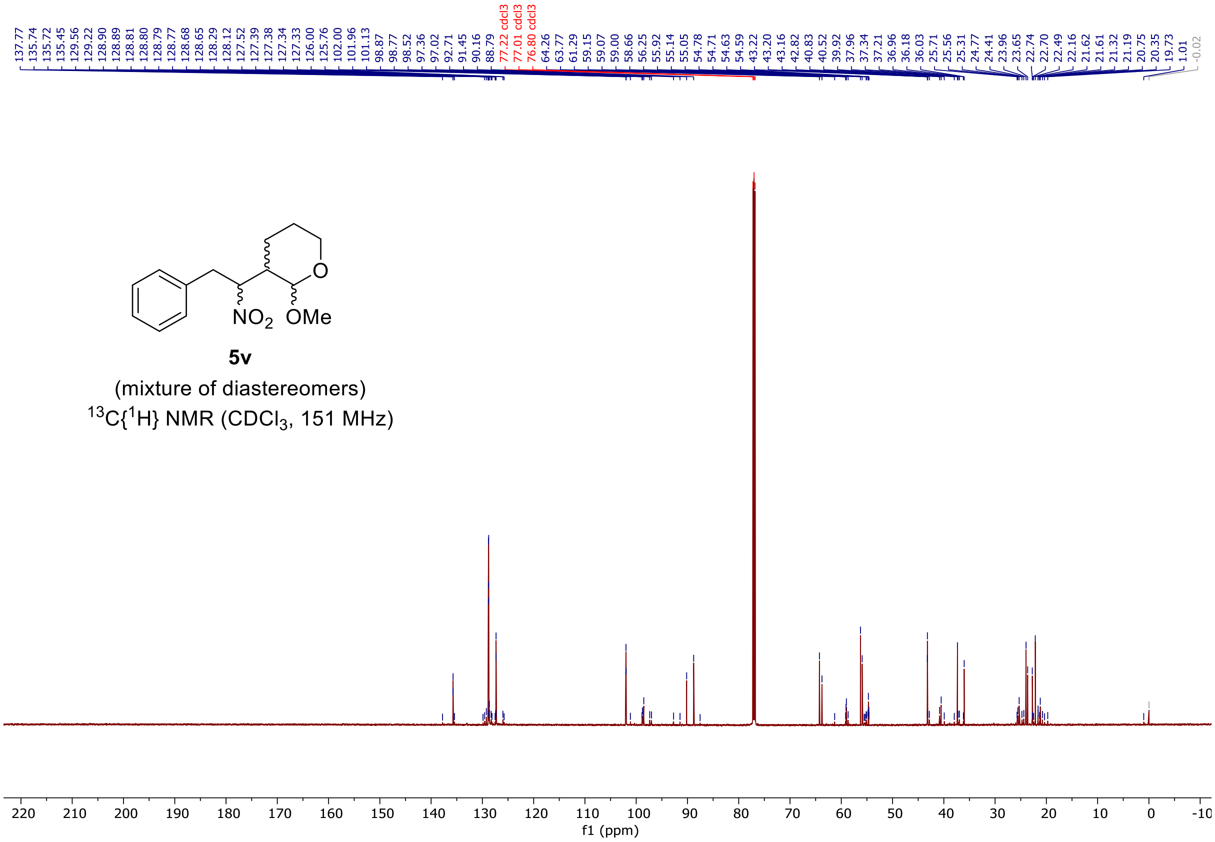

Figure S46

4.27 ^{1}H and ^{13}C{^{1}H} NMR spectra of 16

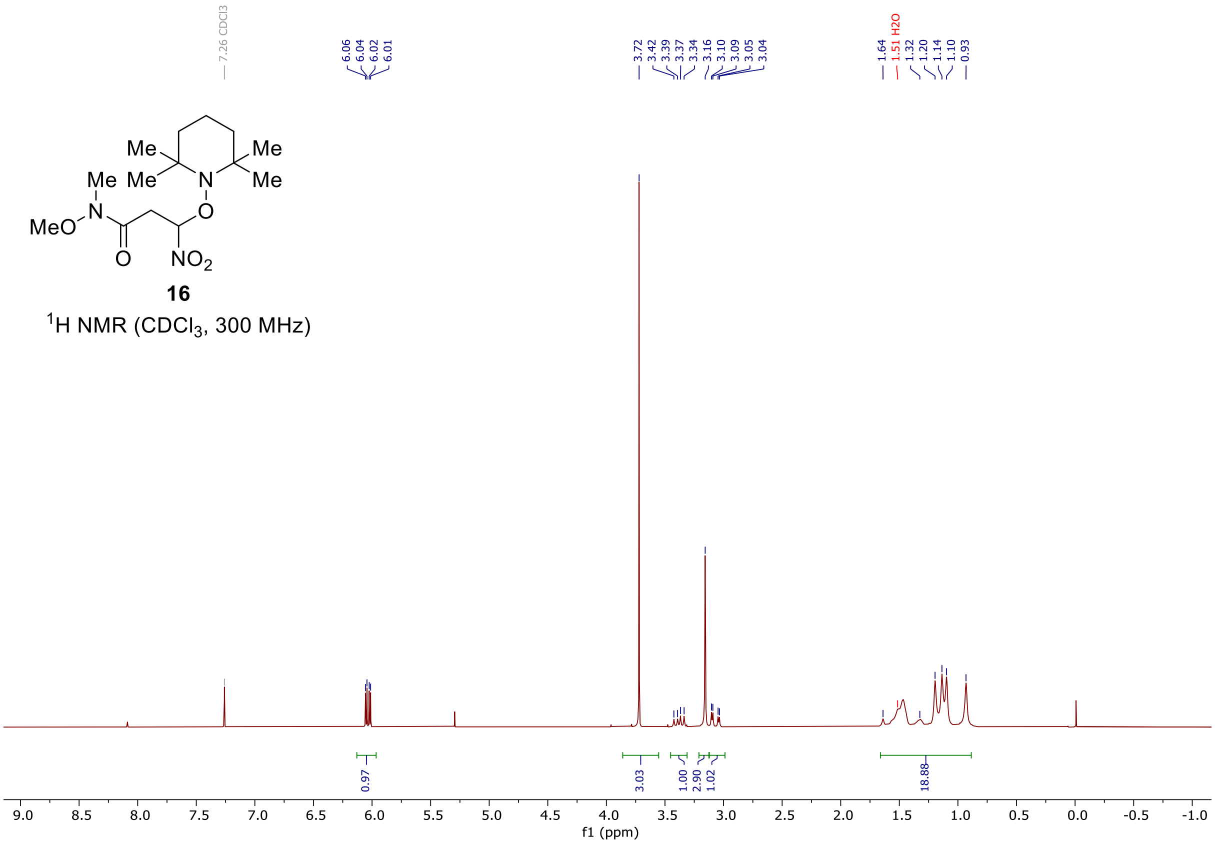

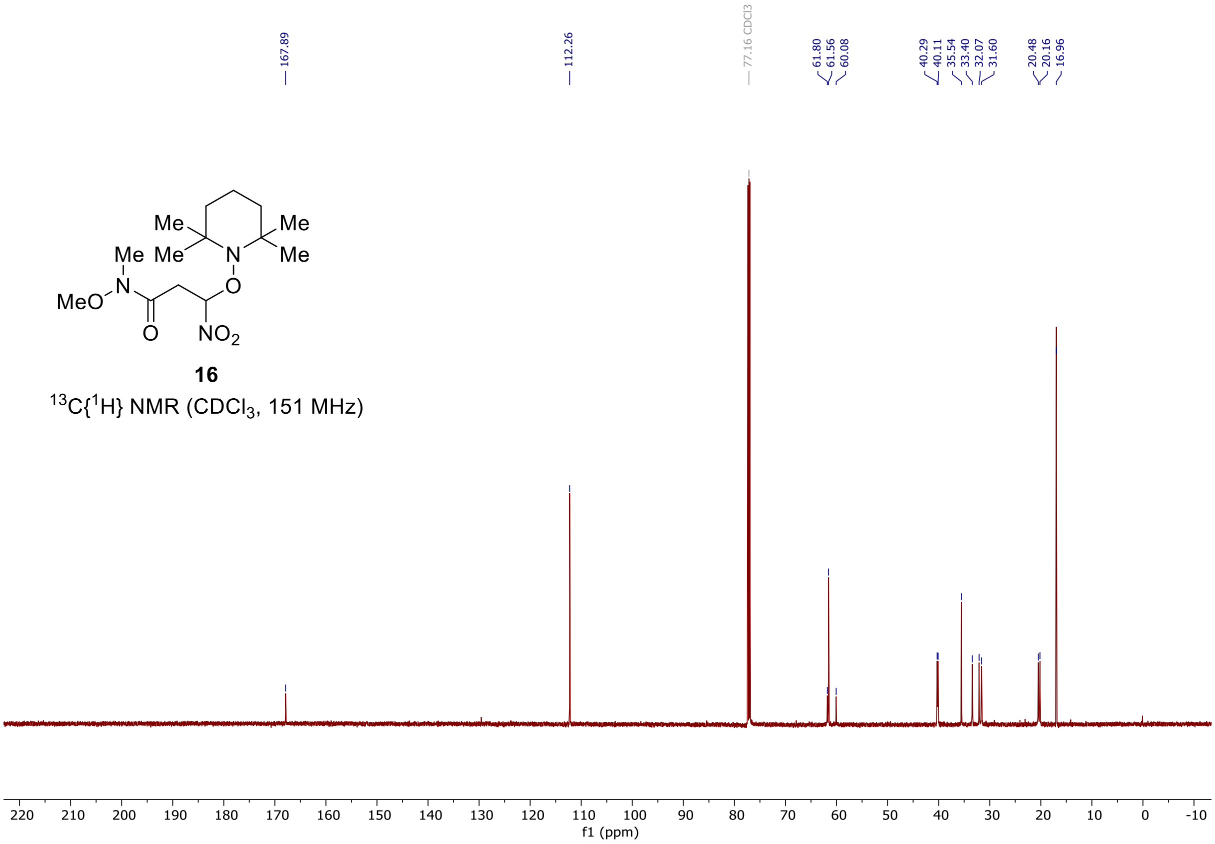

Figure S47

5. References

1. López, R.; Zalacain, M.; Palomo, C. Catalytic Asymmetric Synthesis of γ-Substituted Vinyl Sulfones. *Chem. Eur. J.* **2011**, *17*, 2450–2457, DOI: 10.1002/chem.201003177.

2. Seebach, D.; Henning, R.; Mukhopadhyay, T. Doubly Deprotonated Methyl 3-Mitropropanoate, an Acrylic Ester d^2-reagent. *Chem. Ber.* **1982**, *115*, 1705–1720, DOI: 10.1002/cber.19821150505.

3. Pelletier, S. M.–C.; Ray, P. C.; Dixon, D. J. *Org. Lett.* **2011**, *13*, 6406–6409, DOI: 10.1021/ol202710g.

4. Díaz-Rodríguez, A.; Martínez-Montero, L.; Lavandera, I.; Gotor, V.; Gotor-Fernández, V. *Adv. Synth. Cat.* **2014**, *356*, 2321–2329, DOI: 10.1002/adsc.201400260.

5. Yu, J.; Truc, V.; Riebel, P.; Hier, E.; Mudryk, B. *Org. Synth.* **2008**, *85*, 64–71, DOI: 10.15227/orgsyn.085.0064.

6. Wang, B.; Xiong, D.-C.; Ye, X.-S. Direct C–H Trifluoromethylation of Glycals by Photoredox Catalysis. *Org. Lett.* **2015**, *17*, 5698–5701, DOI: 10.1021/acs.orglett.5b03016.

7. Burchacka, E.; Skoreński, M.; Sieńczyk, M.; Oleksyszyn, J. Phosphonic analogues of glutamic acid as irreversible inhibitors of Staphylococcus aureus endoproteinase GluC: An efficient synthesis and inhibition of the human IgG degradation. *Bioorg. Med. Chem. Lett.* **2013**, *23*, 1412–1415, DOI: 10.1016/j.bmcl.2012.12.074.

8. Bigge, C. F.; Wu, J.-P.; Drummond, J. R. *Tetrahedron Lett.* **1991**, *32*, 7659–7662, DOI: 10.1016/0040-4039(91)80558-N.

9. Zimmerman, H. E.; Wang, P. Inter- and Intramolecular Stereoselective Protonation of Enols[1,2]. *J. Org. Chem.* **2002**, *67*, 9216–9226, DOI: 10.1021/jo026187p.

10. Ranganathan, D.; Rao, C. B.; Ranganathan, S. Nitroethylene: synthesis of novel 2-nitroethylphosphonates. *J. Chem. Soc., Chem. Commun.* **1979**, 975–976, DOI: 10.1039/C39790000975.

11. Paulus, J.; Nachtigall, B.; Meyer, P.; Sewald, N. RGD Peptidomimetic MMAE-Conjugate Addressing Integrin αVβ3-Expressing Cells with High Targeting Index. *Chem. – Eur. J.* **2023**, *29*, e202203476, DOI: 10.1002/chem.202203476.

12. Nagasaka, T.; Hayashim, H.; Hamaguchi, F. Introduction of Alkyl Groups at the a-Positions of Pyrrolidines and Piperidines: Synthesis of (±)-Coniine. *Heterocycles* **1988**, *27*, 1685–1696.

13. Detková, K. D.; Jakubcová, K.; Malatinský, T.; Filo, J.; Cigáň, M.; Budzák, S.; Medved', M.; Jakubec. P. Regioselective C-Arylation of Functionalized Nitroalkanes with Furan, Thiophene, and Substituted Thiophenes. *J. Org. Chem.* DOI: 10.1021/acs.joc.5c02410.
